# Uncovering a Novel Role of ROR1 in the Epigenetic Regulation of Tumor Suppressor Gene CREB3L1 in Triple-Negative Breast Cancer Cells

**DOI:** 10.3390/biom15050734

**Published:** 2025-05-16

**Authors:** Victoria L. Reed, Eric Lalu, Leena Yoon, Norman Fultang, Bela Peethambaran

**Affiliations:** 1 Department of Biology, St. Joseph’s University, Philadelphia, PA 19131, USA; vr20634074@sju.edu (V.L.R.); el20840346@sju.edu (E.L.); ly10782859@sju.edu (L.Y.); 2 Cancer Biology Program, The Wistar Institute, Philadelphia, PA 19104, USA; 3 Perelman School of Medicine, University of Pennsylvania, Philadelphia, PA 19104, USA; norman.fultang@pennmedicine.upenn.edu

**Keywords:** epigenetic regulation, tumor suppressor genes, CpG methylation, TNBC, ROR1, CREB3L1, DNA methyltransferases

## Abstract

A characteristic of triple-negative breast cancer (TNBC) is the epigenetic regulation of tumor suppressor genes, leading to TNBC heterogeneity and treatment resistance in patients. TNBC exhibits high methylation rates, leading to the silencing of numerous tumor suppressor genes. DNA methyltransferase inhibitors (DNMTis) have shown limited clinical efficacy in TNBC treatment. This study aims to uncover a target that could be used to reverse the epigenetic silencing of tumor suppressor genes in TNBC. The Western blot analysis demonstrated that ROR1 knockdown, an oncofetal gene, reduced DNMT3A and DNMT3B protein expression in the TNBC cell lines MDA-MB-231 and HCC1806, as well as a non-malignant breast cell line, MCF10A. The reduced representation bisulfite sequencing (RRBS) analysis identified differential methylation of CREB3L1 when ROR1 is knocked down in TNBC cell lines. CREB3L1 is a transcription factor that plays tumor-suppressive roles in TNBC and is commonly epigenetically silenced in patients. This study shows that ROR1 requires pSTAT3 activation to upregulate DNMT3A and DNMT3B expression to induce CREB3L1 epigenetic silencing in TNBC. ROR1 knockdown resulted in the re-expression of CREB3L1 in TNBC cells. The data provide evidence that ROR1 inhibition, in combination with DNMTis, could enhance patient outcomes as a therapeutic approach for TNBC.

## 1. Introduction

In the United States, breast cancer is the most frequently diagnosed malignancy and ranks as the second leading cause of cancer-related deaths among women [1]. There are four main subtypes of breast cancer: luminal A, luminal B, human epidermal growth factor receptor 2 (HER2) positive, and triple-negative [2]. Triple-negative breast cancer (TNBC) lacks the three main receptor treatment targets (estrogen, progesterone, and HER2), leading to rapid proliferation of tumor cells, early metastasis, and treatment resistance [3,4]. Although chemotherapy is the standard approach to treating TNBC, tumor recurrence and poor prognosis are common due to the intrinsic chemotherapy resistance of TNBC [5,6]. There is a dire need to identify prognostic markers that aid in the development of efficient TNBC treatments, specifically targeting cancer cells in patients.

TNBC is a heterogeneous disease with several histological and molecular alterations within the tumor tissue [3]. TNBC is composed of six different subtypes that display unique biological processes. This increases the difficulty of targeting and killing TNBC, since cells within the different TNBC subtypes drive disease progression through the activation of different signaling pathways [3]. Treatment failure and disease progression are a result of complex tumor–host interactions, such as alterations in the tumor genome and immune microenvironment [7]. One of the driving forces underlying tumor heterogeneity, therapeutic resistance, and the progression of TNBC is epigenetic dysregulation, such as oncogene activation and tumor suppressor gene dysfunction [4,8]. The epigenetic silencing of tumor suppressors mediated by oncogenes has been demonstrated in several malignancies, including breast cancer [9]. DNA hypermethylation is a process in which methyl groups are transferred from the methyl donor, S-adenosylmethionine, and added to the fifth carbon position of cytosine bases within CpG-rich promoter regions [10,11]. This process is a prevalent epigenetic modification seen in TNBC [4,10,12]. DNA methylation is facilitated by DNA methyltransferases (DNMTs) such as DNMT1, DNMT3A, and DNMT3B, all of which are overexpressed in breast cancer patients with advanced clinical disease stages [10,13]. DNMT3A and DNMT3B are known as the de novo methyltransferases, which establish the methylation of CpG sites, and DNMT1 is known as the “maintenance methyltransferase” since it sustains the methylation patterns during cell proliferation [10]. DNMT3A and DNMT3B have also been found to aid in maintaining methylation patterns as well [14,15]. The high expression of DNMT1, DNMT3A, and DNMT3B correlates with low overall survival rate, increased tumor size, and lymph node metastasis in TNBC patients [4,10,16]. This is a result of the DNMT-mediated hypermethylation of the promoter regions within tumor suppressors, leading to the epigenetic silencing of these genes and enhancing epithelial-to-mesenchymal transition (EMT), proliferation, survival, and metastasis of TNBC [4,12].

Decitabine and azacytidine are FDA-approved DNMT inhibitors for the treatment of hematological malignancies, such as myelodysplastic syndromes and myeloid leukemia, but efficacy in treating solid tumors has been lackluster [17]. In TNBC clinical trials (NCT01349959, NCT03295552, NCT00748553), decitabine and azacytidine have failed to demonstrate significant clinical benefit. These treatment failures are thought to be due, in part, to TNBC heterogeneity, preventing the identification of a universal target for the epigenetic dysregulation seen in TNBC. Additionally, DNMT inhibitors cause off-target cytotoxicity in patients due to their lack of specificity for oncogenic DNMTs [18]. This underscores the need to identify a gene that is specifically upregulated in cancerous tissue and can be used to selectively inhibit oncogenic DNMTs without affecting their physiological roles in healthy cells.

Receptor tyrosine kinase-like orphan receptor 1 (ROR1) is a transmembrane protein with low expression in healthy tissue but is highly expressed in embryonic development and cancer [19,20]. ROR1 is activated by its ligand, Wnt5a, which triggers the auto- or hetero-phosphorylation of its tyrosine residues [21]. Breast cancer patients with high ROR1 expression are seen to have significantly shorter metastasis-free and overall survival rates compared to those with low ROR1 expression [22]. ROR1 is thought to be an attractive biomarker in breast cancer, primarily for TNBC and its subtypes, and increases metastasis by activating various oncogenic signaling pathways, including PI3K/AKT, Hippo, and MEK/ERK pathways [23,24,25]. We have previously shown that ROR1 regulates chemoresistance in TNBC through the regulation of drug-efflux pump ABCB1 [26]. Inhibiting ROR1 activation by treatment with strictinin, a small molecule inhibitor, resulted in the downregulation of the PI3K/AKT pathway, thus decreasing tumor cell migration and survival and increasing apoptosis [27]. There is a lack of comprehensive understanding of an upstream oncogene that controls DNMT regulation in TNBC. Evidence has shown that the PI3K/AKT pathway mediates epigenetic regulation in cancer through DNMT1 and DNMT3B stabilization and upregulation in cancers such as glioblastoma and leukemia [28,29]. However, there has been no connection established between ROR1, the upstream activator of the PI3K/AKT pathway, and DNMT regulation in TNBC. We employed ROR1 knockdown in a TNBC cell line and screened the differentially methylated regions to identify functionally silenced tumor suppressor genes. The data presented in this study further characterized the role of ROR1 in TNBC by identifying a novel function of ROR1 as an epigenetic regulator of tumor suppressors.

Hypermethylation, unlike genetic mutations, can be reversed, resulting in the re-expression of these genes [4]. Therefore, we must identify tumor suppressor genes that are only silenced through hypermethylation, not mutations, in TNBC. cAMP responsive element binding protein 3-like-1 (CREB3L1) is a transcription factor for the unfolded protein response (UPR) that is activated through intramembrane proteolysis upon ER stress [30]. CREB3L1, originally known as an old astrocyte specifically induced substance (OASIS), has been known to play important roles in bone formation and astrocyte differentiation [31,32,33]. CREB3L1 plays a tumor-suppressive role in breast cancer: prolonged CREB3L1 expression under cytotoxic conditions induces apoptosis as opposed to favoring tumor cell survival and has been found to negatively regulate various oncogenes that enhance breast cancer development [30]. Chemotherapeutic drugs, such as doxorubicin and paclitaxel, have been shown to activate CREB3L1, leading to TNBC growth inhibition and cell cycle arrest [34,35]. *CREB3L1* is epigenetically silenced through DNA hypermethylation, a step that is also necessary for Hepatitis C-infected cell proliferation [36]. Low *CREB3L1* expression is associated with high-grade metastatic breast cancers with poor prognosis, specifically TNBC [33]. In this report, we address current gaps in the literature by identifying ROR1 as a key driver of DNMT regulation and CREB3L1 silencing in TNBC. We show that knocking down ROR1 protein levels decreases the hypermethylation of CpG regions within the *CREB3L1* gene, thus reversing its inhibition, resulting in the re-expression of the gene. Our results establish a pathway suggesting ROR1 as a potential therapeutic target for reversing the epigenetic silencing of tumor suppressor genes in TNBC and other malignancies that have low *CREB3L1* expression.

## 2. Materials and Methods

### 2.1. Cell Culture and Treatment Techniques 

#### 2.1.1. Cell Culture

TNBC cell lines MDA-MB-231 (HTB 26, ATCC, Manassas, VA, USA), HCC1806 (CRL-2335, ATCC, Manassas, VA, USA), and normal human epithelial breast cell line MCF10A (CRL-10317, ATCC, Manassas, VA, USA) are cultured as recommended by ATCC. MDA-MB-231 and HCC1806 represent the mesenchymal stem-like and basal-like 2 subtypes, respectively [37]. MDA-MB-231 cells were cultured in DMEM/F-12 (10-090-CV, Corning, NC, USA) supplemented with fetal bovine serum (FBS) (10%), insulin (10 µg/mL sc-360248, Santacruz, CA, USA ), non-essential amino acids, sodium pyruvate (1 mM, S8636, Sigma, MO, USA ), penicillin (100 U/mL, 30-001-C1, Sigma, MO, USA), and streptomycin (0.1 mg/mL, 30-001-C1, Sigma, MO, USA). HCC1806 cells were cultured in RPMI (10-040-CV, Corning, NC, USA), with FBS (10%, Waltham, MA, USA) insulin (10 ug/mL, sc-360248, Santacruz, CA, USA), penicillin (100 U/mL, Sigma, St. Louis, MO, USA), and streptomycin (0.1 mg/mL, Sigma, St. Louis, MO, USA). The cells were incubated in 5% CO_2_ as recommended by ATCC. MCF10A cells were cultured in DMEM/F12K (Corning, NC, USA) with FBS (5%, CPS Serum, MO, USA), insulin (10 µg/mL, Santacruz, CA, USA), hydrocortisone (0.5 mg/mL, Waltham, MA, USA), EGF (20 ng/mL), penicillin (100 µg/mL, Sigma, MO, USA), and streptomycin (0.1 mg/mL, Sigma, St. Louis, MO, USA).

#### 2.1.2. Cell Transfection Techniques

To induce an ROR1 knockdown, the TNBC cell lines MDA-MB-231 and HCC1806 were transfected with an ROR1-specific siRNA (siROR1) (Thermo Fisher Scientific, 4390824, Waltham, MA, USA) using Lipofectamine^TM^ 3000 (Thermo Fisher Scientific, L3000008, Waltham, MA, USA). A total of 400,000 cells were seeded in each well of a 6-well plate (Thermo Fisher Scientific, 353046, Waltham, MA, USA) and treated with siROR1 and Opti-MEM reduced serum (Thermo Fisher Scientific, 31985088) for 24 h. The cells were rescued for 24 h, and then cells were lysed for protein extraction or further experimentation. The control cells were treated using scrambled RNA (scRNA) (Thermo Fisher Scientific, AM4611, Waltham, MA, USA).

To induce a STAT3 knockdown, the HCC1806 TNBC cells were transfected with a STAT3-specific siRNA (siSTAT3) (Thermo Fisher Scientific, 4390824, Waltham, MA, USA) using Lipofectamine^TM^ 3000. A total of 400,000 cells were seeded in each well of a 6-well plate and treated with siSTAT3 and Opti-MEM reduced serum for 24 h. The cells were rescued for 24 h, and then cells were lysed for protein extraction or further experimentation. The control cells were treated using scRNA. To determine the effects of STAT3 knockdown, Western blotting was conducted to probe the levels of STAT3, phosphorylated-STAT3 (pSTAT3), ROR1, and CREB3L1 using the antibodies mentioned under Section 2.2.

#### 2.1.3. Cell Treatment Techniques

To enhance STAT3 expression in normal breast cells, the MCF10A cells were seeded in a 6-well plate (approximately 450,000 cells per well). Interleukin-6 (IL-6) is a mediator of pSTAT3 activation; thus, after 24 h, the cells were treated with 100 ng/mL of human IL-6 recombinant protein (Cell Signaling, 48333S, London, UK) for 30 min. The naive MCF10A cells were used as the control sample. Following incubation, the cells were washed with 1×PBS and lysed using RIPA buffer (as described in Section 2.2).

### 2.2. Protein Extraction and Western Blot Analysis

The cells were rinsed once with 1× PBS, scrapped from each well, and then lysed using 100 µL of RIPA buffer (150 mM NaCl, 1.0% Triton-X-100, 0.5% sodium deoxycholate, 0.1% SDS, 50 mM Tris, pH 8.0) containing 1% protease inhibitor and EDTA (Thermo Fisher Scientific, 78410, Waltham, MA, USA) per sample. Protein concentrations were determined by Bradford Assay using Pierce660 and protein standards (siSTAT3) (Thermo Fisher Scientific, 23200, Waltham, MA, USA). A total of 20 µg of protein was used in SDS-PAGE. Next, the gel was transferred to a nitrocellulose membrane using the iBlot transfer device (Thermo Fisher Scientific, IB301031, Waltham, MA, USA). The membranes were blocked in 5% BSA and then incubated for one hour in primary antibody overnight at 4 °C. The membranes were washed 3 times with 1×TBS-T (20 mM Tris, 150 mM NaCl, 0.1% Tween 20). Membranes were incubated at room temperature for one hour with horseradish-peroxidase-linked Iggs. The protein bands were imaged using Western ECL substrate (Bio-Rad, 170-5060, California, CA, USA) and visualized using chemiluminescence on the ChemiDoc Imaging System (Bio-Rad, 12003153). The primary antibodies were as follows: ROR1 (Cell Signaling, 16540, 1:1000, Cambridge, UK), CREB3L1 (Protein Tech, 11235-AP, 1:2000, Rosemont, IL, USA), DNMT3A (Cell Signaling, 32578, 1:1000, Cambridge, UK), DNMT3B (Cell Signaling, 57868, 1:1000, Cambridge, UK), DNMT1 (Cell Signaling, 5032, 1:1000, Cambridge, UK), STAT3 (Cell Signaling, 12640, 1:1000, Cambridge, UK), pSTAT3 (Cell Signaling, 9145, 1:1000, Cambridge, UK), and β-actin (Santa Cruz, sc-47778, 1:1000, Dallas, TX, USA). The secondary antibodies were as follows: anti-rabbit (Bio-Rad, 1706515, 1:3000) and anti-mouse (Bio-Rad, 1706516, 1:3000, Philadelphia, PA, USA). The protein expression was calculated using Fiji software (Version: 2.16.0/1.54p), first quantifying the signal of the protein of interest, and then normalizing it to the signal of the loading control, β-actin. The statistical analysis was calculated using a paired *t*-test. Three technical replicates were used for each protein of interest probed in the Western blot experiments.

### 2.3. DNMT Activity Assay

Nuclear protein was extracted from MDA-MB-231 and HCC1806 TNBC cells using a nuclear extraction kit (Abcam, ab113474, Cambridge, UK). The cytoplasmic extract was removed from the nuclear material by lysing the TNBC cells using 100 µL of 1X pre-extraction buffer. The nuclear protein was then extracted using 10 µL of extraction buffer. We used 20 µg of protein in each test. The DNMT3A activity (Abcam, ab113470, Cambridge, UK) was tested in MDA-MB-231 cells, and the DNMT3B activity (Abcam, ab113471, Cambridge, UK) was tested in HCC1806 cells. The MDA-MB-231 and HCC1806 cells were seeded at 400,000 cells per well in a 6-well plate. For the control sample, we treated MDA-MB-231 and HCC1806 cells with scrambled RNA. We knocked down ROR1 expression in the same cell lines by treating the cells with siRNA. Following the nuclear protein extraction, the control and ROR1 knockdown sample lysates were added to the wells provided in the kit. The lysates were incubated in the wells for 2 h to allow for the DNMTs in our samples to bind to the DNA affinity substrate that was coated on the bottom of the wells. The bound DNMTs are incubated in DNMT-specific antibodies and then quantified using a SpectraMax^®^ ABS Plus absorbance microplate reader (Molecular Devices, San Jose, CA, USA). The absorbance was read at 450 nm, and the reference wavelength was read at 655 nm. The statistical analysis was calculated using a paired *t*-test. Three technical replicates were used for each protein of interest probed in the Western blot experiments.

### 2.4. Immunofluorescence Analysis

A total of 50,000 cells were seeded in 35 mm glass-bottom dishes (Cellvis, D35-10-1.5-N, Mountain View, CA, USA) and fixed using 300 µL of ice-cold methanol for 5 min at −20 °C. After a series of PBS washes, cells were incubated overnight at 4 °C in 200 µL of primary antibody diluted in normal goat serum and PBS. The cells were washed again and then incubated for one hour at 37 °C in 200 µL of a secondary antibody. Following incubation, cells were washed, and then 100 µL of DAPI stain solution was added to the cells for 3 min at room temperature. The cells were PBS-washed twice and then imaged via the STELLARIS 8 laser-scanning confocal microscope (Leica Microsystems, Wetzlar, DE). The control for each experiment included MDA-MB-231 and HCC1806 cells treated with scrambled RNA. We knocked down the ROR1 in the same cell lines using siRNA. The quantification of the images was conducted using Image J analysis. The primary antibodies were as follows: CREB3L1 (Protein Tech, 11235-AP, 1:1000, Rosemont, IL, USA), DNMT3A (Cell Signaling, 32578, 1:1000, Cambridge, UK), and DNMT3B (Cell Signaling, 57868, 1:1000, Cambridge, UK). The secondary antibody used was anti-rabbit Alexa-Fluor-488 (Thermo Fisher Scientific, A-11008, 1:1000, Waltham, MA, USA). Fiji software was used to measure the nuclear fluorescence intensity for the proteins of interest. The nuclear fluorescence intensity was calculated using the Corrected Total Cell Fluorescence (CTCF) calculation, subtracting the background fluorescence readings and correcting for area differences. The statistical significance was calculated using a paired *t*-test from 30 technical replicate images in both the control and the ROR1 knockout cells.

### 2.5. Chromatin-Immunoprecipitation (ChIP)

The DNMT-bound chromatin was immunoprecipitated using the Pierce Agarose ChIP Kit (Thermo Fisher, 26156, Waltham, MA, USA). The cells were seeded at 2 million cells per 6-well plate. For the control samples, we treated MDA-MB-231 and HCC1806 cells with scrambled RNA. We treated the same cell lines with siRNA to knock down ROR1 expression. Following transfection, the cells were fixed with 1% paraformaldehyde. Cells were lysed, and nuclear chromatin-bound protein was extracted. Immunoprecipitation was performed overnight at 4 °C using DNMT3A (Cell Signaling, 57868, 1:100, Cambridge, UK) or DNMT3B (Cell Signaling, 57868T, 1:50, Cambridge, UK) antibodies. CREB3L1 levels were analyzed using the CFX384 Touch Real-Time PCR detection system (Bio-Rad, 1855484), 5 ng of CREB3L1 DNA, SYBR Master Mix Hi-ROX (Origene, QP100001, Rockville, MD, USA), and a CREB3L1-specific primer (Origene, HP216486, Rockville, MD, USA) (Fwd—CCTTGTGCTTTGTTCTGGTGC/Rev—CCGTCATCGTAGAATAGGAGGC). GAPDH was used as the reference gene (Thermo Fisher Scientific, 1862245, Waltham, MA, USA). The statistical significance was calculated using a paired *t*-test from 30 technical replicate images in both the control and the ROR1 knockout cells. The following cycle conditions were used: 95 °C for 15 min, 95 °C for 15 s, and 62 °C for 1 min (steps 2 and 3 repeated for 40 cycles).

### 2.6. DNA Extraction and Reduced Representation Bisulfite Sequencing

The MDA-MB-231 cells were treated with scrambled RNA in our control samples. We knocked down ROR1 expression in MDA-MB-231 cells through siRNA transfection. DNA was extracted from the samples using the DNeasy Blood and Tissue kit (Qiagen, 69504, Hilden, Germany) as described in the manufacturer’s protocol and sent to Zymo Research to perform bisulfite sequencing analysis. To prepare the RRBS library, 400 ng of input DNA was digested with 30 units of MspI (NEB). The DNA fragments were ligated to pre-annealed adapters containing 5’-methyl-cytsoine instead of cytosine according to Illumina’s guidelines. The adaptor-ligated fragments greater than or equal to 50 base pairs in size were recovered using the DNA Clean and Concentrator-5 (Zymo, D4003, Irvine, CA, USA). The fragments then were bisulfite-treated using the EZ DNA Methylation-Lightning Kit (Zymo, D5030, Irvine, CA, USA). Preparative-scale PCR was performed, and the resulting products were purified with DNA Clean and Concentrator-5 for sequencing on the Illumina NovaSeq 6000 platform (150 base pairs PE reads). The sequence reads from bisulfite-treated classic RRBS libraries were identified using standard Illumina base-calling software to perform sequence alignments and data analysis. Raw FASTQ files were adapter, filled-in nucleotides, and quality-trimmed using TrimGalore. FASTQC was used to assess the effect of trimming and overall quality distributions of the data. Alignments to reference genomes were performed using Bismark. Methylated and unmethylated read totals for each CpG site were called using MethylDackel 0.5.0. The methylation level of each sampled cytosine was estimated as the number of reads reporting a C, divided by the total number of reads reporting a C or T. The cytosines with read depth ≥ 5 in ≥ 1 samples in a group were kept or otherwise removed from that group before downstream analyses. The differentially methylated cytosines (DMCs) and differentially methylated regions (DMRs) were detected with *dss*. The significant DMCs and DMRs have *FDR* ≤ *0.05* (if *p* values are provided by statistical method) and *absolute methylation difference* ≥ *0.1*. The results were summarized using MultiQC v1.9. Each DMR was annotated by overlapping its genomic region with other functional regions (including genes, exons, introns, promoters, and CpG islands), derived from The University of Santa Cruz (UCSC) Genome Browser (RefSeq ID: NM_052854.4.). The overlapped genes identified were put into g:Profiler to produce the functional enrichment analysis. Heatmap plots were generated to visualize the DMR results in the control and siROR1 samples. The methylation score was calculated using the DMCs from the control and siROR1 samples and the statistical significance was calculated using the Wilcoxon test. The raw data have been published on Zenodo (https://zenodo.org/records/15236296), 17 April 2025.

### 2.7. Statistical Analysis

The raw data were processed on Google Sheets before being uploaded to GraphPad Prism 10 (GraphPad Software Inc, San Diego, CA, USA). The statistical significance between the control cells treated with scRNA and the cells with ROR1 knockdown were analyzed using a two-tailed paired Student’s *t*-test for all experiments unless described differently in the figure legend. For the statistical analysis and visualization, we used TrackViewer, a Bioconductor package, to generate the methylation score box plot and the lollipop plot from the RRBS results. The box plots were created in Python, version 3.13.3 using the seaborn and matplotlib libraries, and the Wilcoxon test was performed to assess the statistical significance of the DMR methylation score. Fiji software (https://doi.org/10.1038/nmeth.2019, accessed on 17 April 2025) [38] was used to quantify Western blot and immunofluorescence protein expression. For reproducibility, all the experiments were performed with at least three biological replicates on all the cell lines; *p*-values that were equal to or less than 0.05 were considered to have statistical significance.

## 3. Results

### 3.1. ROR1 Regulates DNMT3A and DNMT3B Expression and Activity in TNBC Cell Lines

**Figure 1:**
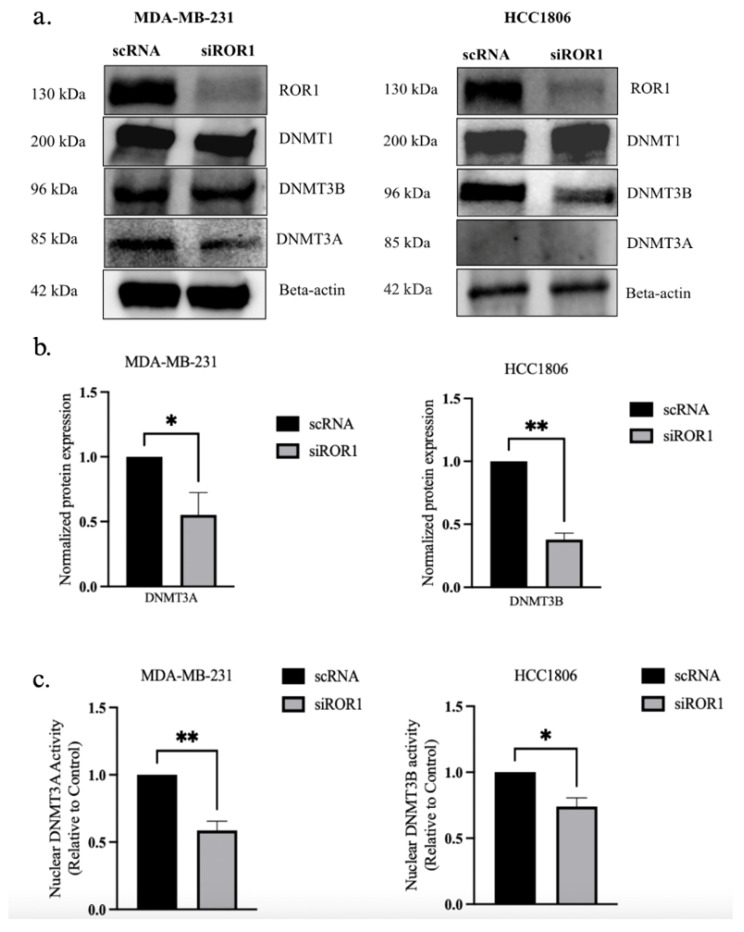
ROR1 knockdown decreases DNMT3A and DNMT3B expression and activity in TNBC cells. (**a**) Western blot analysis comparing protein levels of DNMTs in control and siROR1 in MDA-MB-231 and HCC1806 cells. (**b**) Quantification of Western blot analysis. In MDA-MB-231 cells, ROR1 knockdown resulted in a 0.5-fold change in DNMT3A protein levels. In HCC1806 cells, ROR1 knockdown resulted in a 0.4-fold change in DNMT3B protein levels. (**c**) The bar graph revealed that knocking down ROR1 expression reduces the nuclear activity of DNMT3A and DNMT3B in TNBC cells (** *p* < 0.01 * *p* < 0.05, n = 3). Statistical significance was determined through paired Student’s *t*-tests.

To determine if ROR1 regulates DNMTs in TNBC, we evaluated the protein expression of DNMTs in two TNBC subtypes, HCC1806 and MDA-MB-231, following ROR1 knockdown (siROR1). The controls included the same TNBC cell lines that were treated with scrambled RNA (scRNA). The Western blot analysis showed no significant change in DNMT1 expression upon ROR1 knockdown (*p* > 0.05, Figure 1a). However, we saw a significant reduction in DNMT3A and DNMT3B expression in the siROR1-treated cells compared to the scRNA samples (*p* < 0.0235 and *p* < 0.0174, Figure 1a–c). Notably, DNMT3A was not detected in HCC1806 cells but was expressed in MDA-MB-231 cells, suggesting subtype-specific DNMT expression. Interestingly, DNMT3B protein levels were significantly reduced when ROR1 was knocked down in the HCC1806 cells, whereas no significant changes were observed in the MDA-MB-231 cells, indicating potential differential regulatory mechanisms across TNBC subtypes. To further evaluate the functional impact of ROR1 knockdown, we performed DNMT activity assays. We observed a significant decrease in the amount of DNMT3A (*p* < 0.0091) and DNMT3B-mediated (*p* < 0.0207) methylation of a DNA substrate in MDA-MB-231 and HCC1806 siROR1-treated cells compared to scRNA controls (Figure 1c). These results indicated that ROR1 positively regulates the enzymatic activity of DNMT3A and DNMT3B in TNBC cells. Given that DNMT3A and DNMT3B play critical roles in DNA methylation, we assessed whether ROR1 knockdown influences CpG methylation patterns in TNBC. Hence, we performed a bisulfite sequencing analysis in MDA-MB-231 cells to determine whether ROR1 expression correlated with changes in DNA methylation at CpG sites.

### 3.2. ROR1 Knockdown Reduces the DNA Methylation Status of the CpG Regions Throughout the MDA-MB-231 Genome

**Figure 2:**
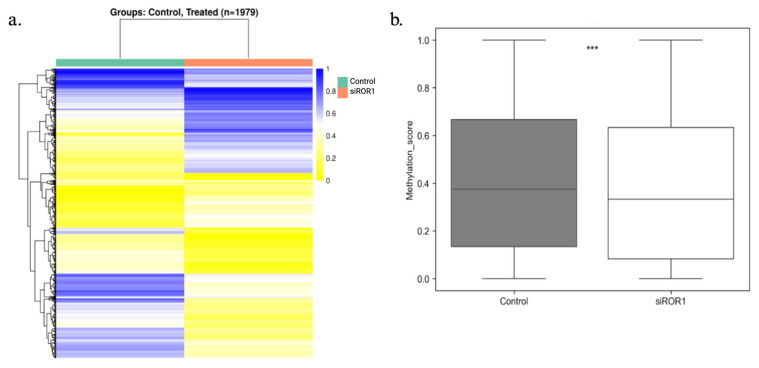
ROR1 is a regulator of DNA hypermethylation in MDA-MB-231 cells. Reduced representation bisulfite sequencing results comparing the gene methylation status for naive MDA-MB-231 cells (control) and MDA-MB-231 cells treated with MDA-MB-231 cells knocked down for ROR1. (**a**) Heatmap analysis showing the differentially methylated regions (DMR) between control and treated samples. The percent methylation ranges from 1 blue to 0 yellow. (**b**) Box plot showing the overall methylation status of the CpG regions within the MDA-MB-231 genomes in control vs. siROR1 samples. (Wilcoxon test, *** *p* = 8.532 × 10^−16^).

RRBS was performed to investigate how decreasing ROR1 expression would affect the methylation of CpG regions within TNBC cells. The analysis identified 1979 DMRs between MDA-MB-231 cells with high ROR1 expression to the MDA-MB-231 cells with low ROR1 protein expression following siRNA knockdown (Figure 2a). The methylation levels of the CpG regions were represented in blue if they were fully methylated, but the unmethylated regions were represented in yellow. The ROR1 knockdown resulted in a significant decrease in genome-wide CpG methylation compared to our control samples (*** *p* = 8.532 × 10^−16^, Figure 2b). These findings support the hypothesis that ROR1 modulates methylation patterns in TNBC cells. The functional enrichment analysis of the differentially methylated regions revealed that the affected genes were mainly involved in biological processes such as nervous system development, cell adhesion, signaling, differentiation, proliferation, and cell morphogenesis. Given that these processes are critical for embryogenesis and that ROR1 is a key regulator of embryonic development [19] these findings align with the known functional role of ROR1 in developmental pathways.

### 3.3. ROR1 Knockdown Hinders the Physical Binding of DNMT3A and DNMT3B to the Coding Region of the CREB3L1 Gene in TNBC Cells

**Figure 3:**
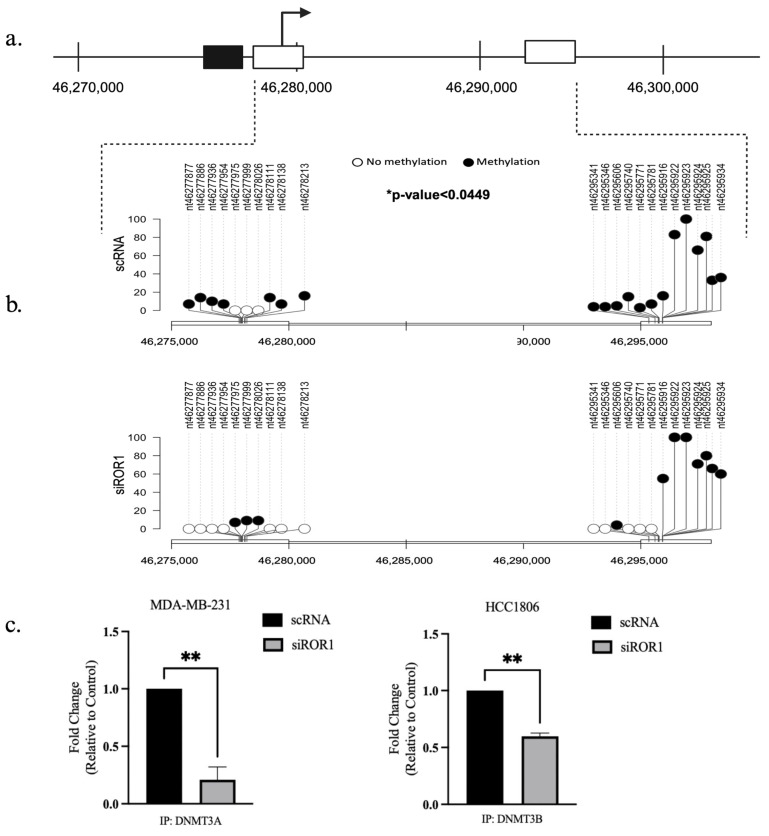
ROR1 knockdown reduced methylation of the CpG sites within the CREB3L1 gene. (**a**) Schematic of the CREB3L1 gene in MDA-MB-231 cells, chromosome location 11p11.2 (RefSeq ID: NM_052854.4). The black box represents the promoter region of the gene and its location within the gene. The white boxes represent the CpG islands in the gene and their proximal distance to the promoter region. (**b**) Lollipop plot illustrating the specific locations of 21 differentially methylated CpG sites in the CREB3L1 gene in the scRNA vs. siROR1 samples. The black dots represent methylated cytosines, and the white dots represent unmethylated cytosines. The Y-axis indicates methylation percentage (* *p* < 0.05). (**c**) ChIP assay demonstrates that ROR1 expression regulates the ability of DNMT3A and DNMT3B to bind to the CREB3L1-coding region in TNBC cells. Cell lines with ROR1 expression knocked down showed a 0.25-fold decrease in DNMT3A and a 0.6-fold change in DNMT3B bound to the CREB3L1-coding region compared to TNBC cells treated with scrambled RNA (** *p* < 0.01, n = 3). Statistical significance was determined using a paired *t*-test.

Through RRBS we observed that *CREB3L1*, a known tumor suppressor in TNBC, was one of the genes that had significant differential methylation patterns when ROR1 was knocked down in MDA-MB-231 cells. Using the UCSC genome browser, we were able to identify 2 CpG regions located less than 2 kb upstream of the CREB3L1 promoter region (Figure 3a). The analysis of the *CREB3L1* gene in the MDA-MB-231 cell line demonstrated that at the location 11p11.2, the CpG-rich regions between the promoter position 46277451 (start) to 46277794 (end) were hypermethylated. Between the control MDA-MB-231 samples and the ROR1 knockdown MDA-MB-231 samples, there were 21 differentially methylated CpG sites within the 2 regions. Knocking down ROR1 resulted in a significant decrease in *CREB3L1* CpG methylation (*p* < 0.049, Figure 3b). The differentially methylated CpG sites were located within the coding region of the *CREB3L1* gene. To assess if knocking down ROR1 expression affects the ability of DNMT3A and DNMT3B to bind to the *CREB3L1*-coding region, a ChIP assay was performed. The results show that knocking down ROR1 decreased the amount of DNMT3A (*p* < 0.0065) and DNMT3B (*p* < 0.0018) bound to the *CREB3L1*-coding region in the MDA-MB-231 and HCC1806 cells, respectively (Figure 3c). Taken together, these results support our hypothesis that knocking down ROR1 expression reduces the physical interaction between the methyltransferases (DNMT3A and DNMT3B) and the *CREB3L1*-coding region, leading to a decrease in the CpG methylation status of the *CREB3L1* in TNBC cells.

### 3.4. ROR1 Knockdown Reverses CREB3L1 Silencing in TNBC Cells

**Figure 4:**
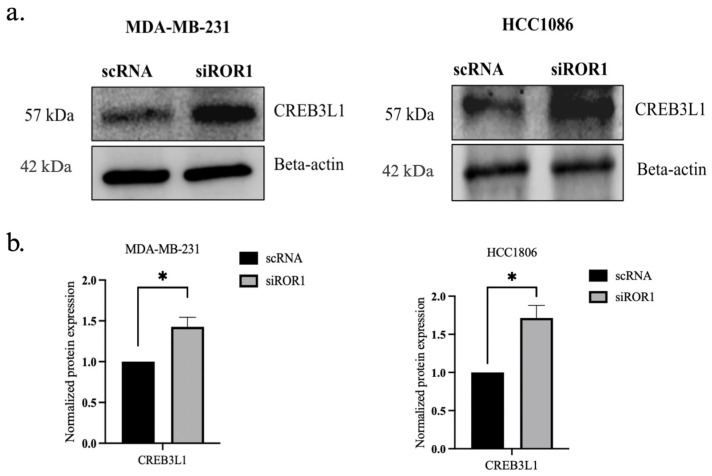

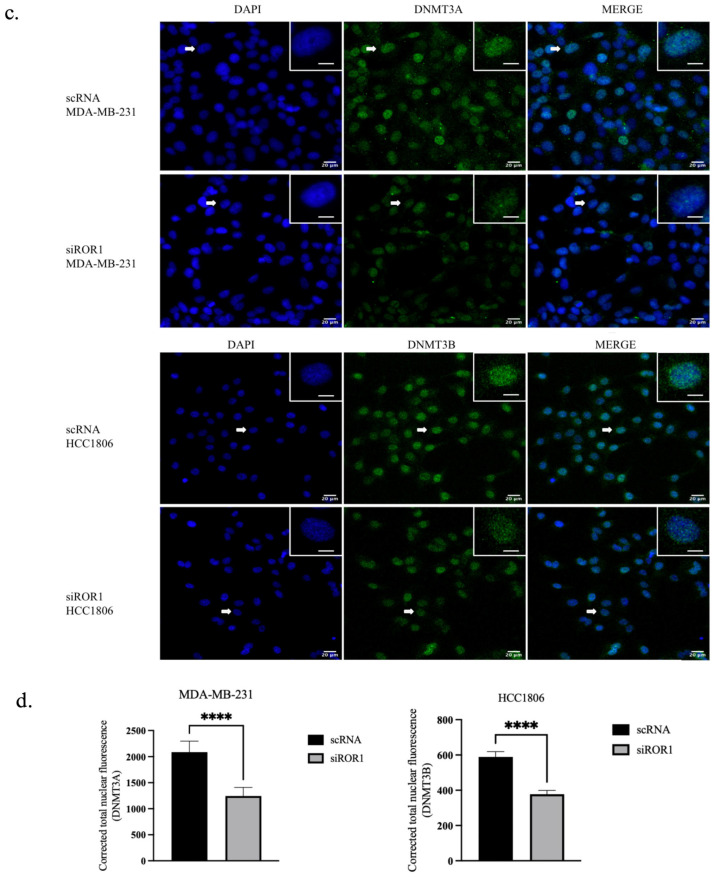

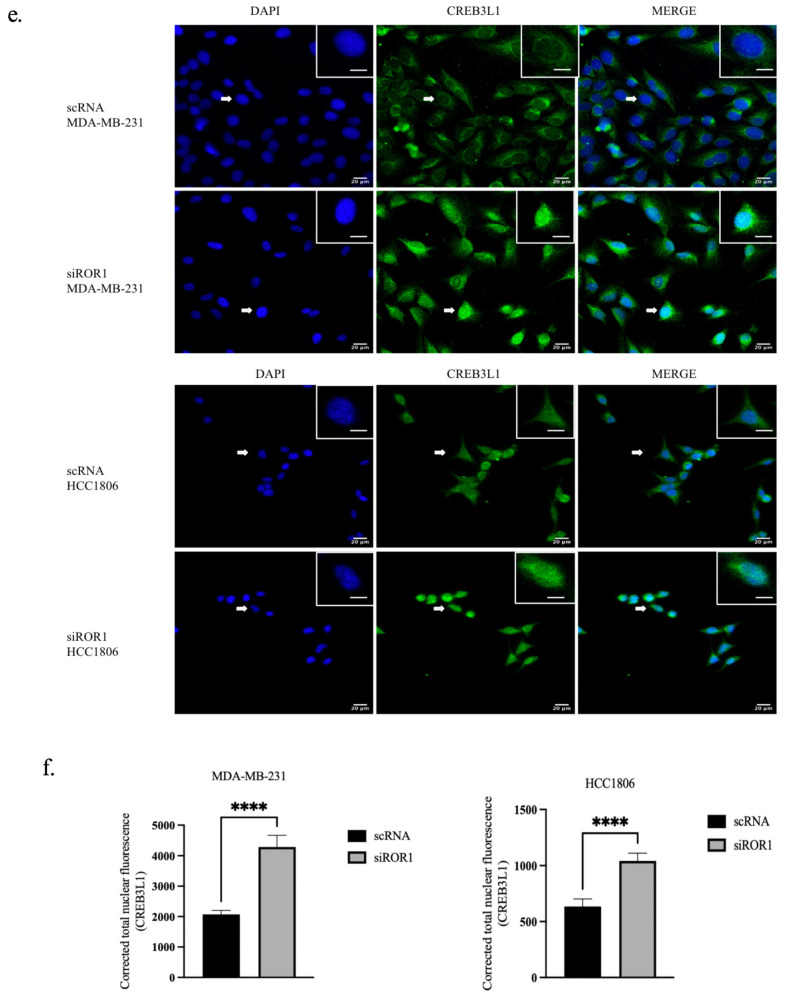
CREB3L1 nuclear expression increased and nuclear DNMT3A/DNMT3B expression decreased following ROR1 knockdown. (**a**) Western blot analysis investigating the effect of ROR1 knockdown by siRNA on CREB3L1. (**b**) Quantification of Western blot analysis. ROR1 knockdown resulted in a 1.4-fold change in CREB3L1 expression in MDA-MB-231 cells and a 1.7-fold change in CREB3L1 protein expression in HCC1806 cells (* *p* < 0.05, n = 3). (**c**–**f**) Immunofluorescence analysis and quantification investigating how ROR1 expression affects the nuclear expression of (**c**,**d**) DNMT3A, DNMT3B, and (**e**,**f**) CREB3L1 (**** *p* < 0.0001, n = 30). ROR1 knockdown resulted in a 0.6-fold change in DNMT3A nuclear expression in MDA-MB-231 cells and a 0.6-fold change in DNMT3B nuclear expression in HCC1806 cells. ROR1 knockdown resulted in a 2-fold change in nuclear CREB3L1 expression within MDA-MB-231 cells and a 1.6-fold change in CREB3L1 nuclear expression in HCC1806 cells. The cells that are marked with a white arrow appear in the top right corner of each image. Scale bars represent 20 uM. Statistical significance was determined through paired Student’s *t*-tests.

In MDA-MB-231 and HCC1806 TNBC cells, ROR1 knockdown resulted in an increase of CREB3L1 expression (*p* < 0.0235 and *p* < 0.0174) compared to the control TNBC samples (Figure 4a,b). To further confirm the reactivation of CREB3L1, after knocking down ROR1, we conducted an immunofluorescence analysis to specifically quantify the nuclear expression of CREB3L1, DNMT3A, and DNMT3B in our TNBC cell samples. Given that DNMT3A and DNMT3B are DNA methyltransferases responsible for the methylation of DNA, these enzymes are localized to the nucleus. Upon activation, the CREB3L1 protein translocates to the nucleus, where it can transcribe its target genes [36]. Here, we show that ROR1 knockdown decreased DNMT3A/DNMT3B nuclear protein expression in the MDA-MB-231 and HCC1806 cell lines (Figure 4c,d). The immunofluorescence analysis results corroborate our results from Figure 1, demonstrating that the nuclear localization of CREB3L1 protein was enhanced in the MDA-MB-231 and HCC1806 cell lines upon ROR1 knockdown (Figure 4e,f). These results suggest that decreasing ROR1 in TNBC cells can reverse the epigenetic silencing of *CREB3L1*, leading to an increase in its expression.

### 3.5. ROR1 Activates STAT3 Phosphorylation in TNBC Cells

**Figure 5:**
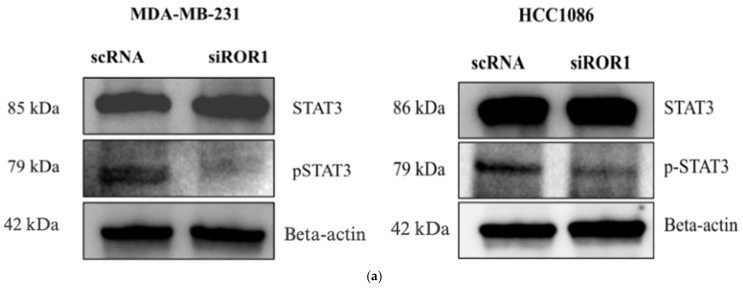

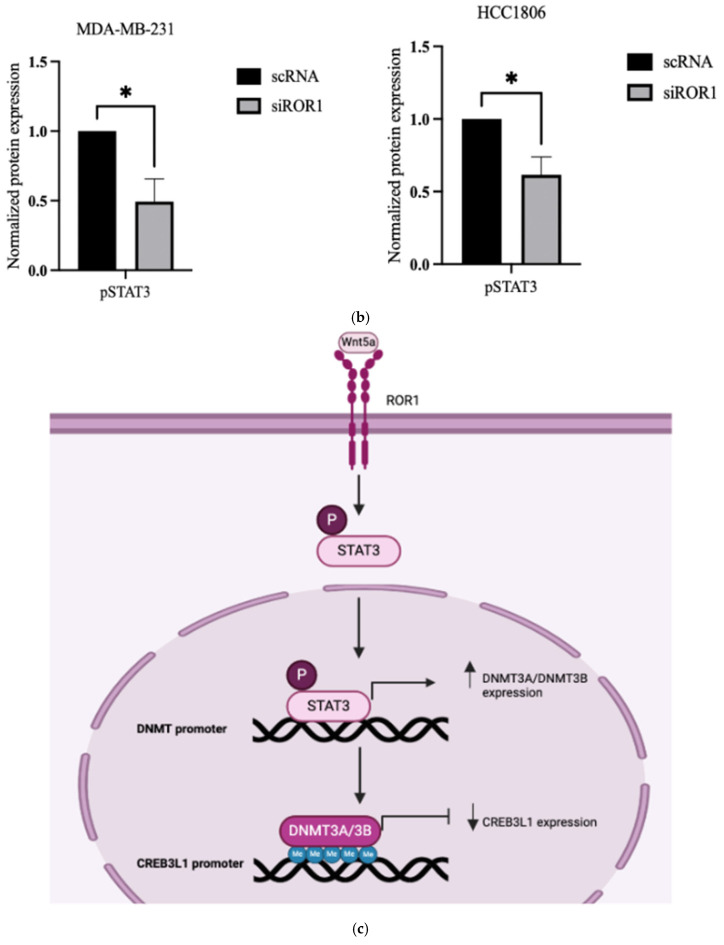
ROR1 activated STAT3 phosphorylation in TNBC cells. (**a**) Western blot analysis comparing the protein expression of STAT3 and pSTAT3 in MDA-MB-231 and HCC 1806 cells with high ROR1 expression (scRNA-control) compared to the same cell lines with ROR1 knocked down (siROR1). (**b**) Quantification of Western blot analysis shows that ROR1 knockdown decreases phosphorylated STAT3 (* *p* < 0.05, n = 3). Statistical significance was determined through paired Student’s *t*-tests. (**c**) Hypothetical figure illustrating the potential signaling pathway that ROR1 is activating to silence CREB3L1 expression in TNBC.

ROR1 is a transmembrane receptor found on the surface of cells, whereas DNMT3A and DNMT3B are nuclear proteins responsible for DNA methylation [19]. We next sought to investigate the mechanistic link between ROR1 signaling and downstream activation of DNMT activity. The STAT3 (signal transducer and activator of transcription 3) protein has been implicated in the CpG methylation of tumor suppressor genes in liver, breast, and hematopoietic cancers by promoting the expression, activation, and stability of DNMTs [39,40,41,42,43,44]. Specifically, STAT3 phosphorylation has been found to increase DNMT1, DNMT3A, and DNMT3B expression in cancer cells [39,42,43]. ROR1 is known to activate signaling pathways through phosphorylation in cancers such as chronic lymphocytic leukemia (CLL) and ovarian cancer, including downstream JAK/STAT effector proteins [45,46]. However, the role of ROR1 in regulating STAT3 in TNBC is yet to be established. We observed a reduction in the levels of pSTAT3 in MDA-MB-231 cells (*p* < 0.0145) and HCC1806 cells (*p* < 0.0310) following ROR1 knockdown (Figure 5a,b). Based on this, we hypothesize that ROR1 promotes STAT3 phosphorylation, which subsequently increases DNMT3A/DNMT3B expression and activity, resulting in the epigenetic silencing of *CREB3L1* expression (Figure 5c).

### 3.6. STAT3 Phosphorylation Has an Inverse Effect on CREB3L1 Expression in TNBC Cells

**Figure 6:**
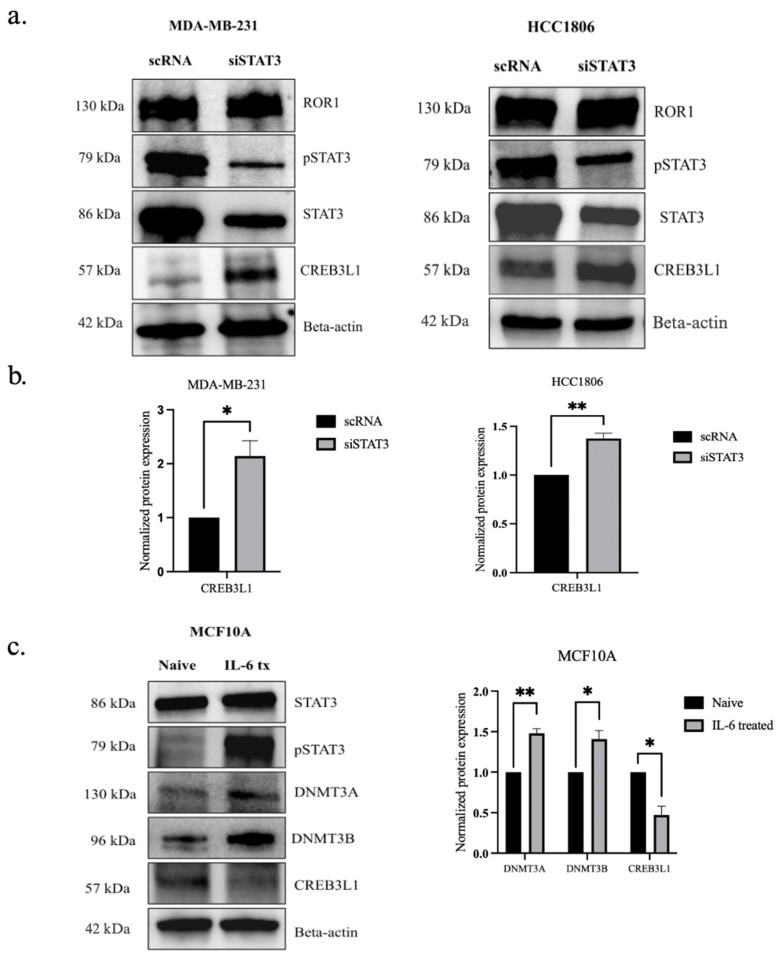
STAT3 phosphorylation had an inverse effect on CREB3L1 protein expression in TNBC. (**a**) Western blot analysis comparing the expression of STAT3, pSTAT3, and CREB3L1 in TNBC cells treated with scRNA to the same cell lines with ROR1 expression knocked down (siROR1). (**b**) Quantification of Western blot analysis (n = 3). Statistical significance was determined through paired Student’s *t*-test (* *p* < 0.05, ** *p* < 0.01). (**c**) Western blot analysis comparing the protein expression of ROR1, STAT3, pSTAT3, DNMT3A, DNMT3B, and CREB3L1 in naive MCF10A cells compared to the same cell line that has been transfected with IL-6 recombinant protein (* *p* < 0.05, ** *p* < 0.01, n = 3).

To determine if pSTAT3 activation is essential for the epigenetic silencing of CREB3L1 in TNBC, we first examined whether we knocked down STAT3 in the TNBC cells by transfecting them with siSTAT3. The STAT3 silencing resulted in increased CREB3L1 expression in both MDA-MB-231 (*p* < 0.0198) and HCC1806 cells (*p* < 0.0069), supporting the hypothesis that STAT3 plays a key role in ROR1-dependent CREB3L1 silencing (Figure 6a,b).

To further confirm that ROR1 mediates the CREB3L1 silencing via STAT3, we forced pSTAT3 activation in non-malignant MCF10A cells, which express low levels of ROR1. The results show that pSTAT3 activation in MCF10A cells increased DNMT3A/DNMT3B expression (*p* < 0.0047 and *p* < 0.0212), accompanied by the depletion of CREB3L1 protein levels (*p* < 0.0139), even though ROR1 expression remained low (Figure 6c). We observed a molecular weight of 130 kDa for DNMT3A in the MCF10A cell line, which differs from what was observed in the MDA-MB-231 cell line (Figure 1a). In the MDA-MB-231 cell line, we observed DNMT3A protein at 85 kDa. DNMT3A has two isoforms, DNMT3A1 and DNMT3A2, with molecular weights of approximately 130 kDa and 85 kDa, respectively [47].

## 4. Discussion

The epigenetic dysregulation of oncogenes and tumor suppressor genes, which is frequently observed in TNBC, drives disease heterogeneity and therapeutic response in patients. However, there is currently a lack of comprehensive understanding of the epigenetic regulation of tumor suppressor genes in TNBC. The oncogene-mediated epigenetic silencing of tumor suppressor genes is an established mechanism by which the tumor suppressor function is impaired [9]. Our study has identified ROR1, a known oncogene, to be an upstream epigenetic regulator of a tumor suppressor, CREB3L1, in MDA-MB-231 and HCC1806 TNBC cell lines.

To examine the impact of ROR1 on the differential methylation of regulatory elements in TNBC, we conducted a reduced representation bisulfite sequencing (RRBS) on MDA-MB-231 cells, which have high ROR1 expression, and compared them to the same cell line with ROR1 knocked down. We screened the hits from the RRBS dataset for genes exhibiting reduced CpG methylation in the promoter, non-coding, and coding regions in the MDA-MB-231 ROR1 knockdown cells (https://zenodo.org/records/15236296, accessed on 17 April 2025). We further refined our selection to the tumor suppressor genes that are epigenetically silenced and not mutated in TNBC and showed a differential methylation pattern when ROR1 was knocked down. Our findings indicate that the methylation of sites near the promoter region of the *CREB3L1* tumor suppressor gene decreases when ROR1 is knocked down. This was validated by monitoring the CREB3L1 protein levels in TNBC subtypes using immunofluorescence and Western blotting. Furthermore, the ChIP assays confirmed the decrease in the physical interaction between DNMTs and the CREB3L1-coding region following ROR1 knockdown in TNBC cells.

Additionally, our study identifies STAT3 to be a key player that is influenced by ROR1 in TNBC cells. ROR1 regulates STAT3 phosphorylation in TNBC cells, a key step in repressing *CREB3L1* gene expression by promoting DNMT upregulation. Nuclear phosphorylated STAT3 is known to regulate the DNMTs in hematopoietic cancer and colon cancer by binding to DNMT promoters to activate their expression [41,48]. Our findings establish ROR1 as a key epigenetic modulator of CREB3L1 through a STAT3-dependent mechanism, providing novel insights into oncogene-mediated epigenetic regulation in TNBC.

Interestingly, a previous study classified *CREB3L1* as an oncogene, promoting TNBC metastasis through the regulation of PERK signaling and EMT [49]. However, several other studies have demonstrated *CREB3L1* as a tumor suppressor in TNBC patients [34,35,50]. CREB3L1 is activated in response to endoplasmic reticulum (ER) stress, which is commonly seen in tumors due to hypoxia and nutrient deprivation [30]. Moreover, CREB3L1 expression was also demonstrated to be imperative for the efficacy of doxorubicin and paclitaxel in blocking the proliferation of cancer cells [34,35]. Hypermethylation of *CREB3L1* at its promoter, as well as within coding and non-coding regions, has been established in TNBC as well as human bladder cancer [33,51]. An overlap in the hypermethylated sites has been identified between TNBC and bladder cancer patients, spanning from +197 bases to +554 bases relative to the transcription start site (TSS) of *CREB3L1* [33,51]. However, there is no evidence in the literature indicating a direct role of ROR1 in the epigenetic silencing of tumor suppressors, such as *CREB3L1*, despite its association with poor prognosis in TNBC and bladder cancer [52,53,54,55,56]. The data from this study establish a mechanism by which ROR1 regulates the methylation of key tumor suppressors in TNBC. These results introduce a novel paradigm in the epigenetic regulation mediated by oncogenes. Since ROR1 is upregulated in several malignancies [25], we propose further investigation into its role in the epigenetic silencing of tumor suppressors across various cancer types.

Previous studies have shown that the subcellular localization of CREB3L1 shifts from the cytoplasm to the nucleus, a process consistent with its activation during cellular stress [57]. In high-grade TNBC tumors, CREB3L1 protein is significantly reduced and predominantly localized in the cytoplasm, whereas, in low-grade TNBC tumors, CREB3L1 is more frequently localized in the nucleus, indicating its active state [57]. A similar pattern is observed in bladder cancer progression, where CREB3L1 is localized in the nucleus of less aggressive tumors but remains in the cytoplasm of late-stage tumors. In the ROR1 knockdown TNBC cell samples, CREB3L1 localization was predominantly nuclear rather than cytoplasmic. This may suggest that ROR1 plays a role in the epigenetic silencing of *CREB3L1* in TNBC and potentially in bladder cancer as well. Furthermore, the expression of DNMT3A and DNMT3B was shown to be diminished in the nucleus of ROR1 knockdown TNBC cells, indicating that ROR1 may regulate DNMT levels to suppress CREB3L1.

Our results show that ROR1 epigenetically silences *CREB3L1* expression via the STAT3-DNMT regulatory axis. ROR1 knockdown resulted in increased levels of pSTAT3 in TNBC cells. Previous studies have shown that pSTAT3 activation enhances DNMT3B expression in hepatocellular carcinoma and colon cancer [43,48]. Additionally, pSTAT3 has been shown to upregulate DNMT1 expression in various cancers, including hepatocellular carcinoma, lymphoma, colon, and breast cancer [43,44,48,56]. In TNBC xenograft models, pSTAT3 activation was found to increase DNMT1 expression, resulting in the downregulation of tumor suppressor genes [57]. However, the relationship between pSTAT3 and DNMT3A/DNMT3B has yet to be established in TNBC. To confirm that the epigenetic regulation is mediated by ROR1-STAT3 pathways, we inhibited STAT3 expressions via siRNA transfection, which resulted in an increased CREB3L1 expression in MDA-MB-231 and HCC1806 cells. To further investigate the role of pSTAT3 in normal breast cells, we induced pSTAT3 activation in a non-tumorigenic MCF10A cell line by transfecting the cells with recombinant IL-6. This led to an increase in DNMT3A/DNMT3B levels and a corresponding decline in CREB3L1 expression. These data proved that the ROR1-mediated epigenetic silencing of CREB3L1 is dependent on pSTAT3 activation, providing a novel insight into the regulatory mechanisms governing tumor suppressor gene silencing in TNBC.

DNA methylation is a reversible process that has led to the clinical evaluation of several DNMT inhibitors in cancer therapy (NCT01349959, NCT00978250, NCT03295552, and NCT00748553). However, the trials for these hypomethylating agents have not been clinical success, primarily due to their lack of specificity in targeting DNMTs that are selectively active in cancer cells [58]. Given this limitation, a combination therapy involving ROR1 inhibitors and the DNMT inhibitor decitabine may enhance therapeutic efficacy [18]. There are several tumor suppressors other than *CREB3L1* that could be silenced by ROR1, suggesting that targeting ROR1 and its downstream DNMT-mediated epigenetic modifications could provide a novel therapeutic strategy for TNBC. DNA methyltransferases, such as DNMT1, DNMT3A, and DNMT3B, have been implicated in the progression of multiple malignancies including breast cancer and acute myeloid leukemia [18,59]. Furthermore, as *CREB3L1* silencing has been observed in lung squamous cell carcinoma, colon, adrenal gland, rectum, cervix, and liver cancers, the findings from this study may have broader implications beyond TNBC [31].

Despite the recent advances in TNBC treatment options, with the FDA approval of targeted and immunotherapeutic drugs, patients continue to experience drug resistance and off-target cytotoxicity [5,60]. The heterogeneity of TNBC presents a significant challenge in treatment [61]. In this study, we utilized two cell lines, MDA-MB-231 and HCC1806, that represent the mesenchymal stem-like and basal-like 2 subtypes, respectively [37]. Both subtypes have high ROR1 expression, which we had shown correlates with elevated DNMT levels and low CREB3L1 expression. We found that the primary DNMT controlling *CREB3L1* was specific to each TNBC cell line. However, despite the subtype-specific differences, we have demonstrated that ROR1 silencing effectively suppresses DNMT expression and activity, thereby restoring *CREB3L1* expression in TNBC cells. This suggests that targeting ROR1 may be a potential strategy to reverse the epigenetic silencing of tumor suppressor genes in TNBC.

## 5. Conclusions

In conclusion, this study provides evidence for a novel unrecognized role of ROR1 as an epigenetic regulator of tumor suppressor genes in TNBC. We have established that ROR1 promotes *CREB3L1* gene methylation through the activation of pSTAT3, leading to its epigenetic silencing. The epigenetic silencing of CREB3L1 was reversed upon ROR1 knockdown and by the inhibition of STAT3 activation, indicating a direct regulatory mechanism. Our data offer novel therapeutic opportunities aimed at targeting ROR1 in malignancies characterized by low CREB3L1 expression. The findings introduce a new strategy for epigenetic reactivation of tumor suppressor genes in TNBC.

## Data Availability

All the data produced and analyzed for this study have been included in the manuscript and the Supplementary File S1.

## Supplementary Materials

The following supporting information can be downloaded at: https://www.mdpi.com/article/10.3390/biom15050734/s1, Supplementary File S1: The original western blot images.

## Abbreviations

CREB3L1	cAMP responsive element binding protein 3-like-1
DNMT	DNA methyltransferase
DMR	Differentially methylated region
ROR1	Receptor tyrosine kinase-like orphan receptor 1
RRBS	Reduced representation bisulfite sequencing
STAT3	Signal transducer and activator of transcription 3 protein
TNBC	Triple-negative breast cancer
