# Supplementary material for: Uncovering a Novel Role of ROR1 in the Epigenetic Regulation of Tumor Suppressor Gene CREB3L1 in Triple-Negative Breast Cancer Cells
Source: Biomolecules. 2025 May 16;15(5):734. doi: 10.3390/biom15050734 (PMC12109183; doi:10.3390/biom15050734)

Raw western blot data

1. All lanes used in the manuscript and used for densitometry analysis are labeled. Unlabeled lanes were not used for analysis.
2. All proteins used in the manuscript and for analysis are labeled “ctl” or “si”. Lanes labeled “X” were not utilized.
3. Protein lysates were obtained from MDA-MB-231 and HCC1806 cell lines.
4. Lanes labeled “si” are proteins from cells lines that were treated with siROR1 Lanes that are labeled “Ctl” are the control samples treated with scrambled RNA (scRNA).
5. All densitometry analysis was normalized to a housekeeping gene, beta-actin or GAPDH
6. Brightness and contrast of blots may have been adjusted to make the protein bands more visible in the manuscript.

MDA-MB-231 cells_ROR1_replicates1&2

Figure 1

- Representative image in Figure 1a of the manuscript

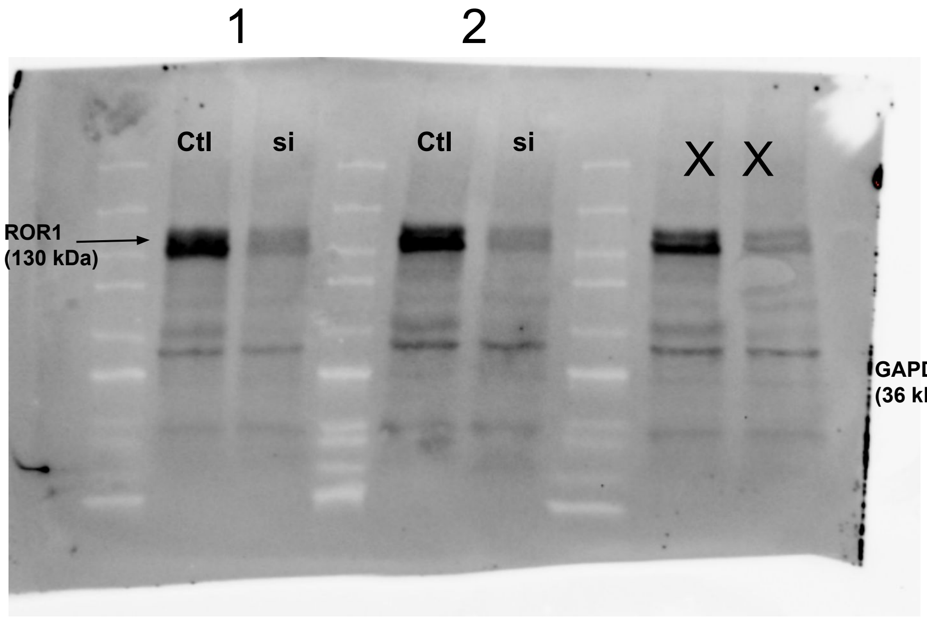

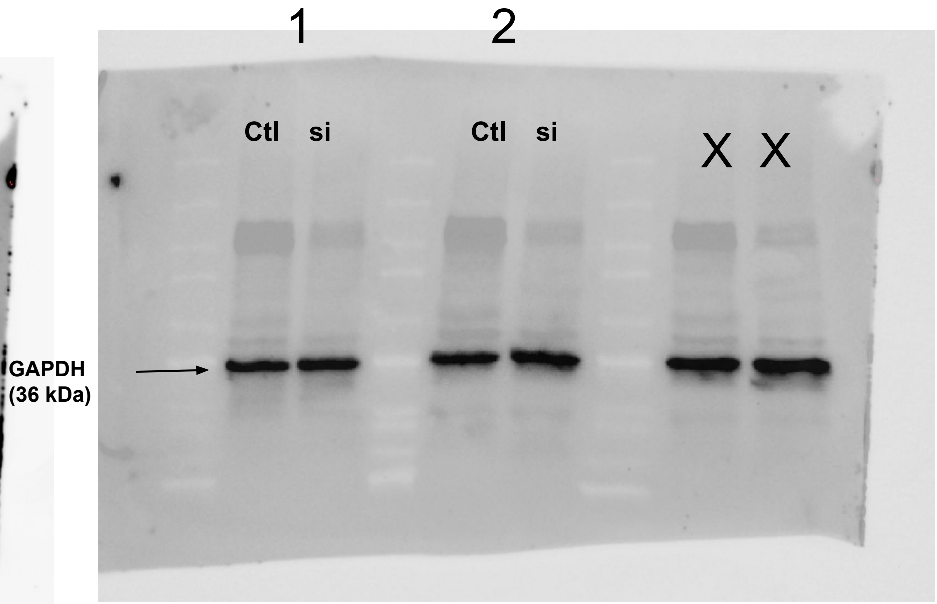

MDA-MB-231 cells_ROR1_replicate3

Figure 1

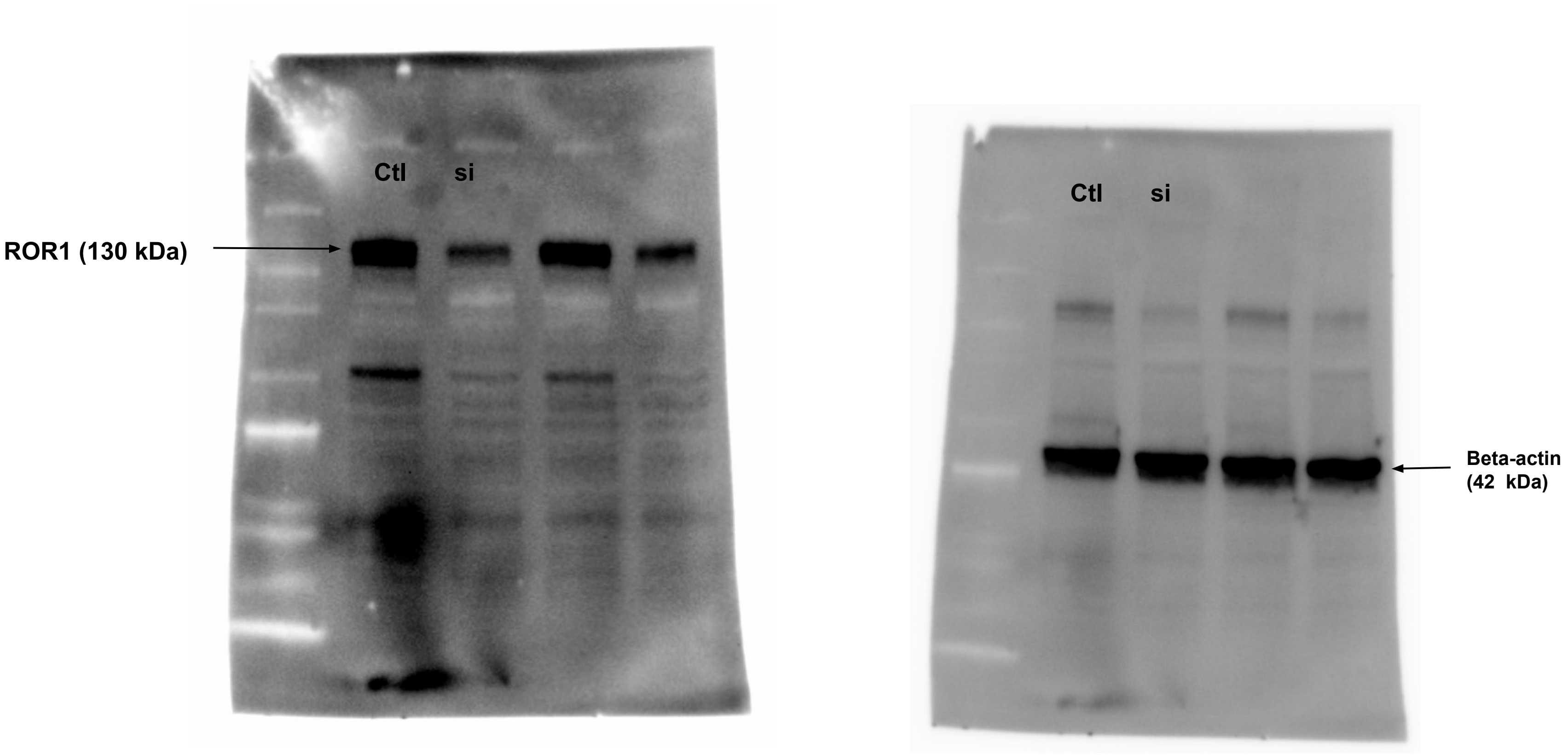

HCC1806 cells_ROR1_replicate1

Figure 1

- Representative image in Figure 1a of the manuscript

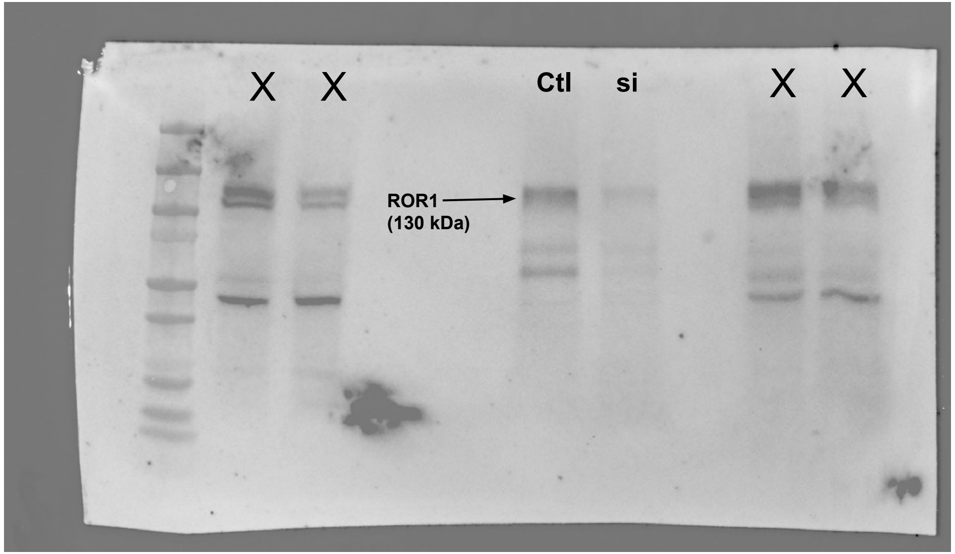

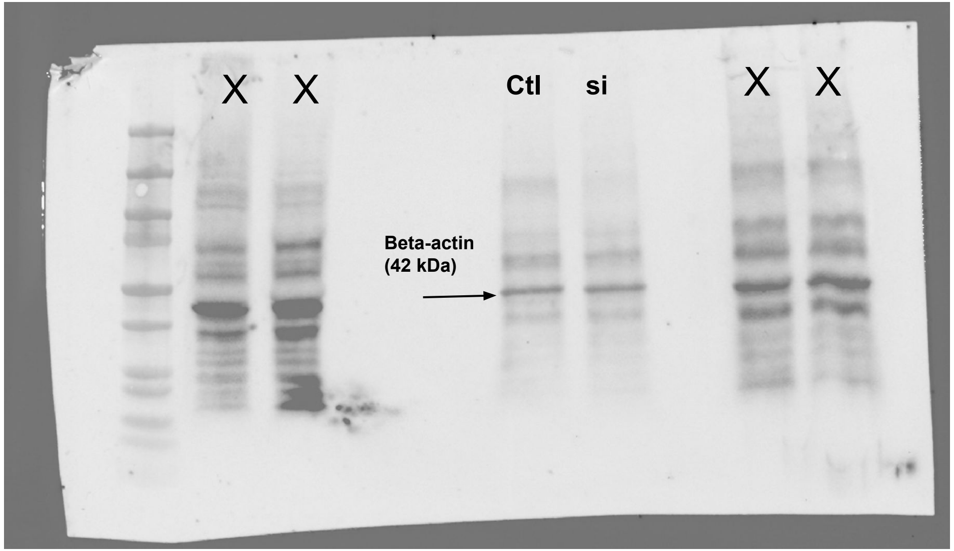

HCC1806 cells_ROR1_replicate2

Figure 1

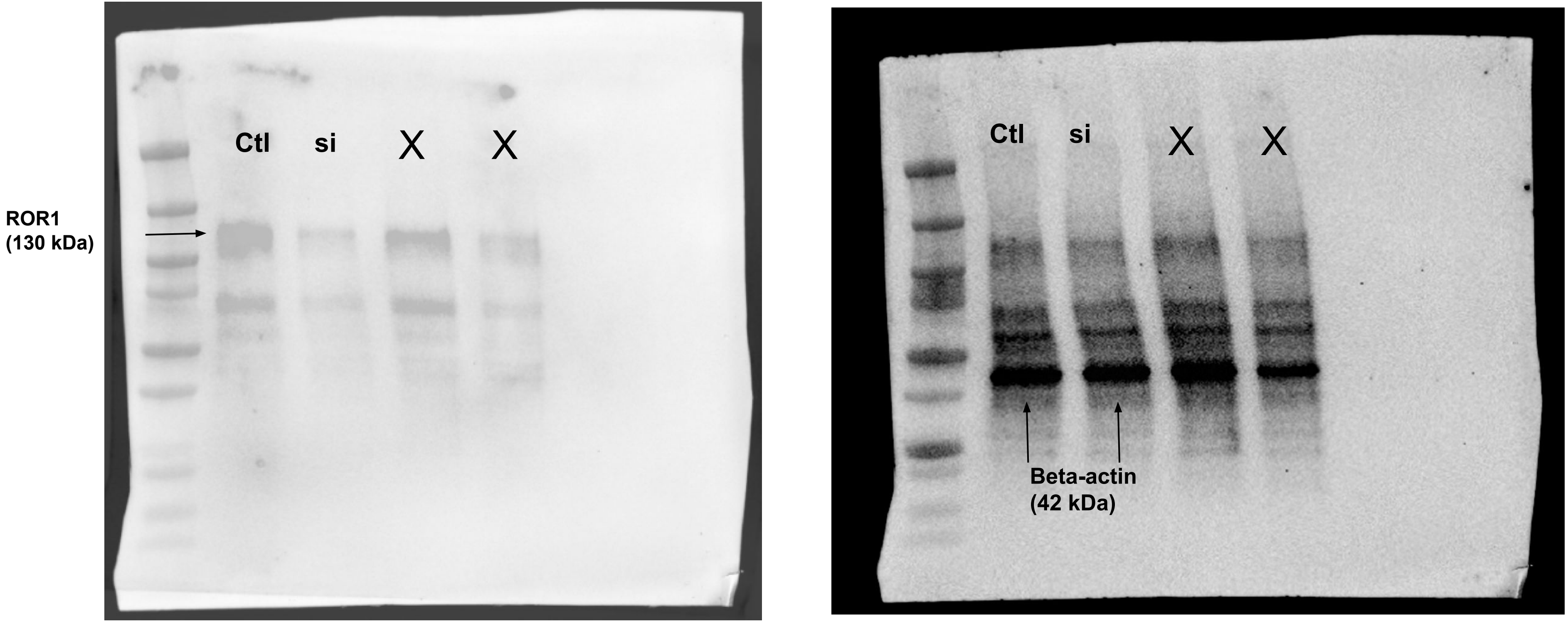

HCC1806 cells_ROR1_replicate3

Figure 1

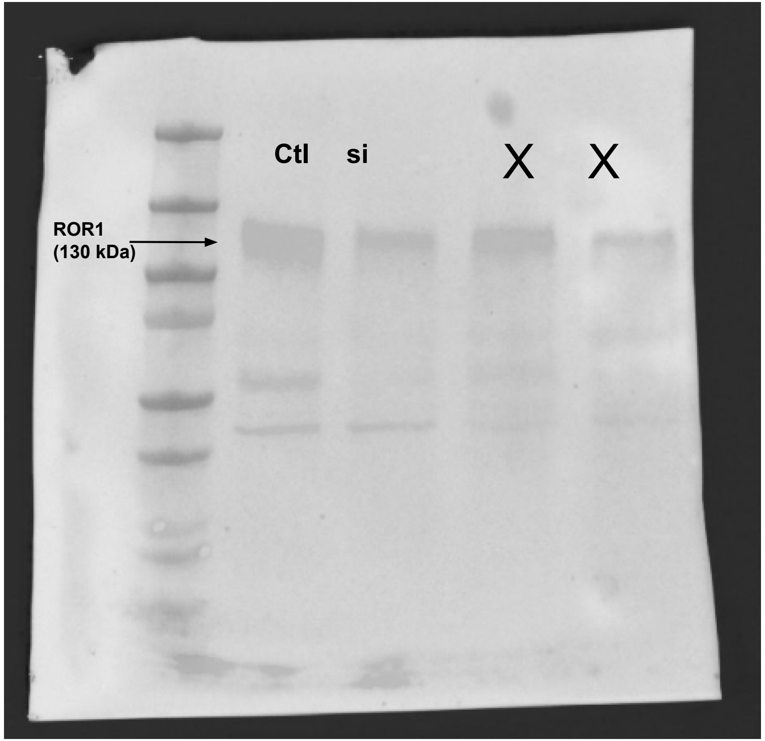

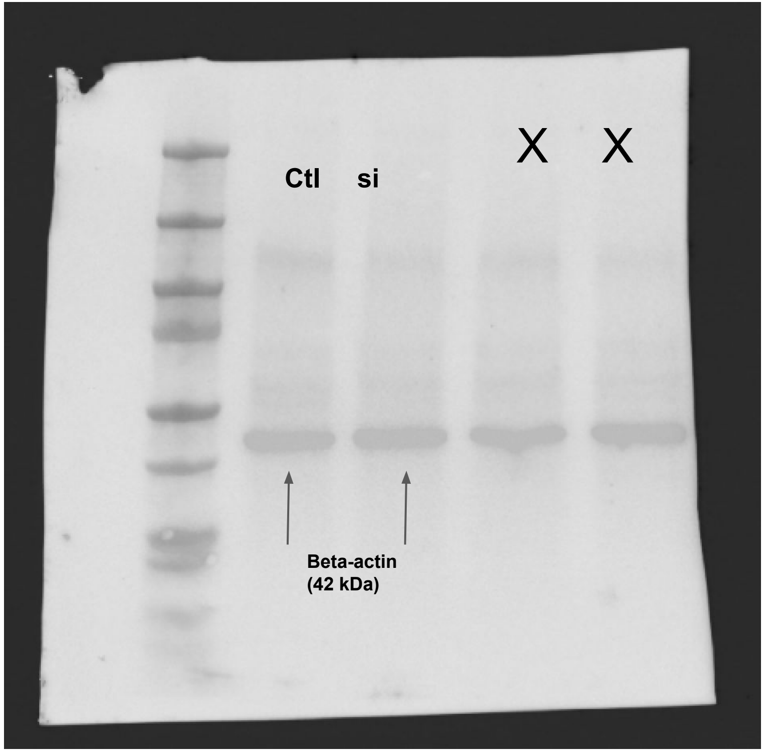

MDA-MB-231 cells_CREB3L1_replicate1

- Representative image in Figure 4a

Figure 4

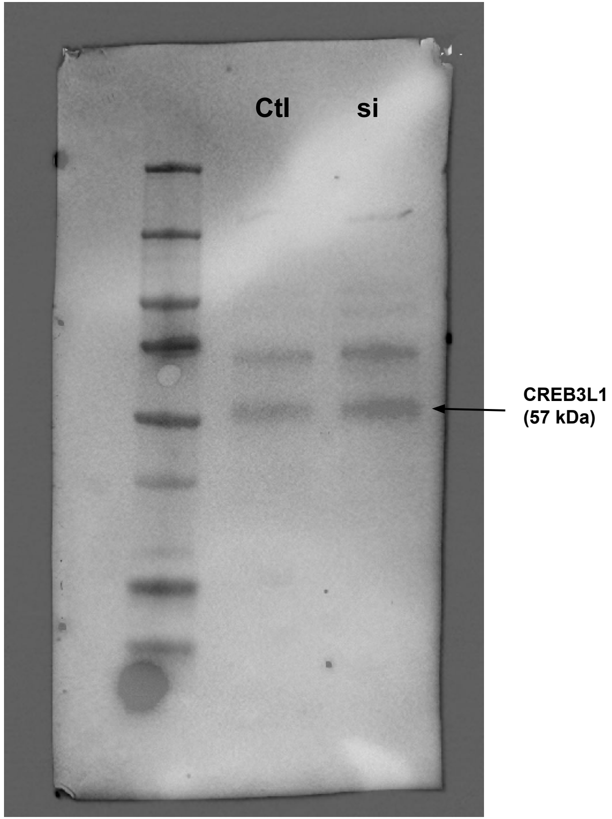

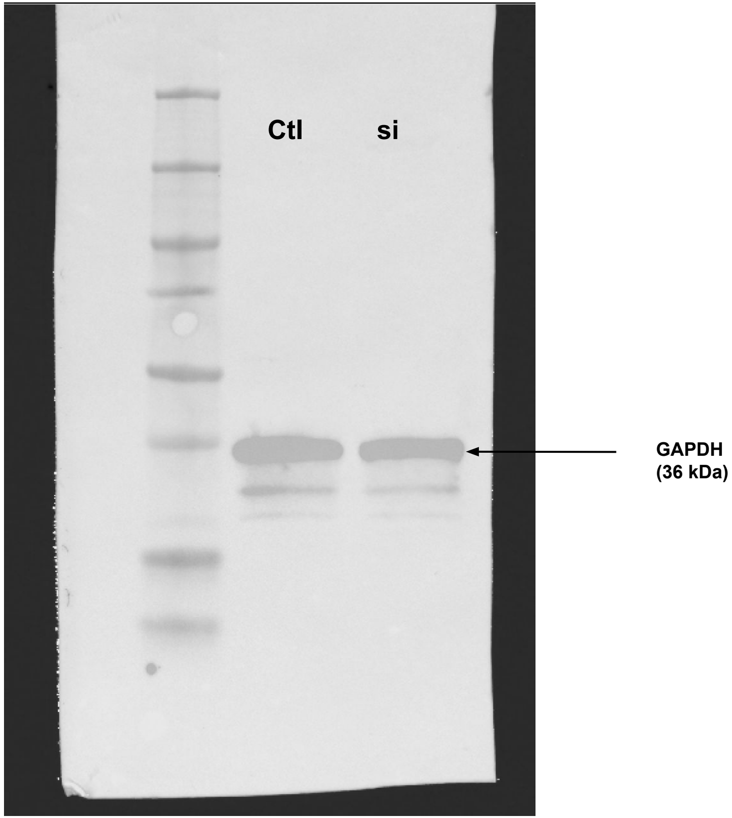

MDA-MB-231 cells_CREB3L1_replicate2

Figure 4

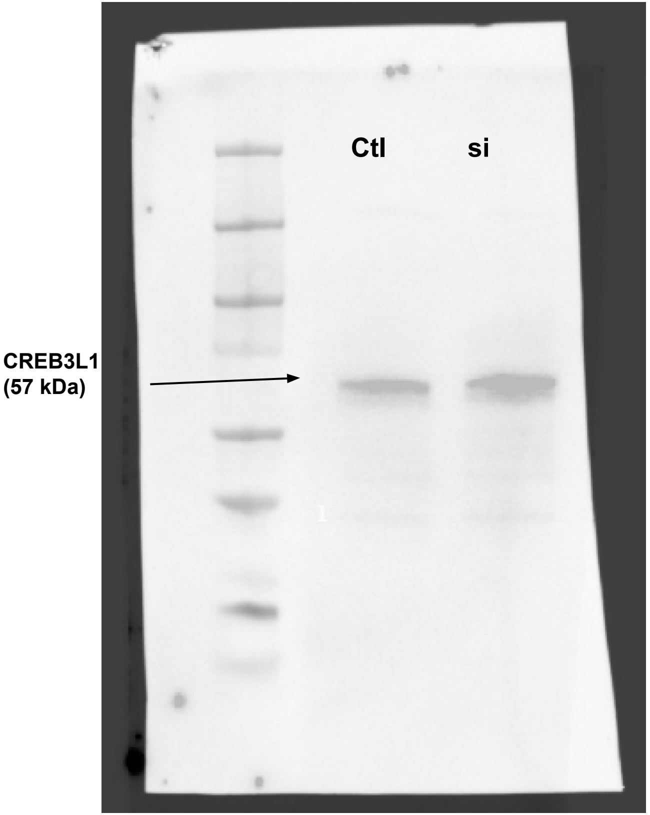

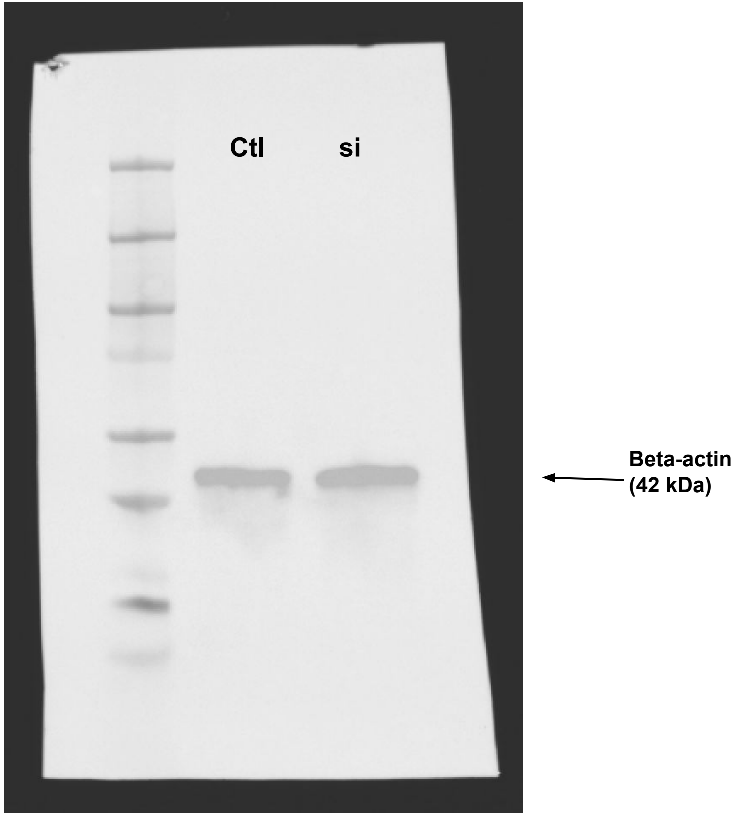

MDA-MB-231 cells_CREB3L1_replicate3

Figure 4

CREB3L1
(57 kDa)

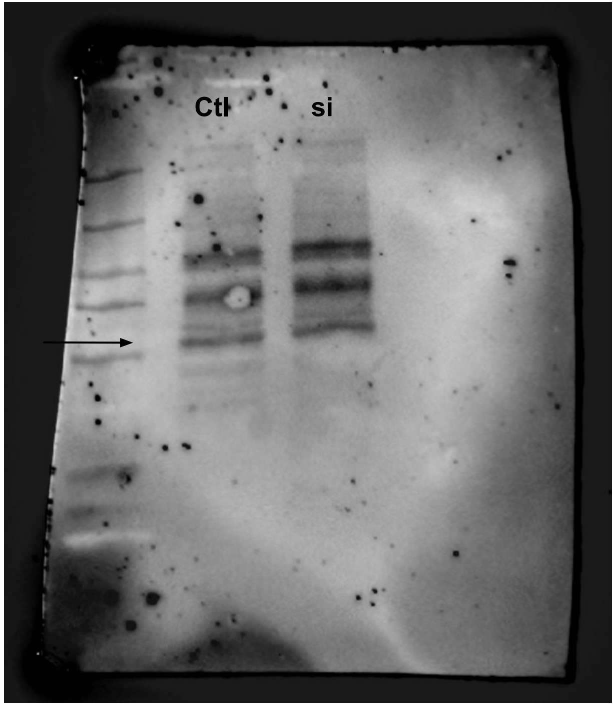

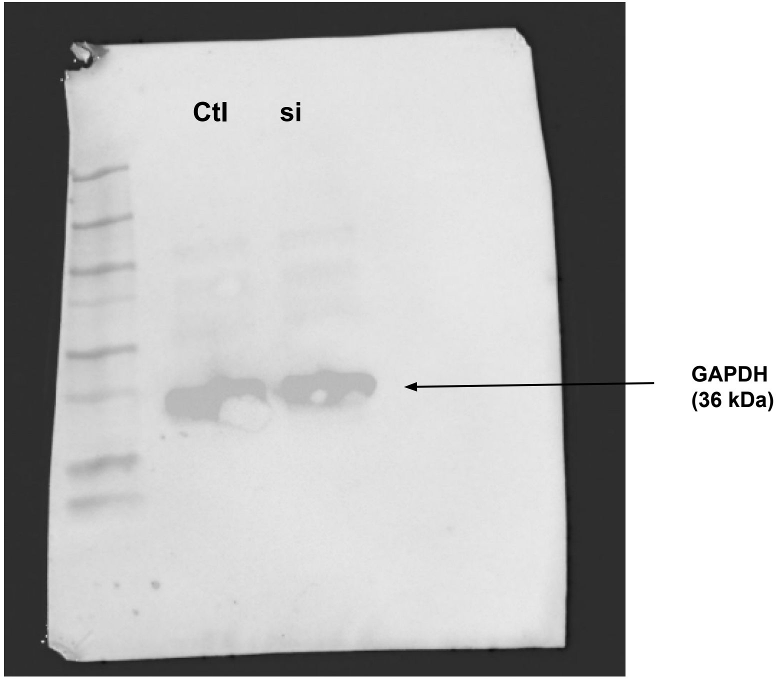

GAPDH
(36 kDa)

HCC1806 cells_CREB3L1_replicates1&2

Figure 4

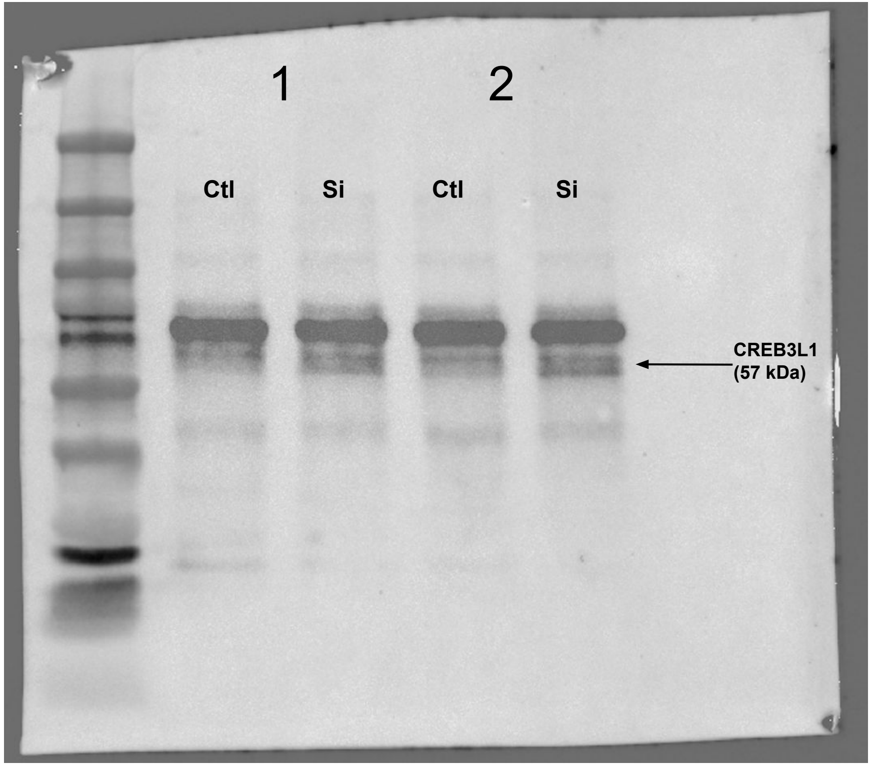

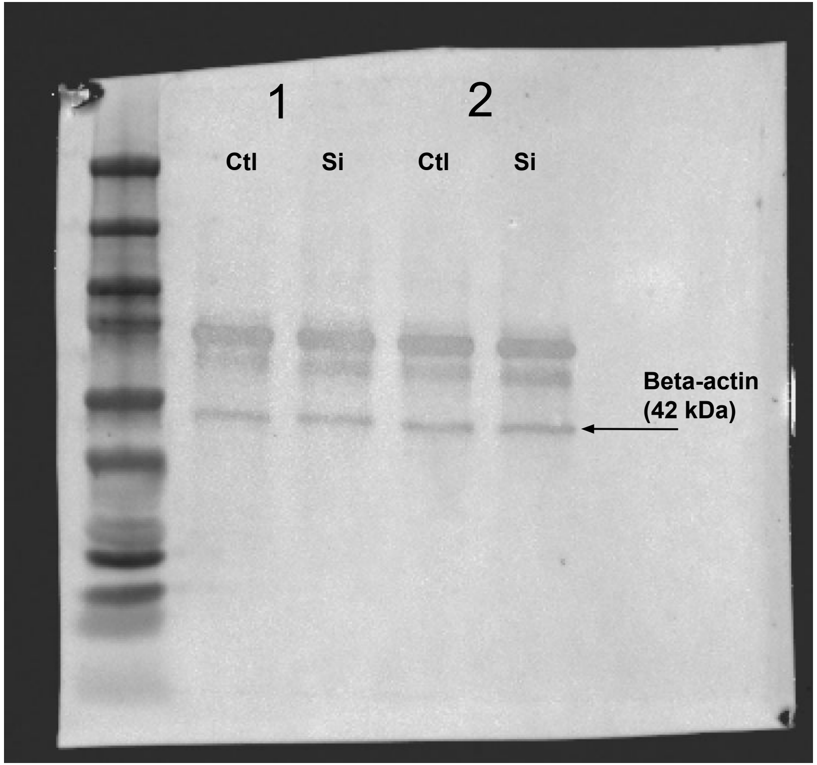

HCC1806 cells_CREB3L1_replicate3

Figure 4

- Representative image in Figure 4a of the manuscript

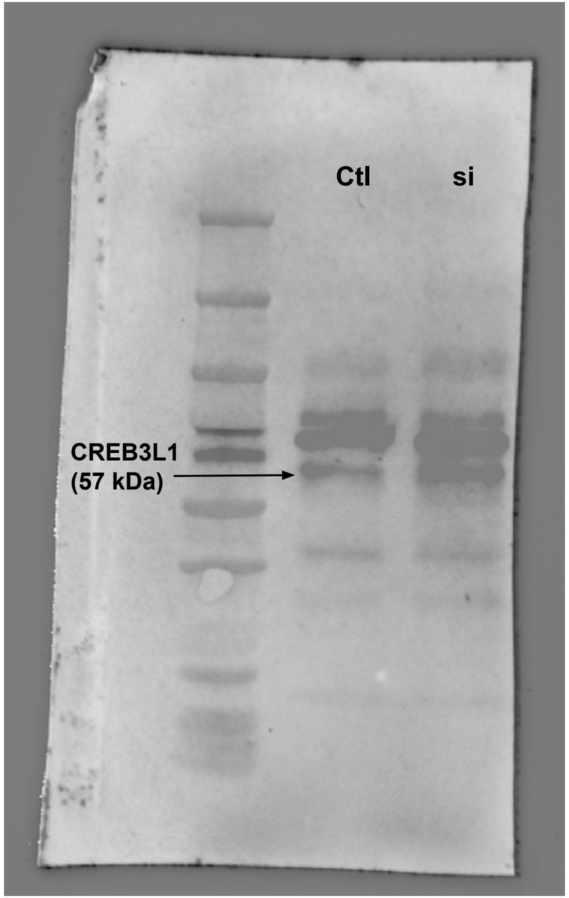

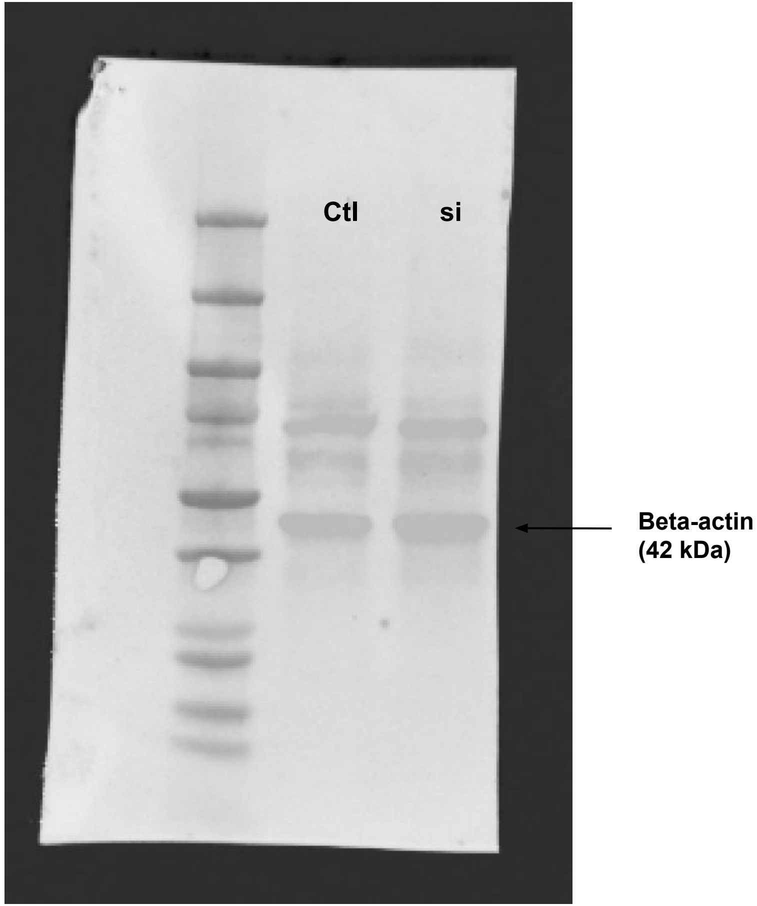

MDA-MB-231 cells_DNMT3A_replicate1

- Representative image Figure 1A

Figure 1

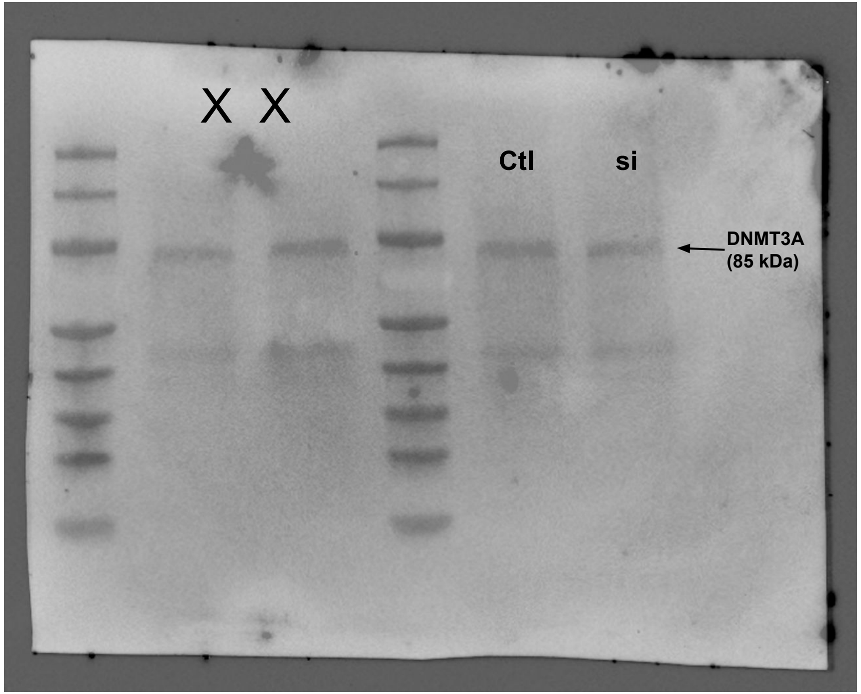

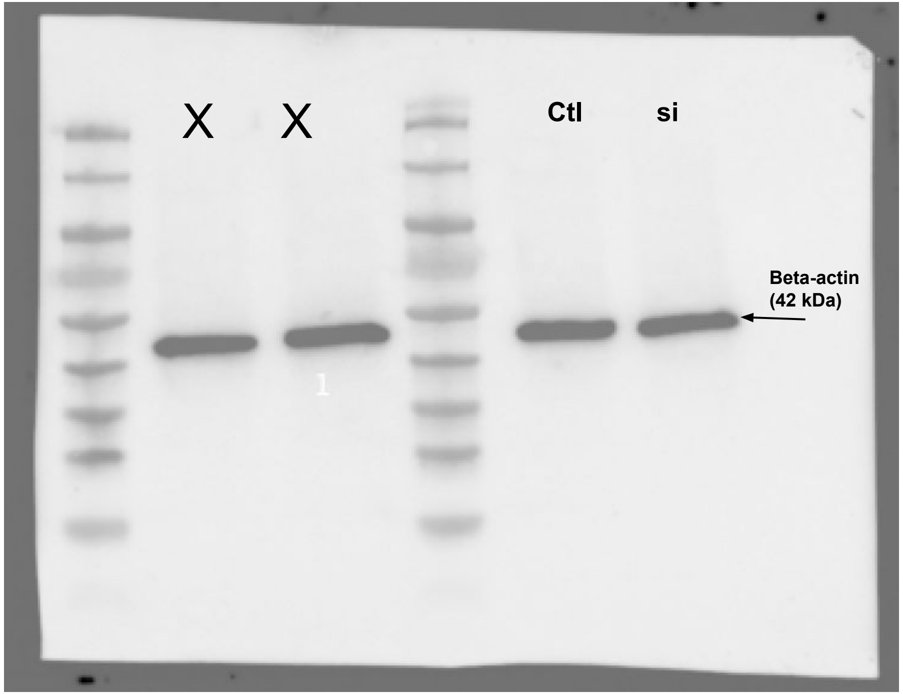

MDA-MB-231 cells_DNMT3A_replicate2

Figure 1

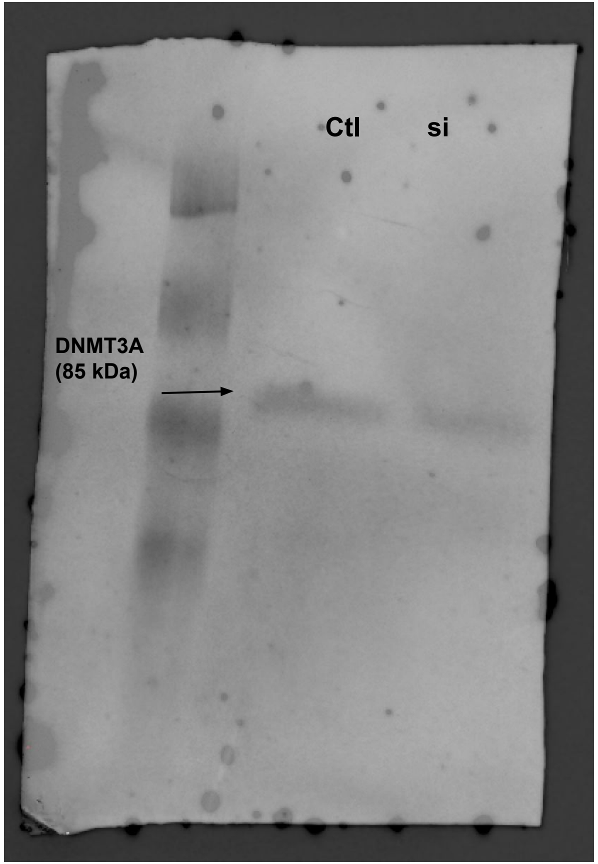

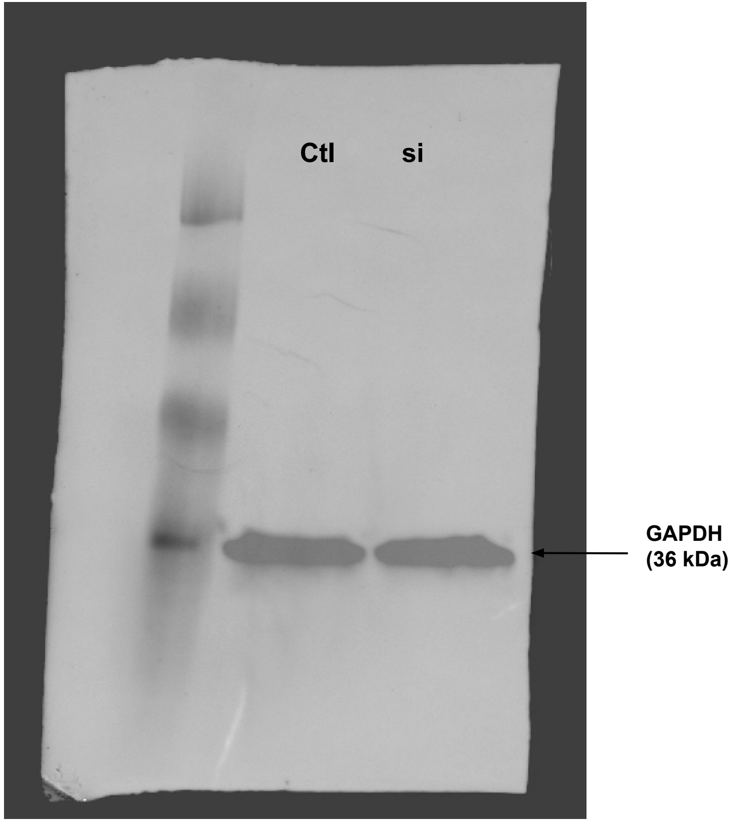

MDA-MB-231 cells_DNMT3A_replicate3

Figure 1

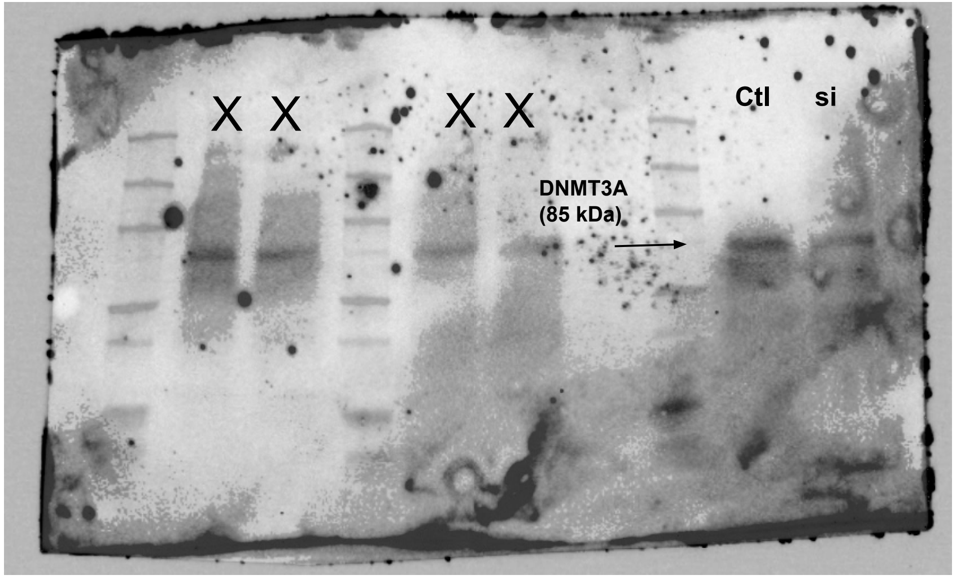

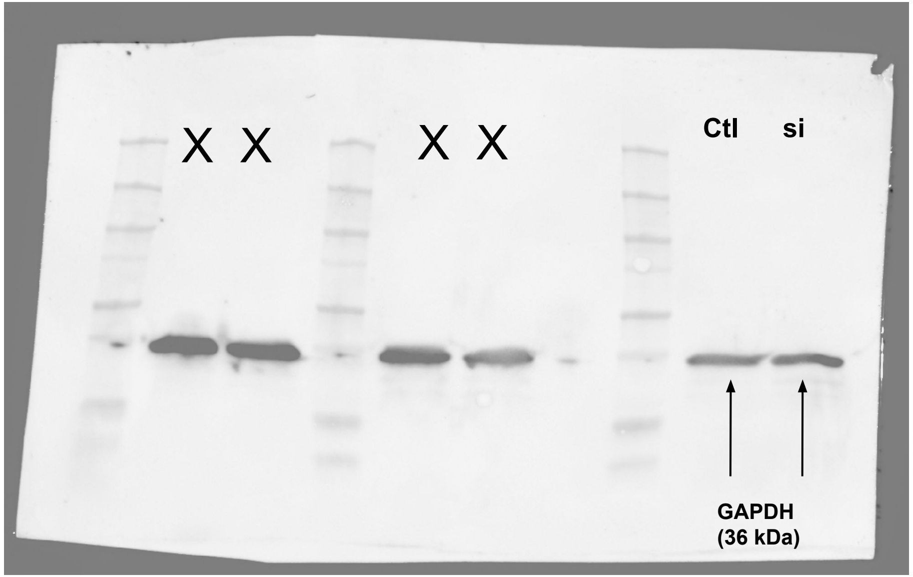

HCC1806 cells_DNMT3A_replicate1

Figure 1

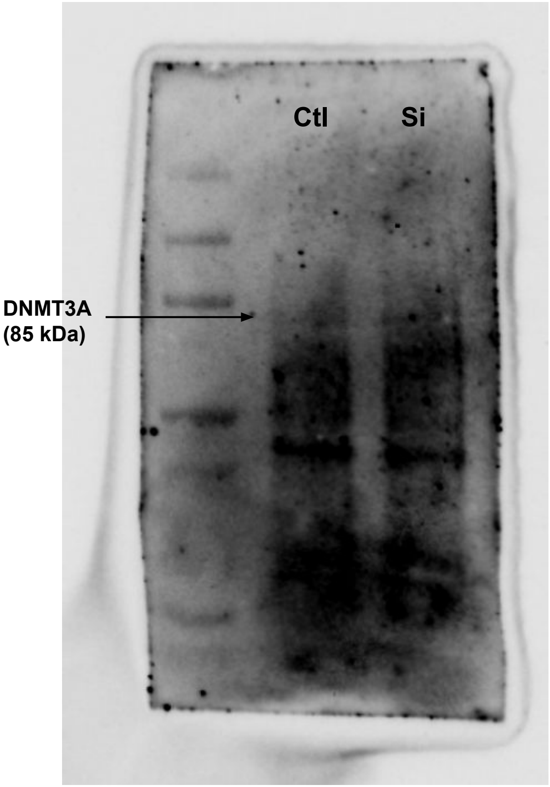

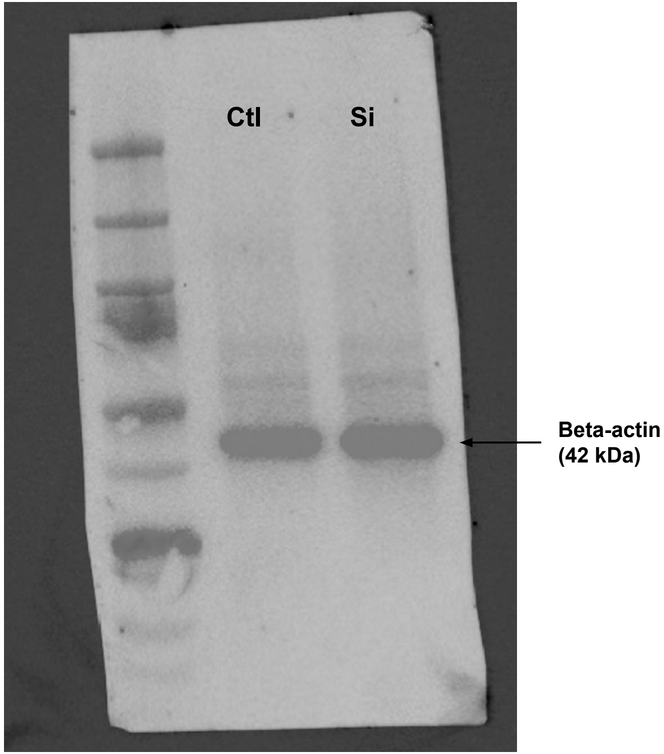

HCC1806 cells_DNMT3A_replicates2&3

Figure 1

- Rep 2 is a representative image in Figure 1a of the manuscript

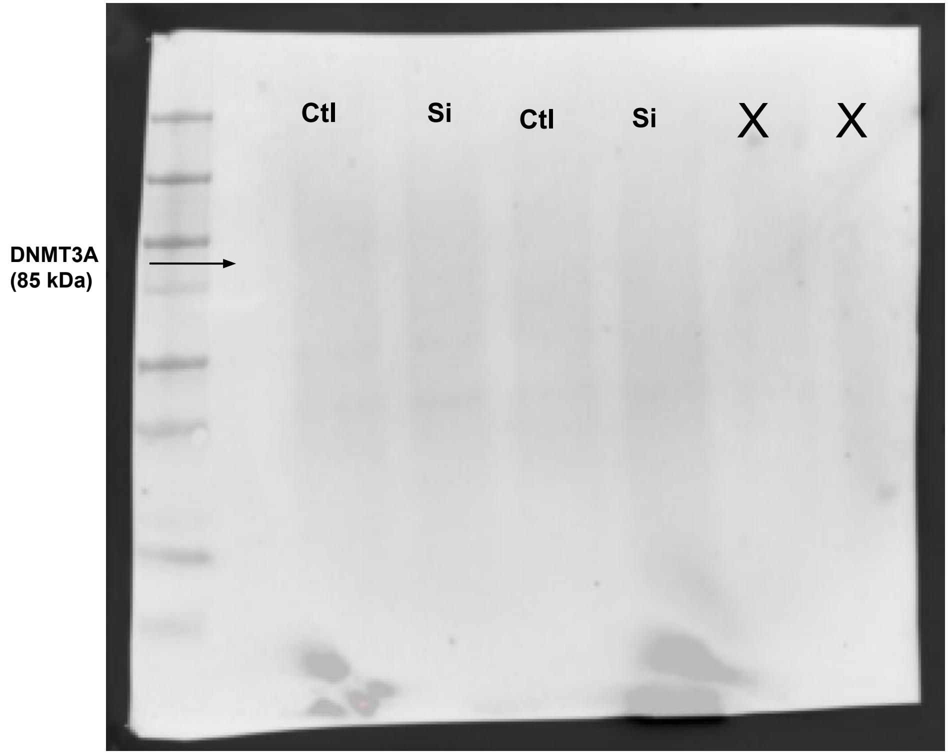

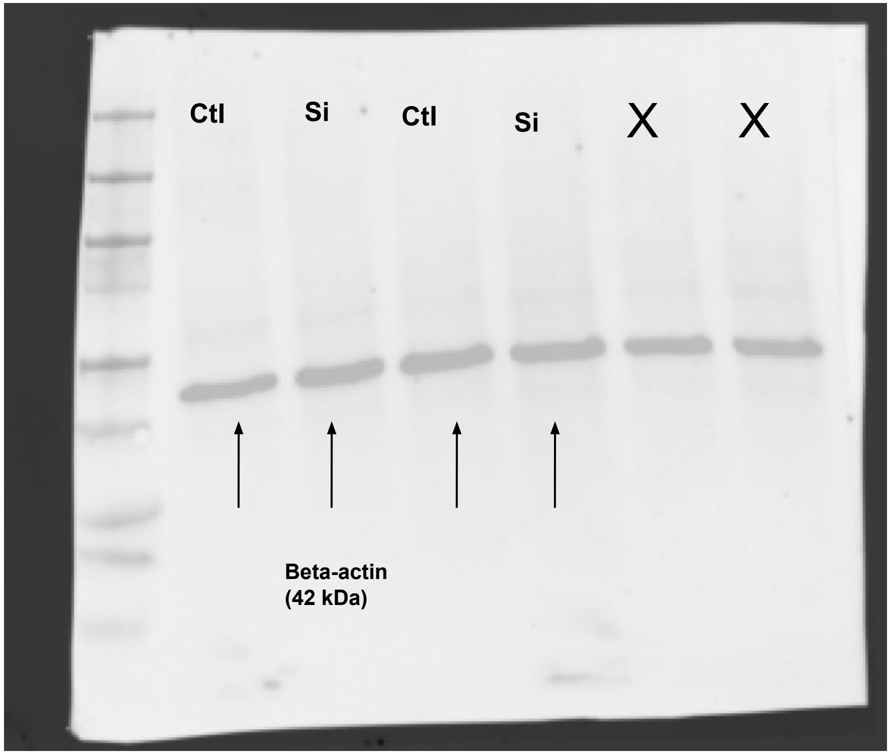

MDA-MB-231 cells_DNMT3B_replicate1

Figure 1

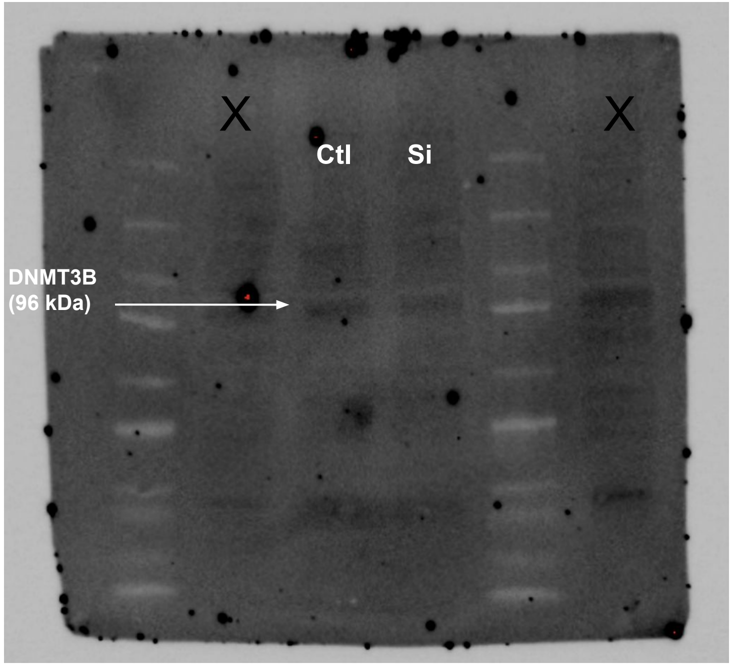

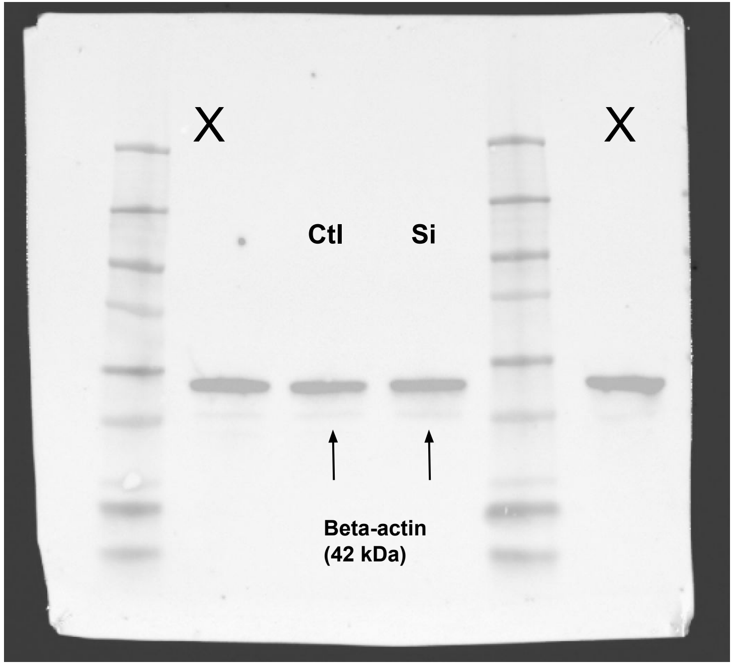

MDA-MB-231 cells_DNMT3B_replicate2

- Representative image in Figure 1a of the manuscript

Figure 1

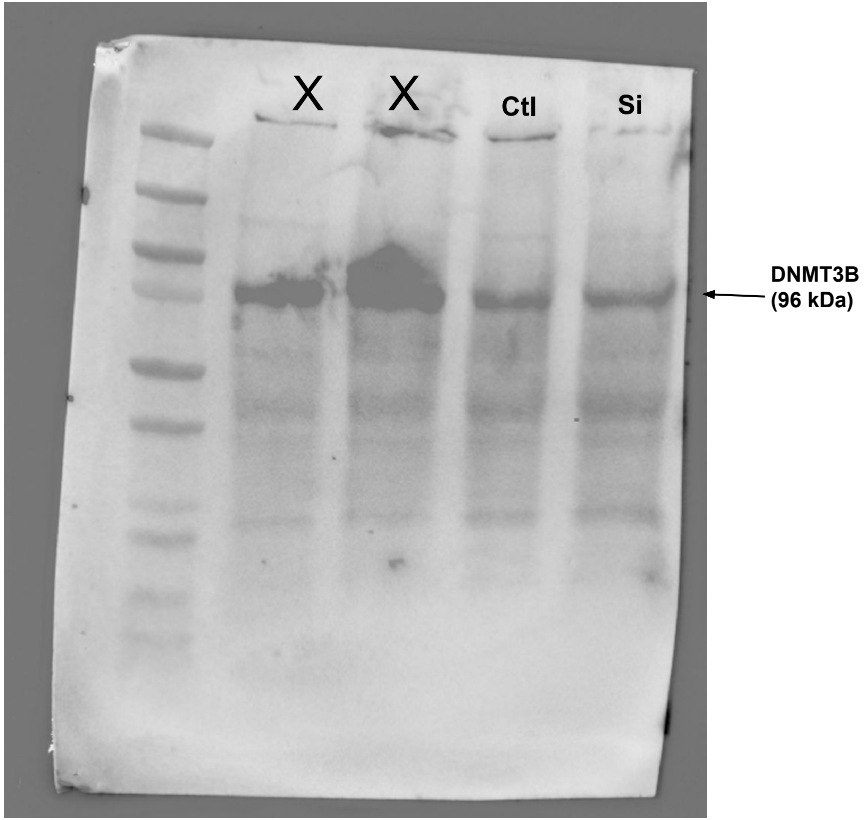

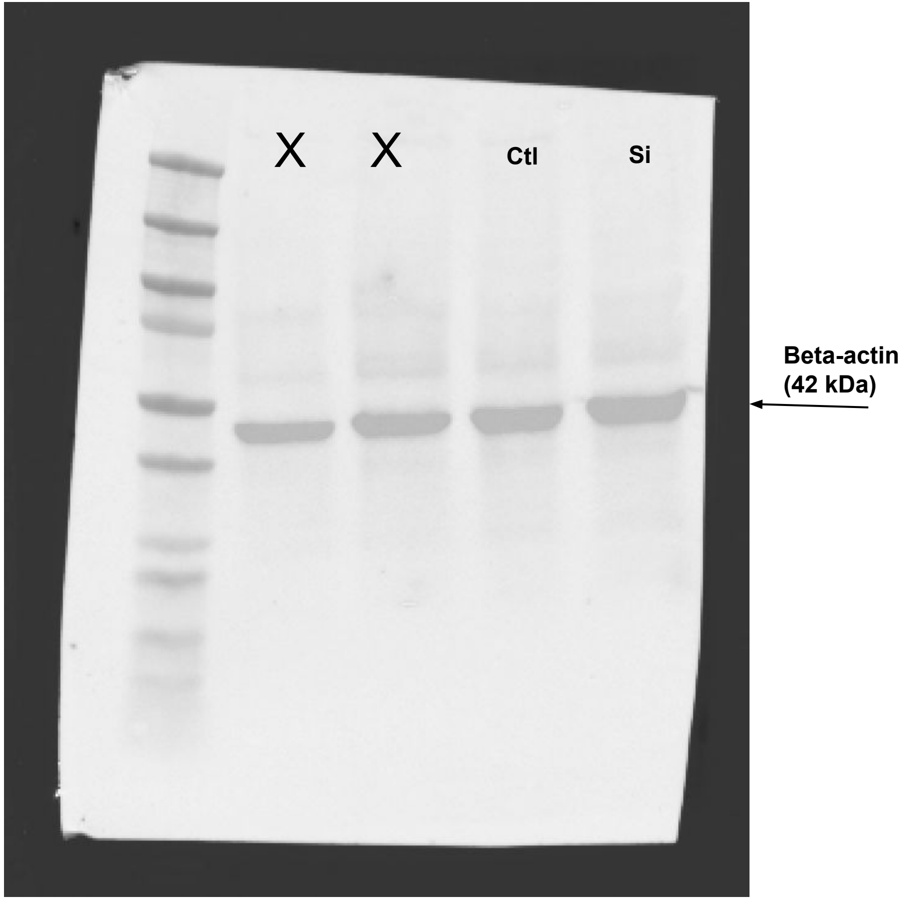

MDA-MB-231 cells_DNMT3B_replicate3

Figure 1

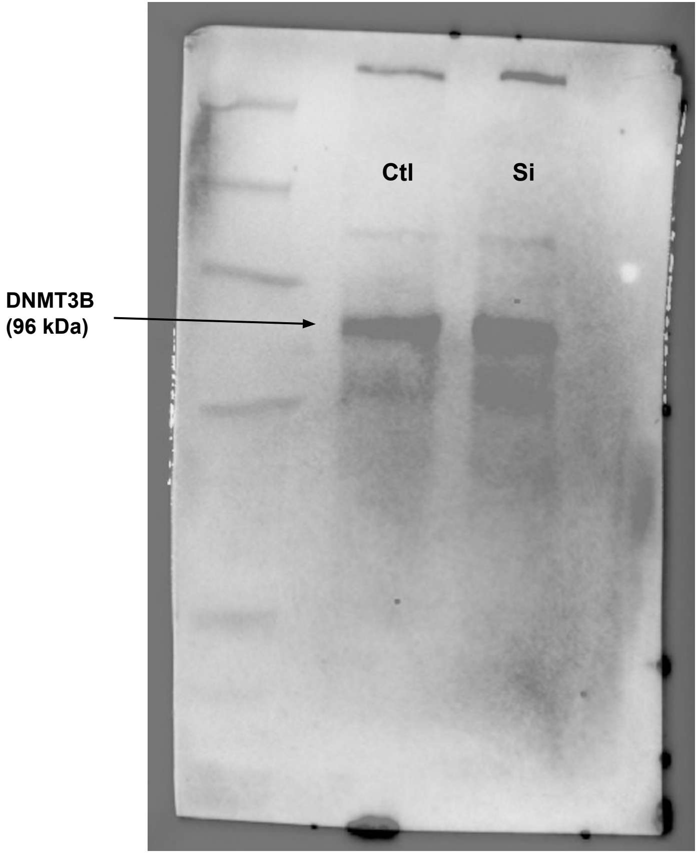

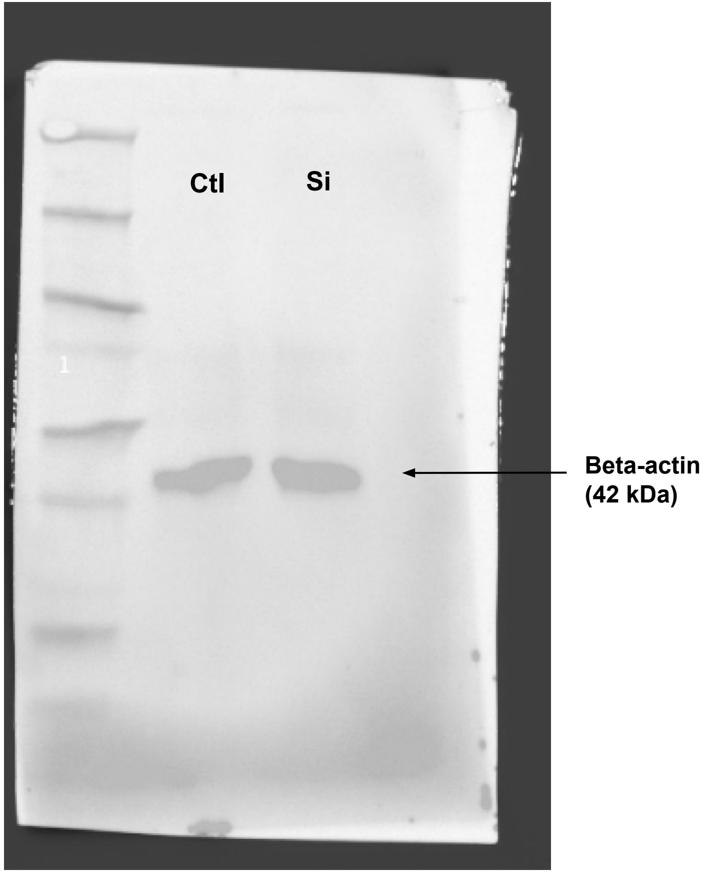

HCC1806 cells_DNMT3B_replicates1&2 Figure 1

- Rep 2 is a representative image in Figure 1a of the manuscript

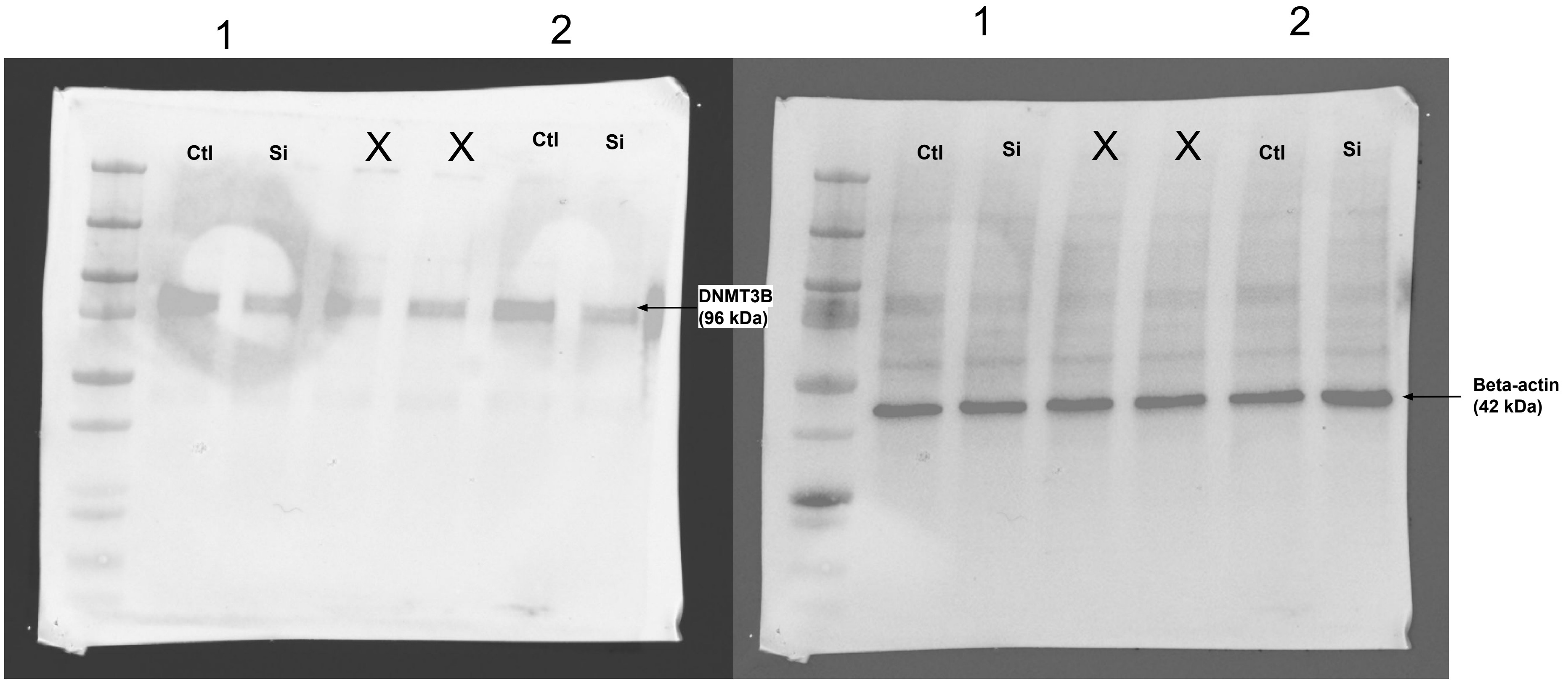

HCC1806 cells_DNMT3B_replicate3

Figure 1

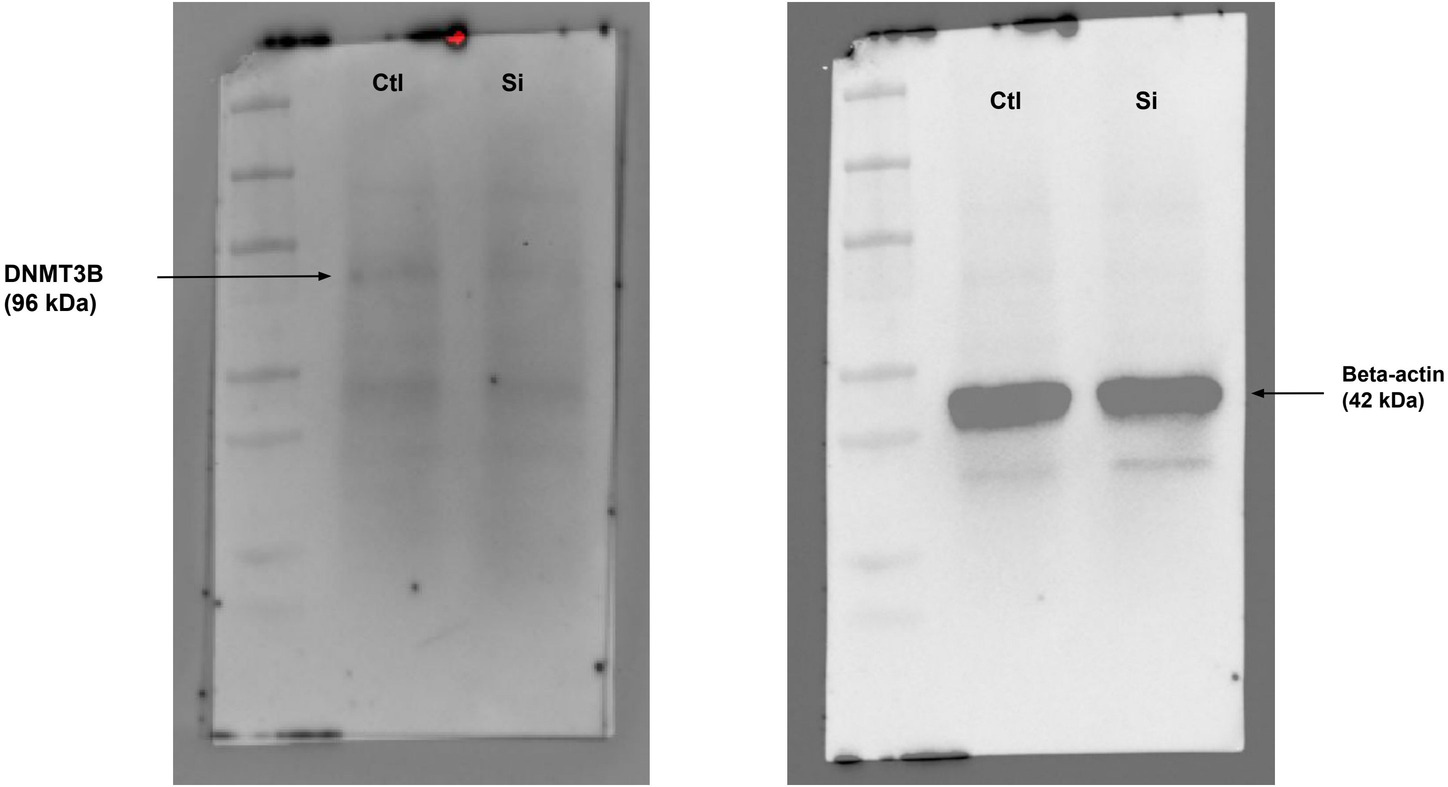

MDA-MB-231 cells_DNMT1_replicate1

Figure 1

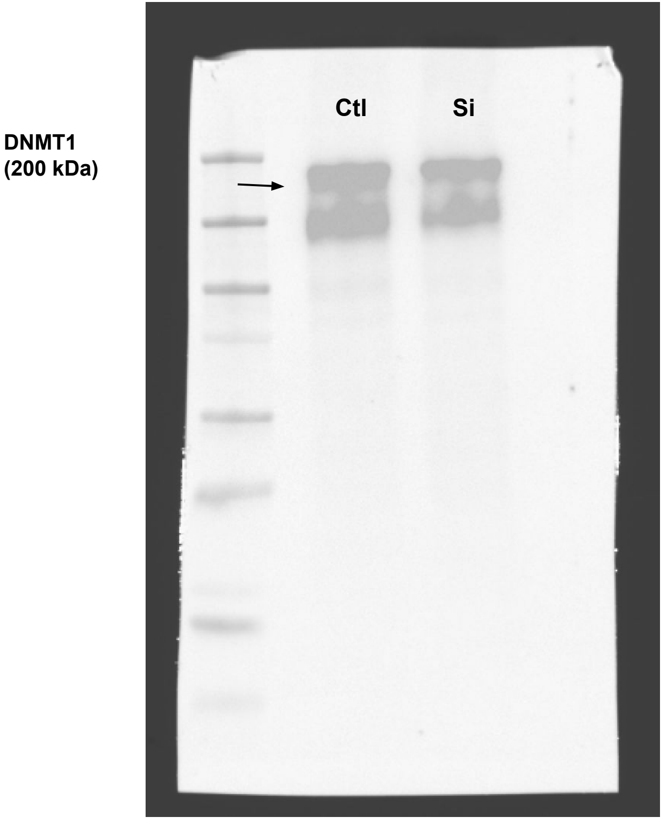

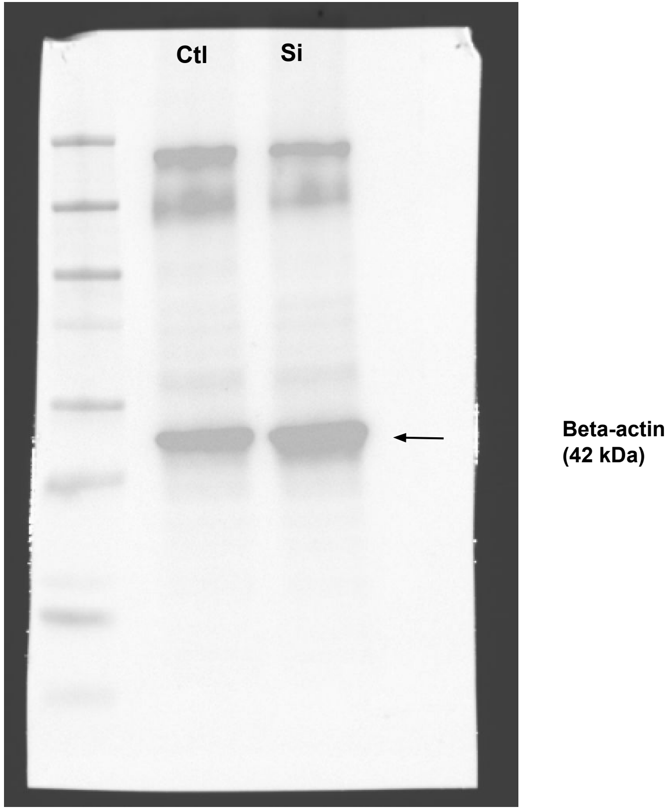

MDA-MB-231 cells_DNMT1_replicate2

Figure 1

DNMT1
(200 kDa)

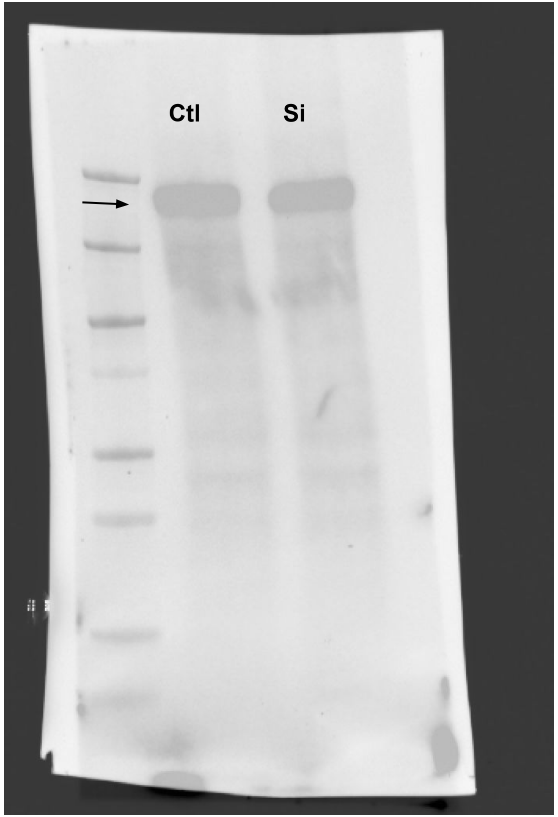

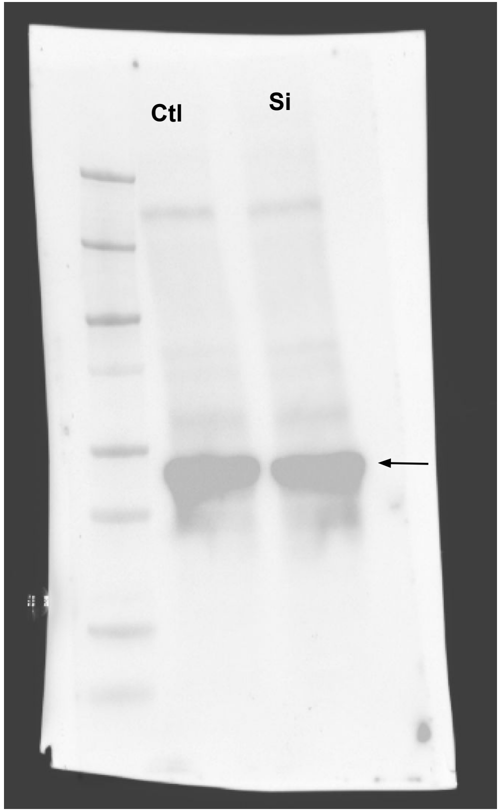

Beta-actin
(42 kDa)

MDA-MB-231 cells_DNMT1_replicate3

Figure 1

- Representative image in Figure 1a of the manuscript

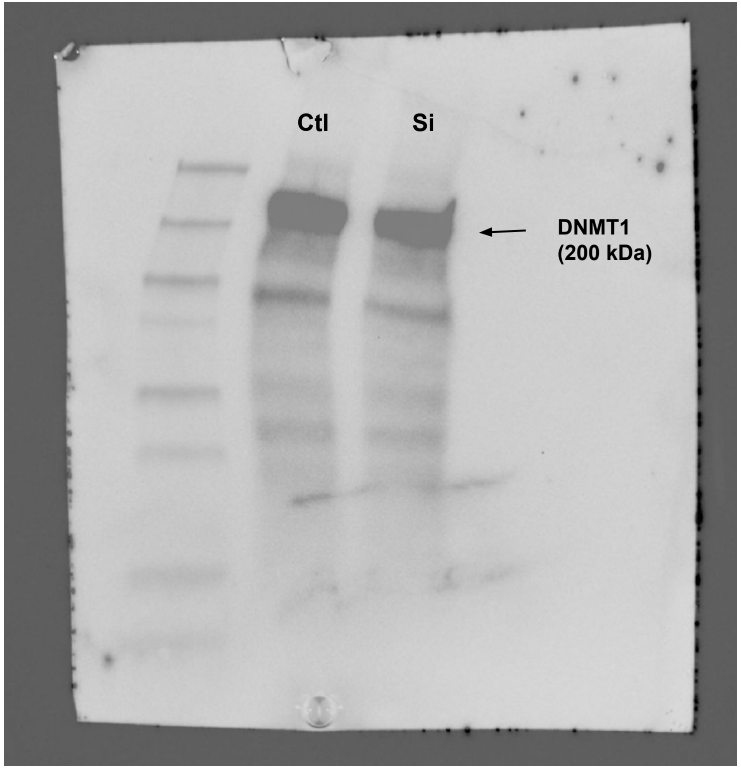

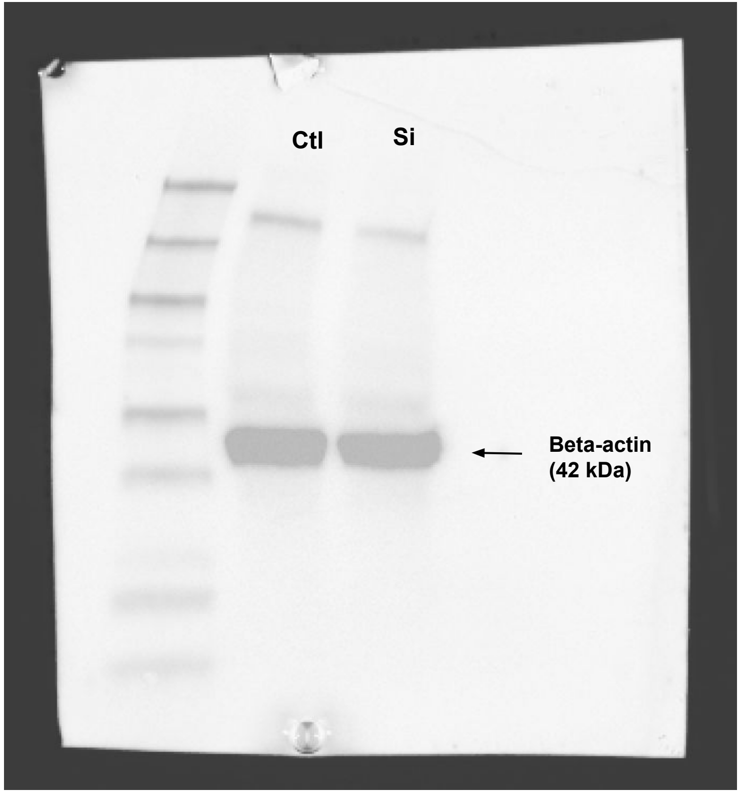

HCC1806 cells_DNMT1_replicate1

Figure 1

- Representative image Figure 1a

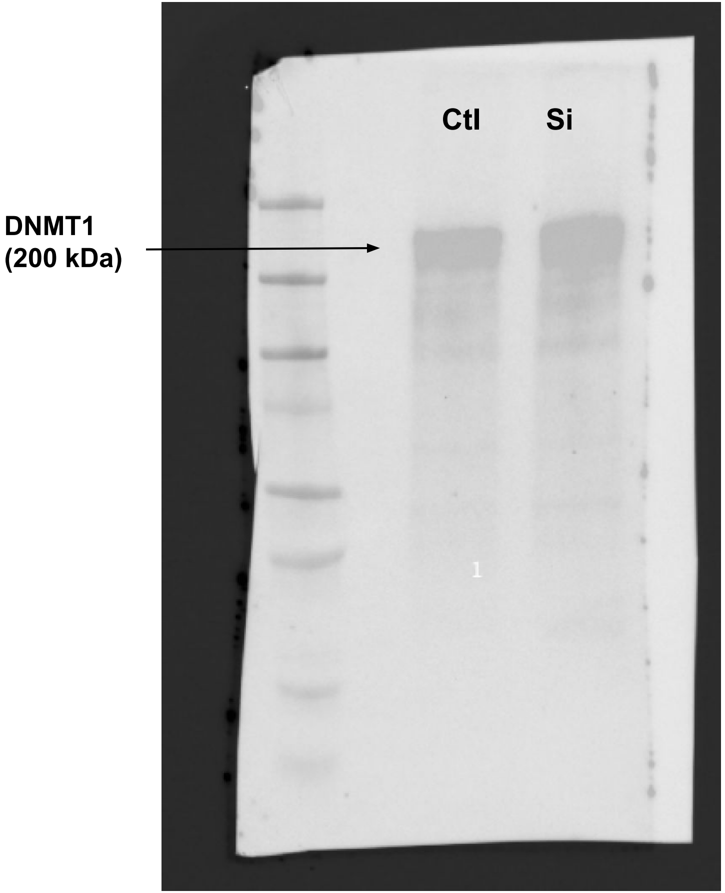

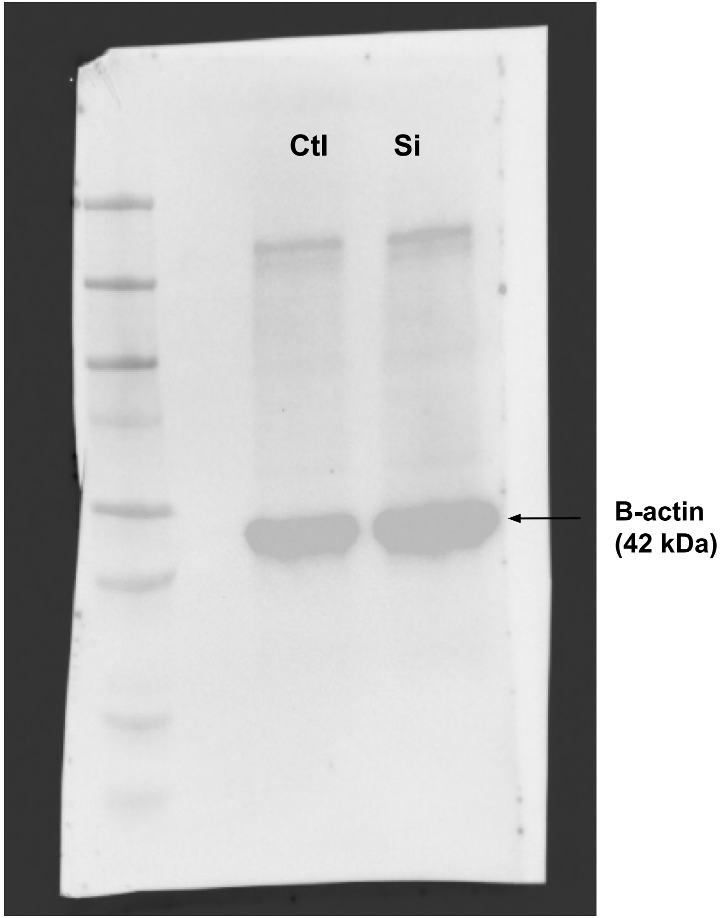

HCC1806 cells_DNMT1_replicates2&3 Figure 1

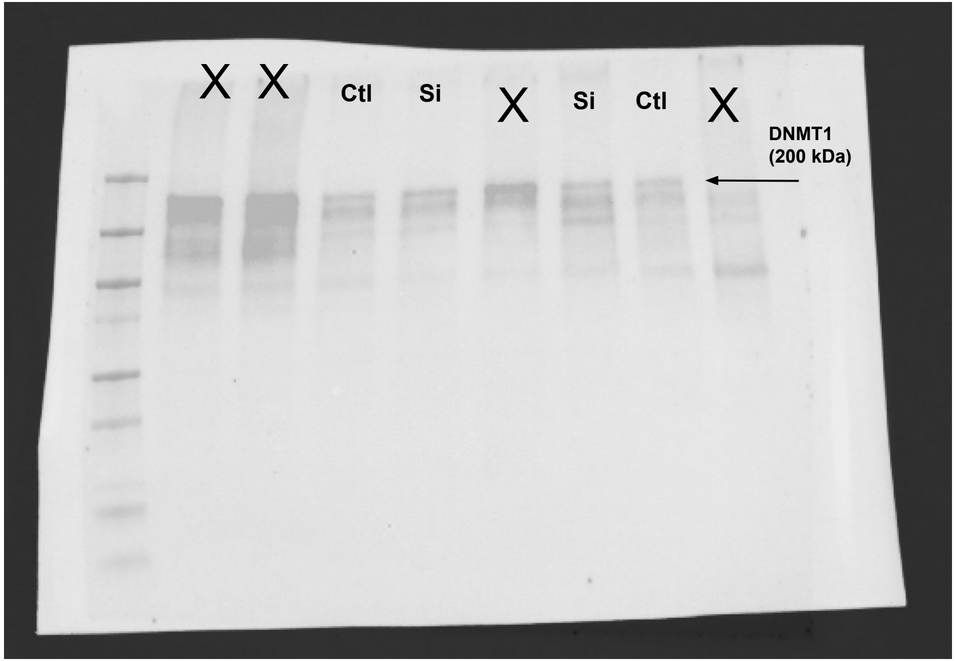

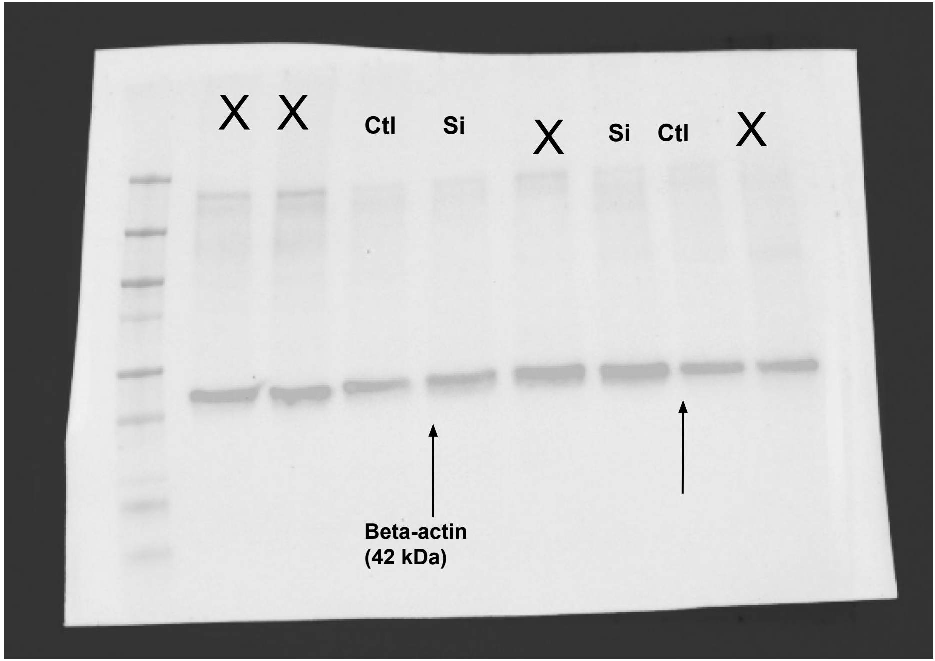

MDA-MB-231 cells_STAT3_replicates1&2 Figure 5

- Replicate 1 was cropped in Figure 5a of the manuscript

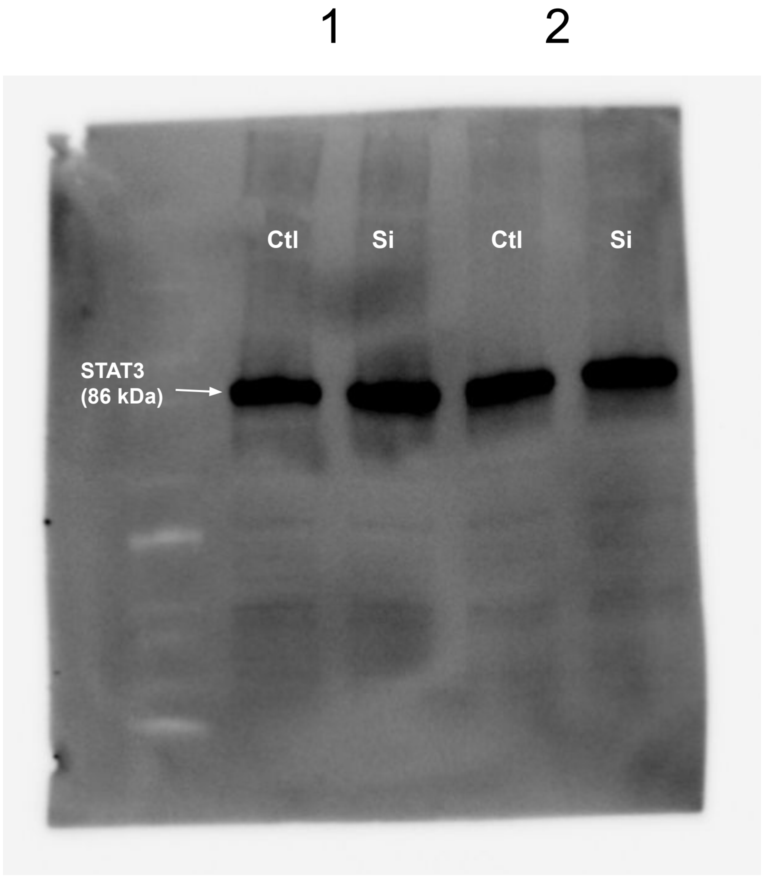

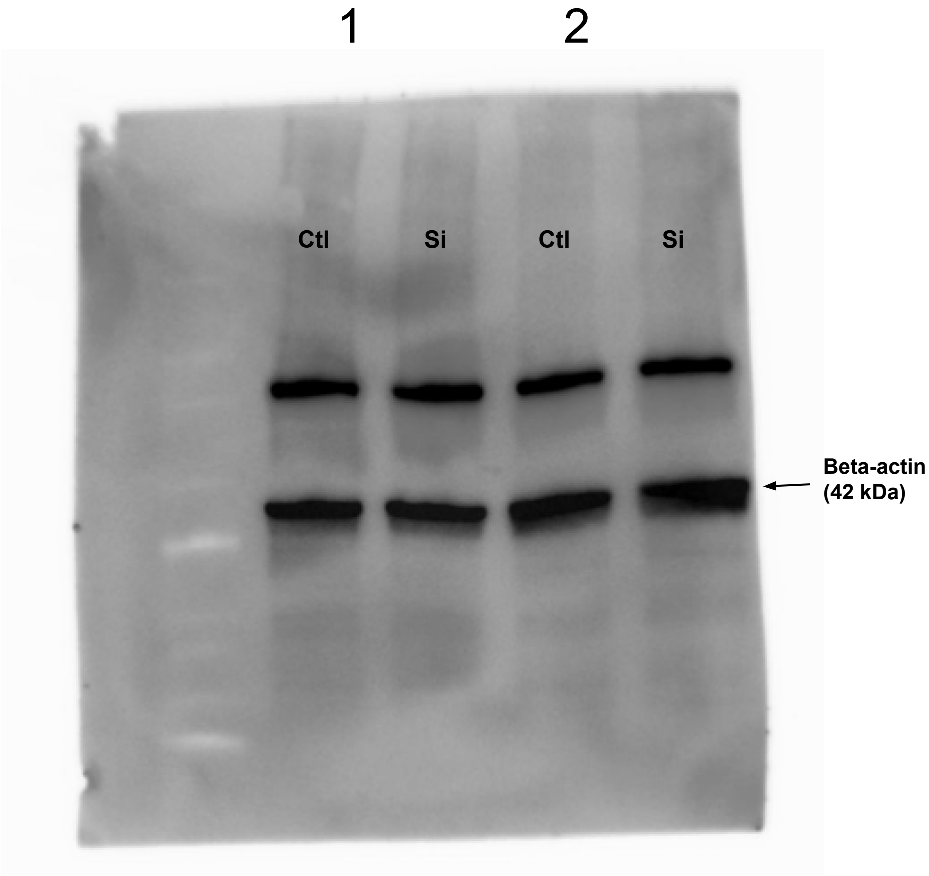

MDA-MB-231 cells_STAT3_replicate3 Figure 5

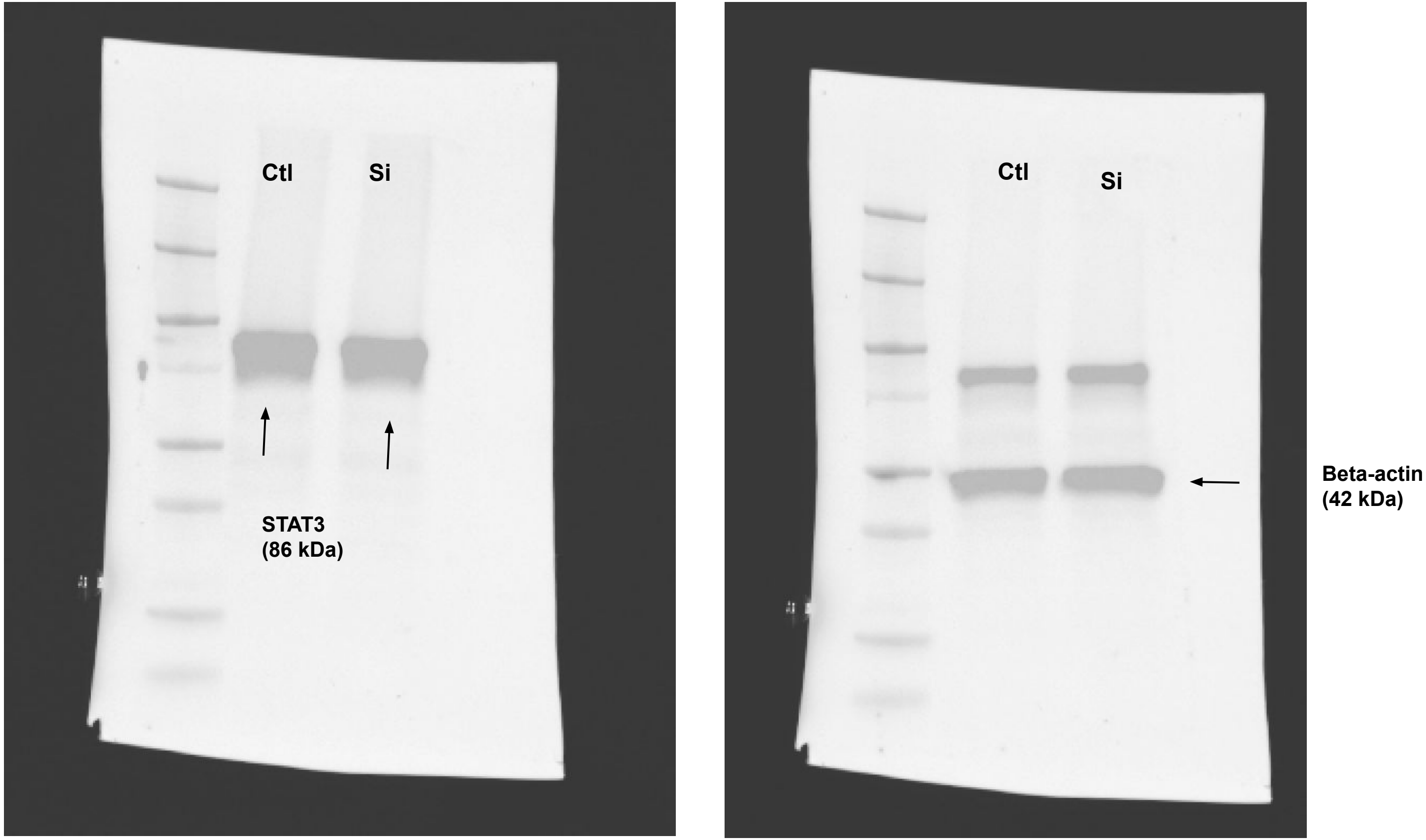

HCC1806 cells_STAT3_replicate1 Figure 5

- Replicate 1 was used in figure 5

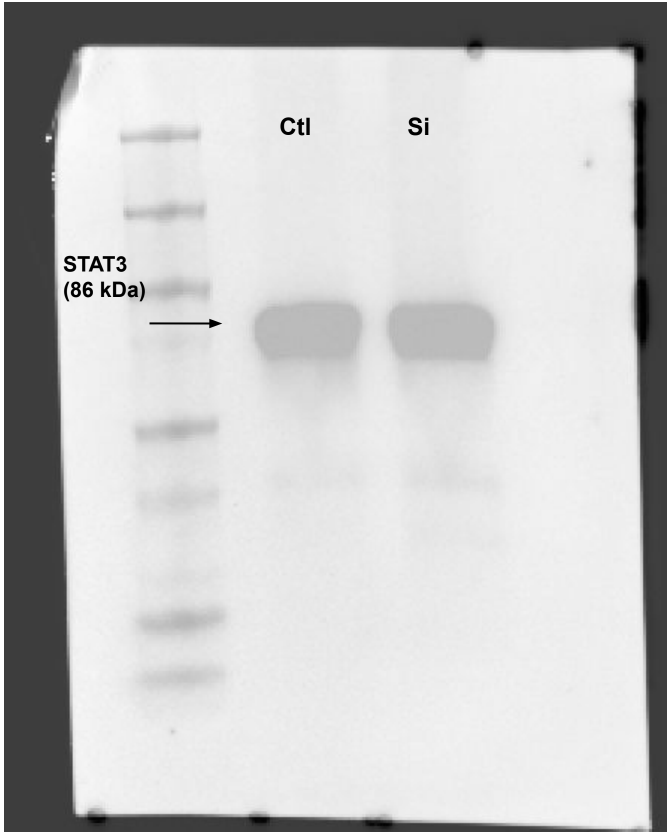

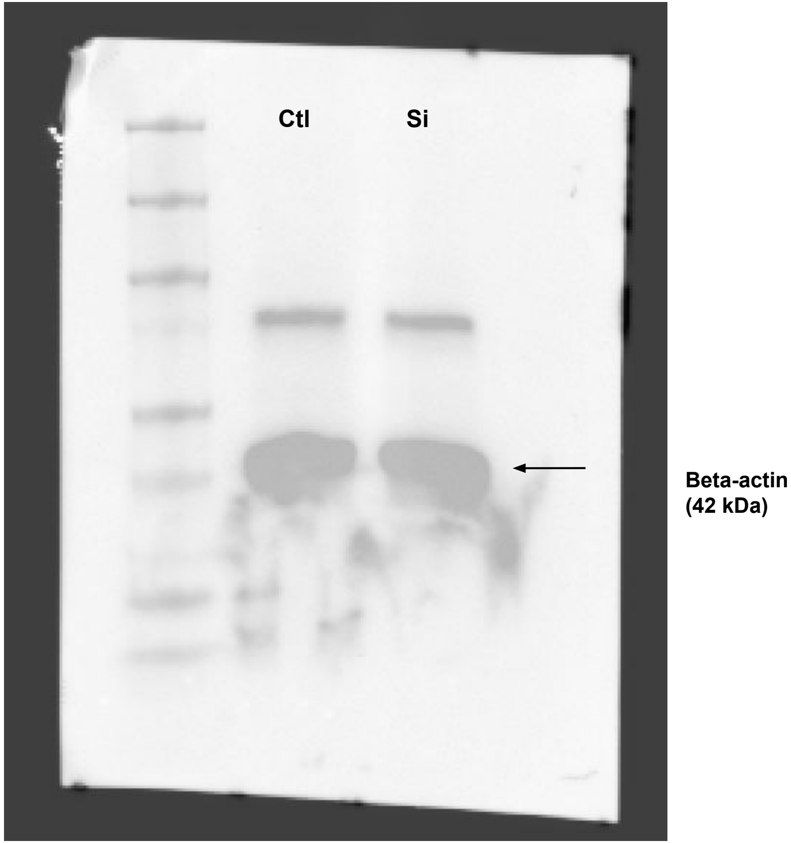

HCC1806 cells_STAT3_replicate2 Figure 5

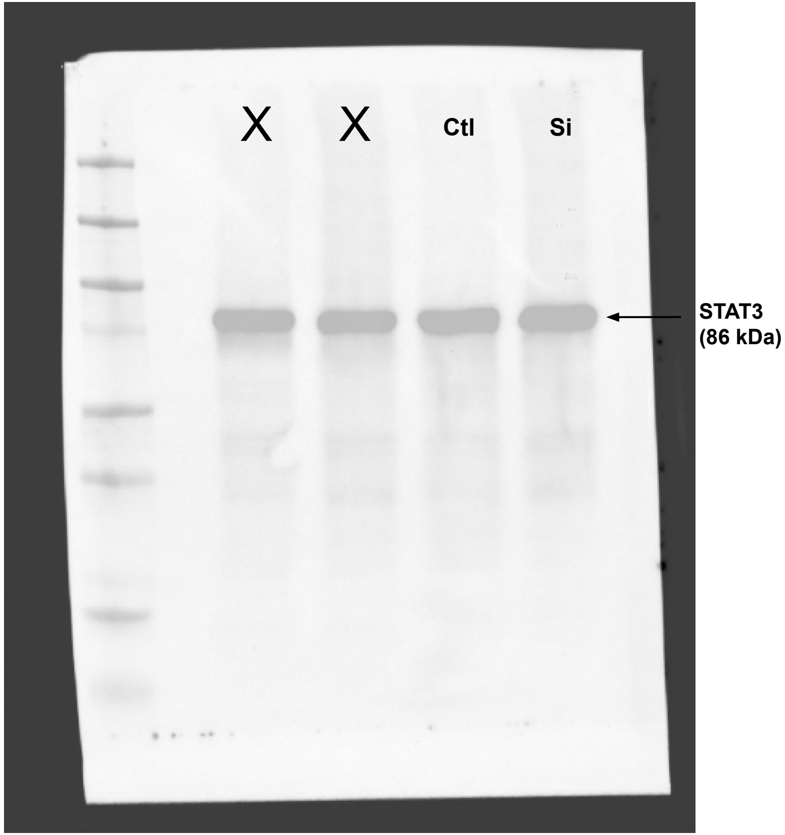

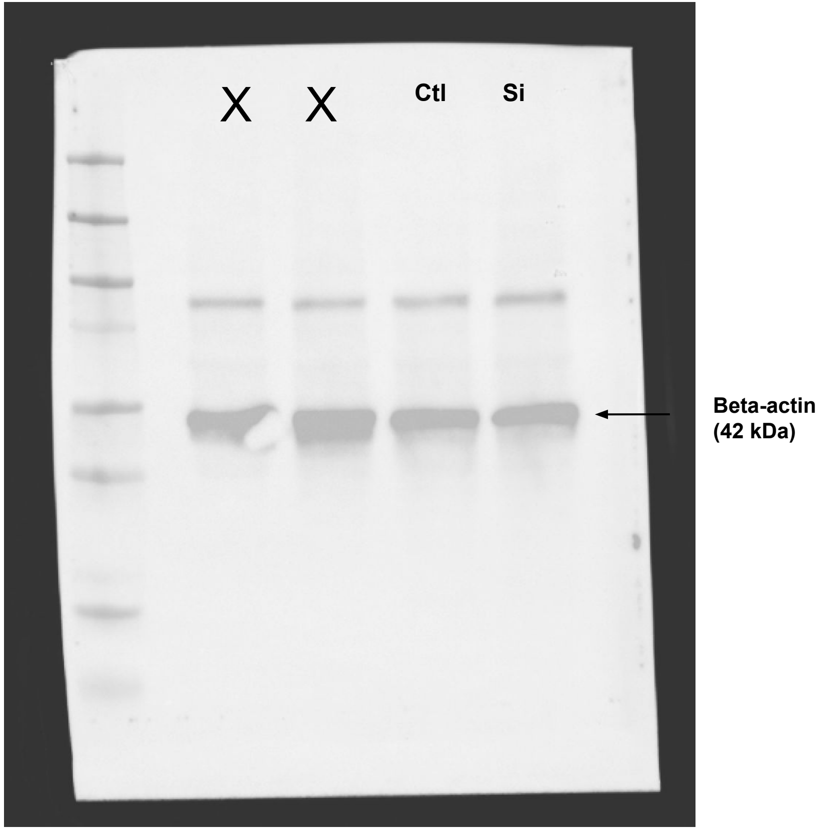

HCC1806 cells_STAT3_replicate3

Figure 5

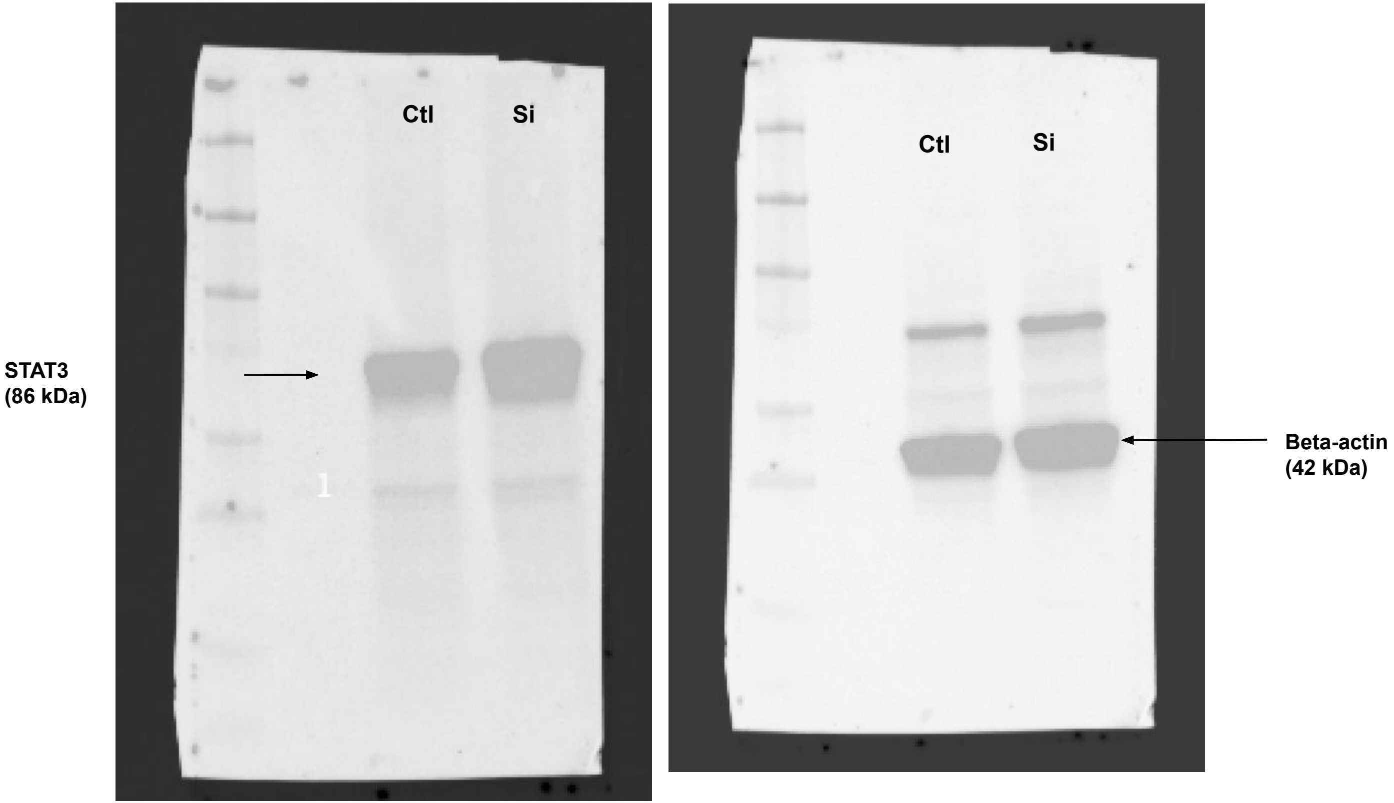

MDA-MB-231 cells_pSTAT3_replicate1 Figure 5

- Replicate 1 was used in Figure 5a

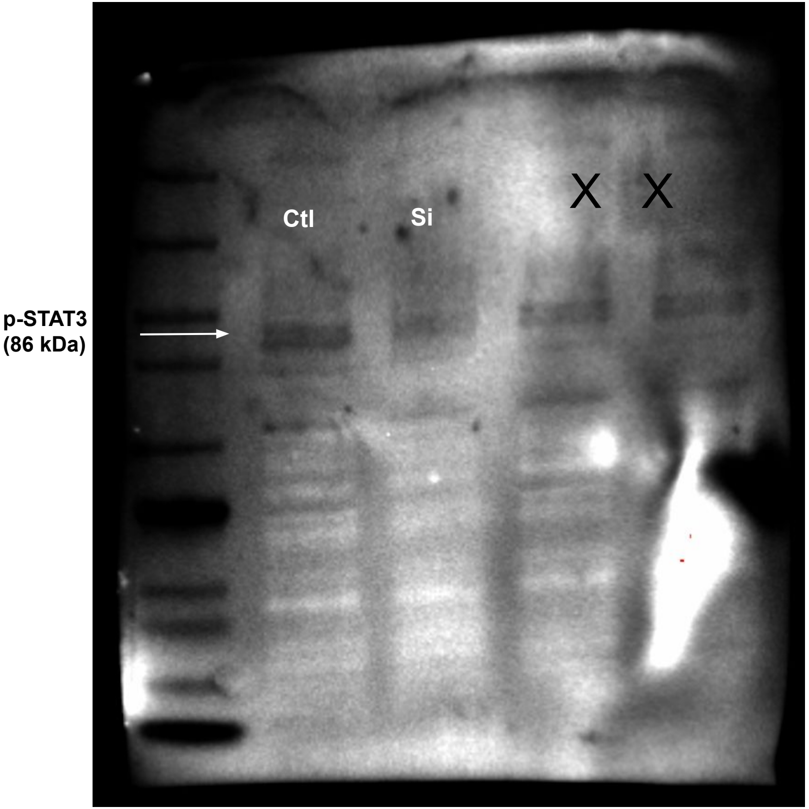

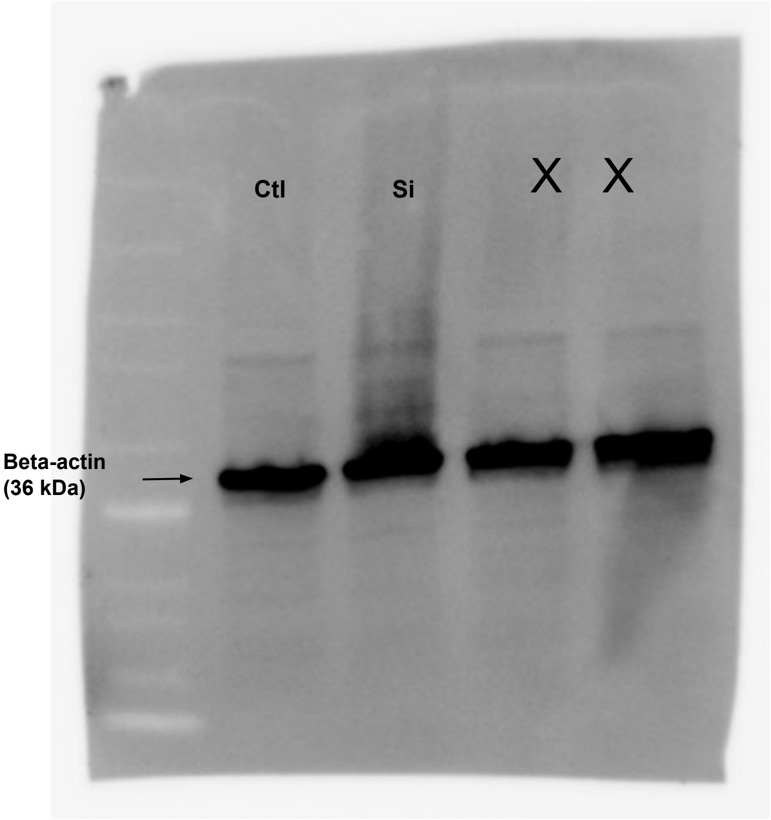

MDA-MB-231 cells_pSTAT3_replicate2

Figure 5

p-STAT3
(86 kDa)

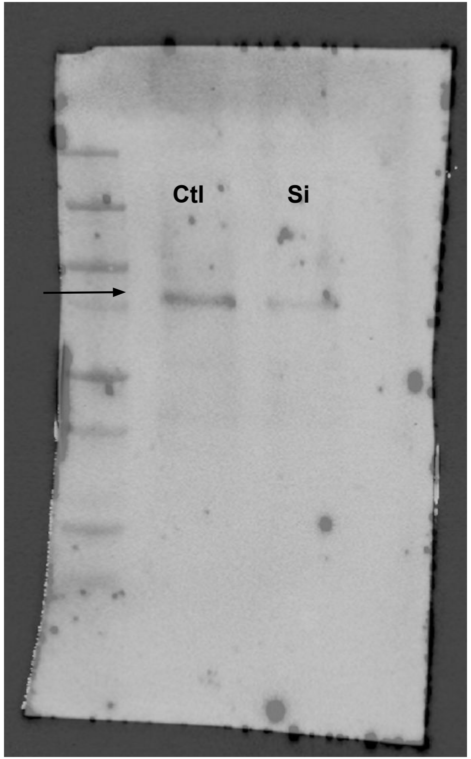

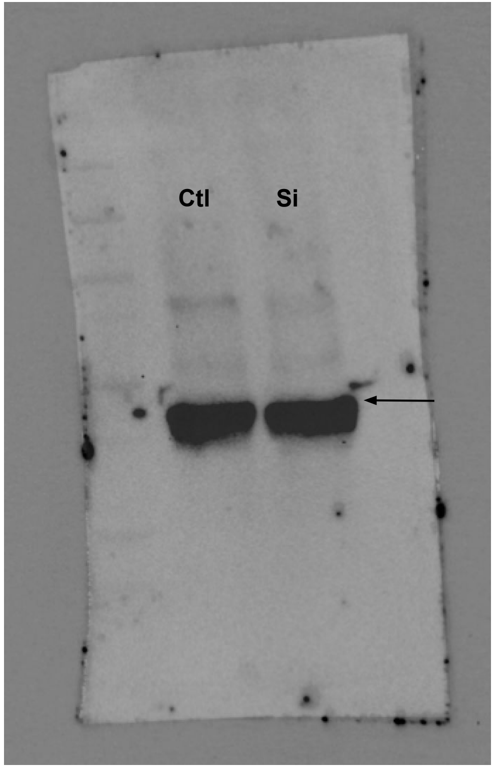

Beta-actin
(42 kDa)

HCC1806 cells_pSTAT3_replicates1&2 Figure 5

- Replicate 1 was used in figure 5a of the manuscript

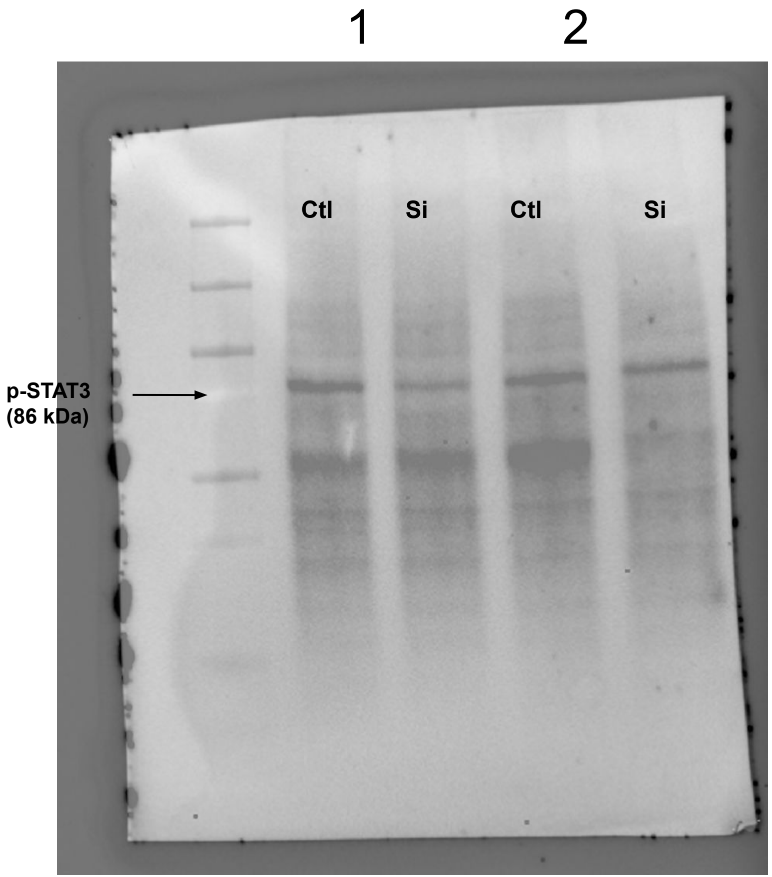

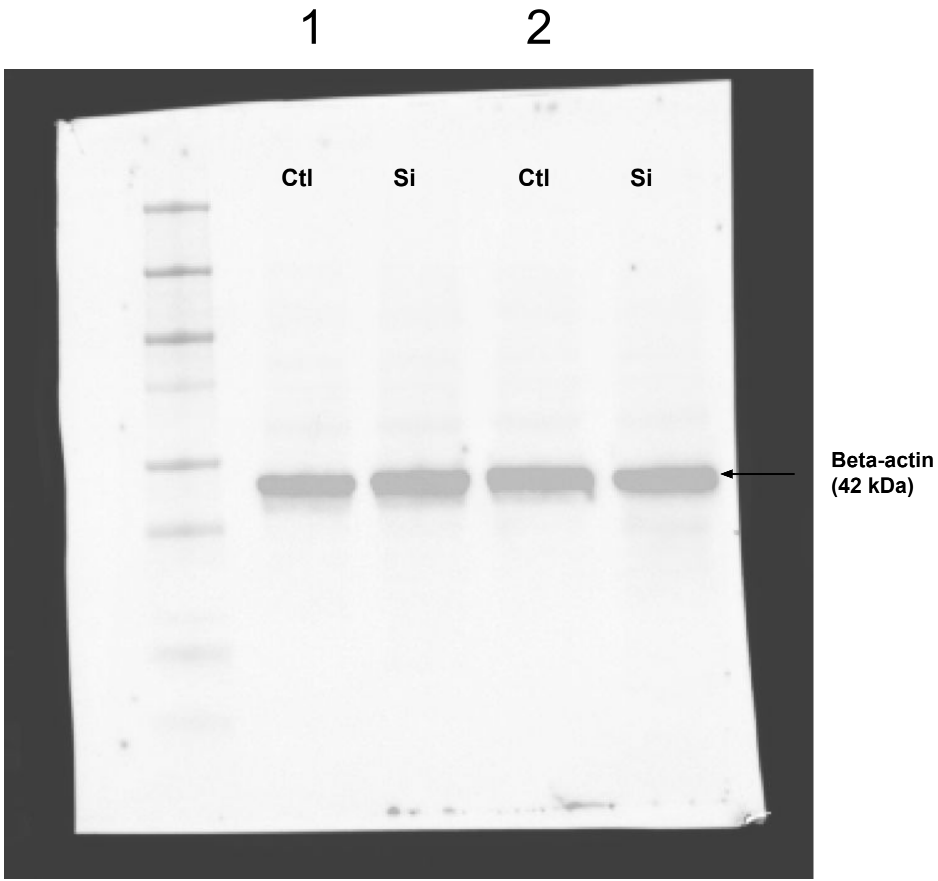

HCC1806 cells_pSTAT3_replicate3 Figure 5

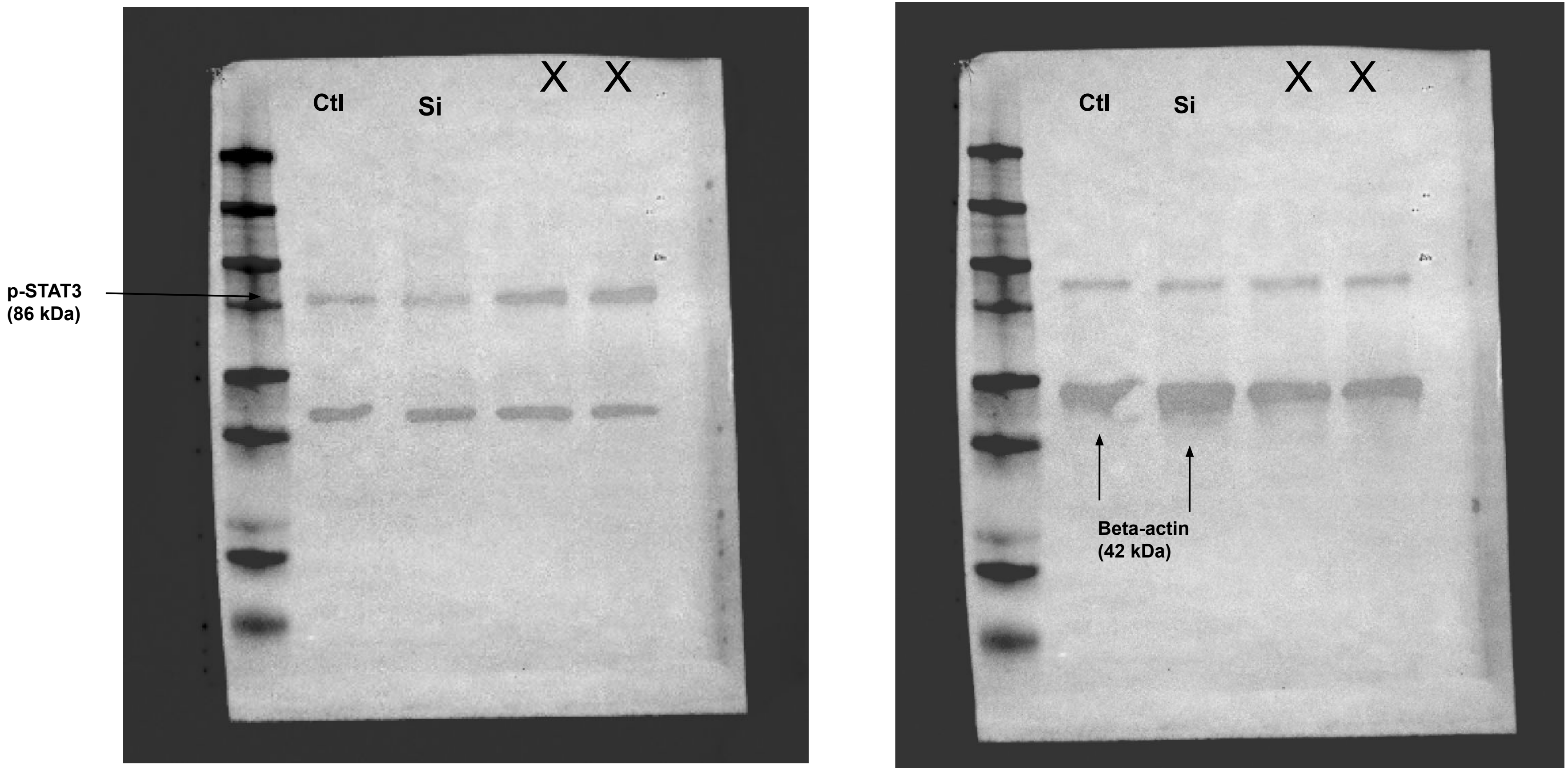

Raw western blot data

1. All lanes used in the manuscript and used for densitometry analysis are labeled. Unlabeled lanes were not used for analysis.
2. All proteins used in the manuscript and for analysis are labeled “ctl” or “IL6+”. Lanes labeled “X” were not utilized.
3. Protein lysates were obtained from MCF10A cell lines
4. Lanes labeled “IL6+” are proteins from cells lines that were transfected with IL-6 recombinant protein to induce a pSTAT3 activation. Lanes that are labeled “Ctl” are the control samples that are untreated.
5. All densitometry analysis was normalized to a housekeeping gene, beta-actin.
6. Brightness and contrast of blots may have been adjusted to make the protein bands more visible in the manuscript.

MCF10A cells_ROR1_replicate1

Figure 6

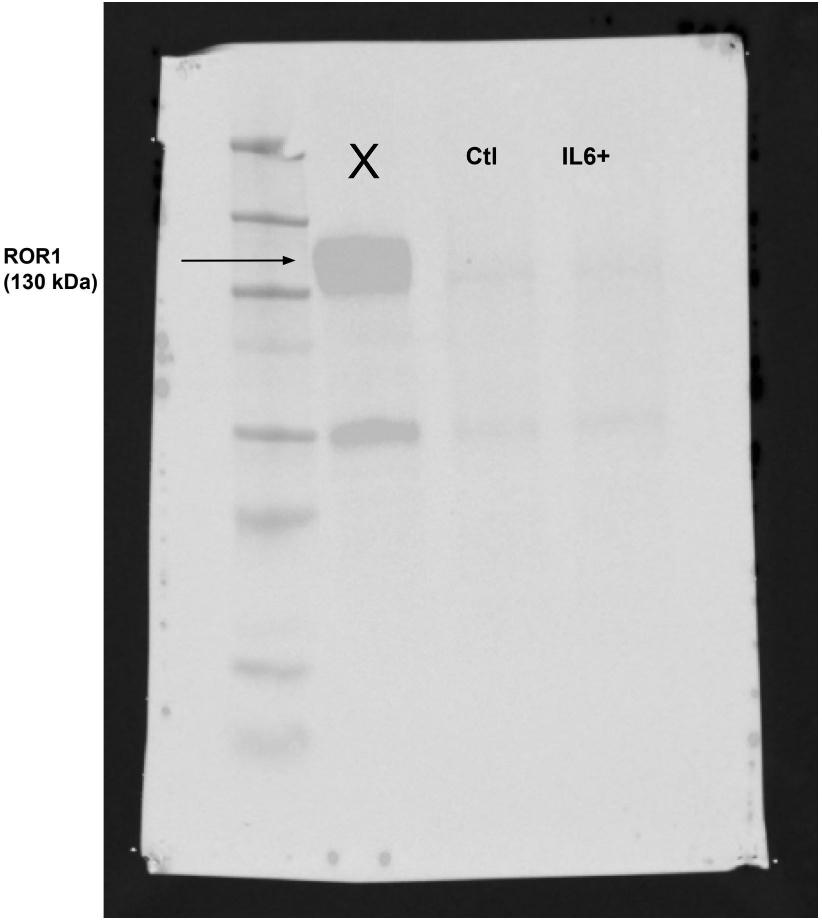

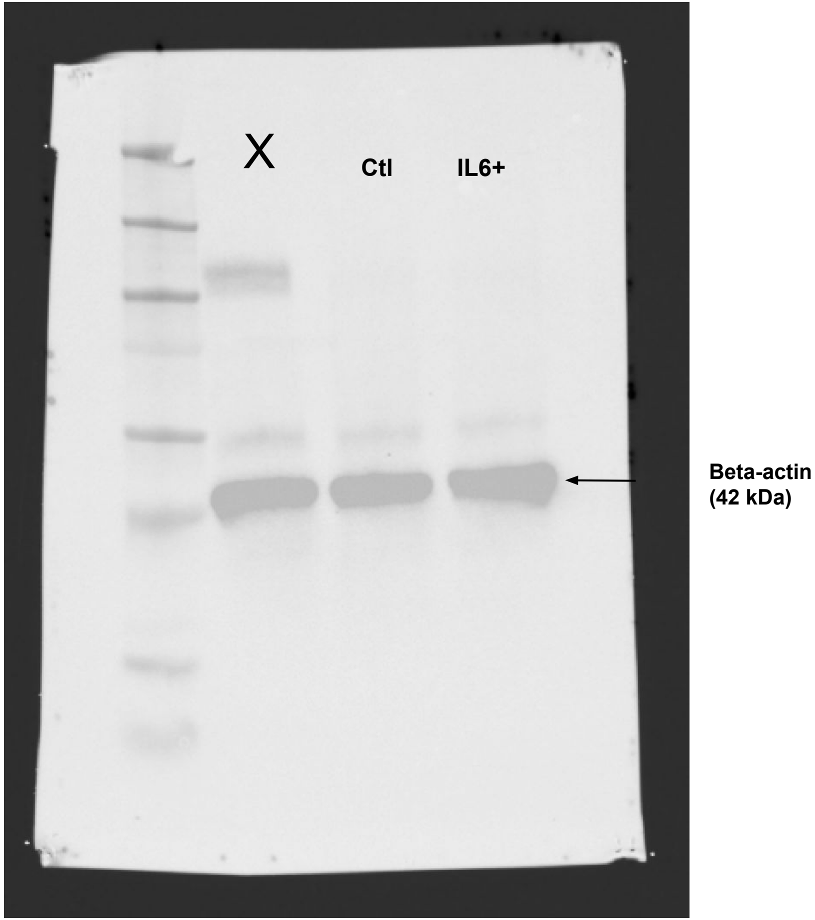

MCF10A cells_ROR1_replicate2

Figure 6

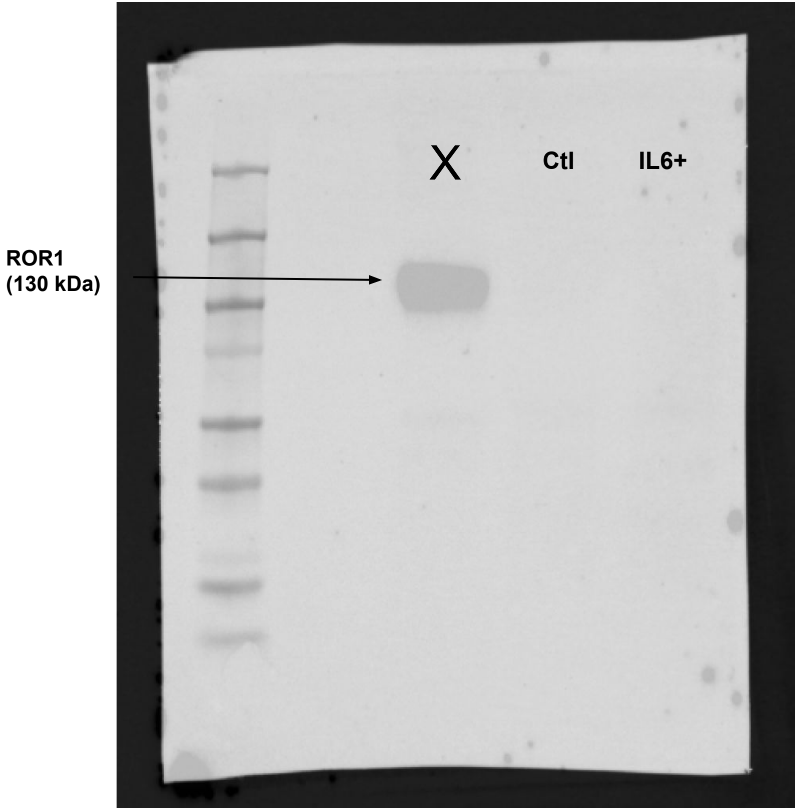

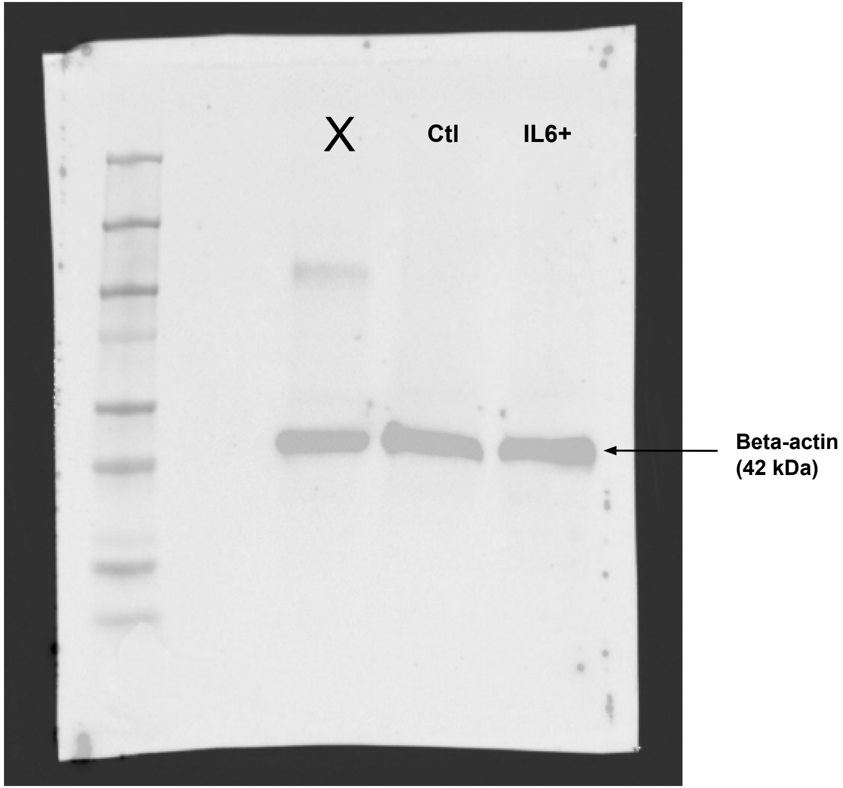

MCF10A cells_ROR1_replicate3

Figure 6

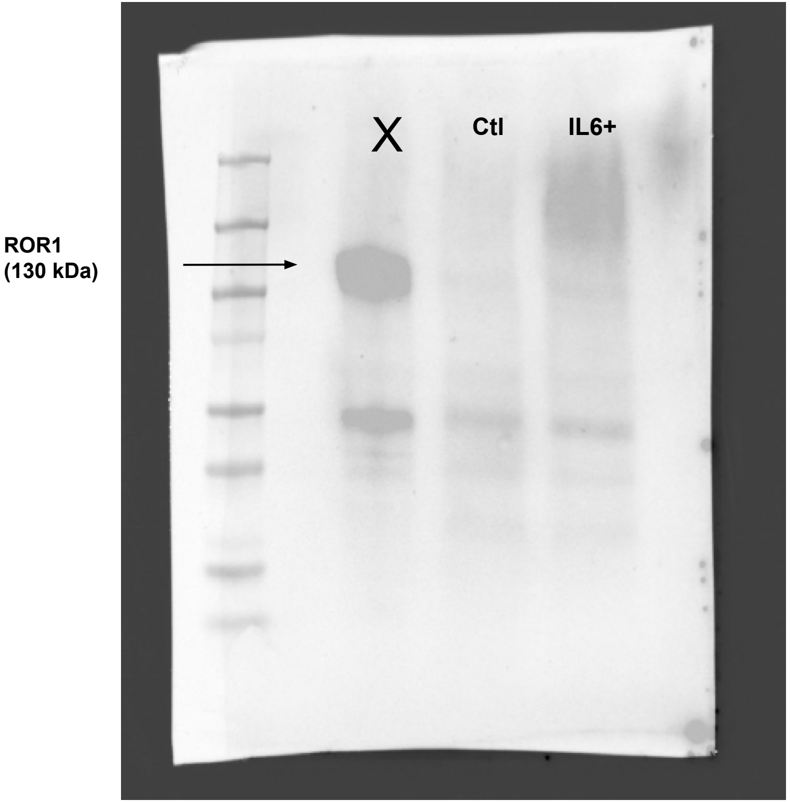

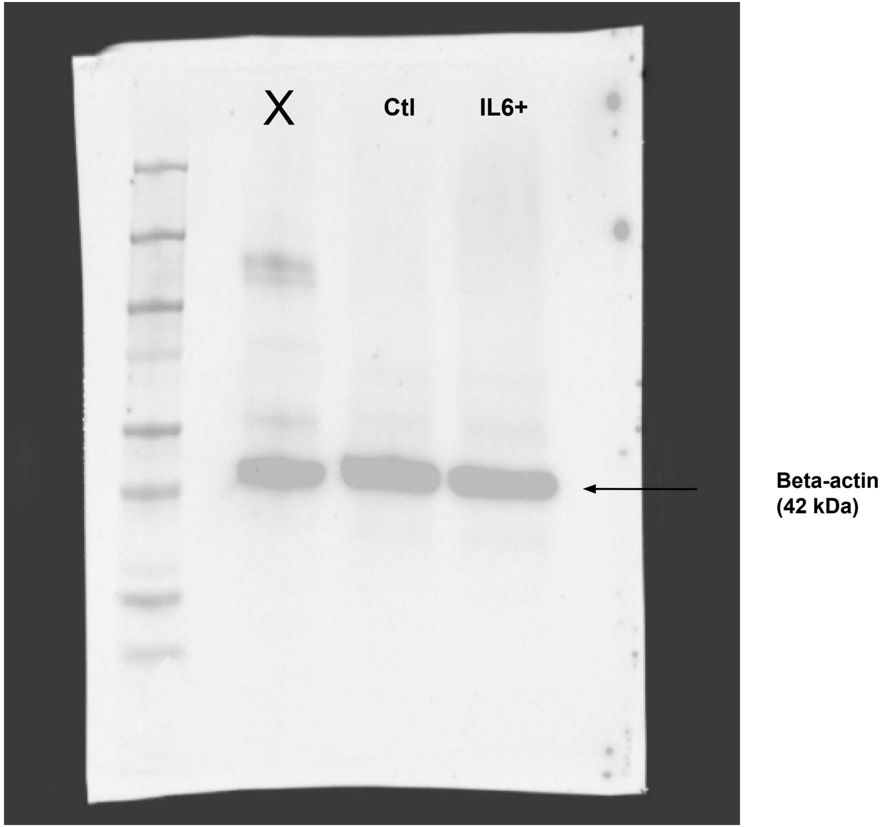

MCF10A cells_STAT3_replicate1

Figure 6

- Replicate 1 used in Figure 6a

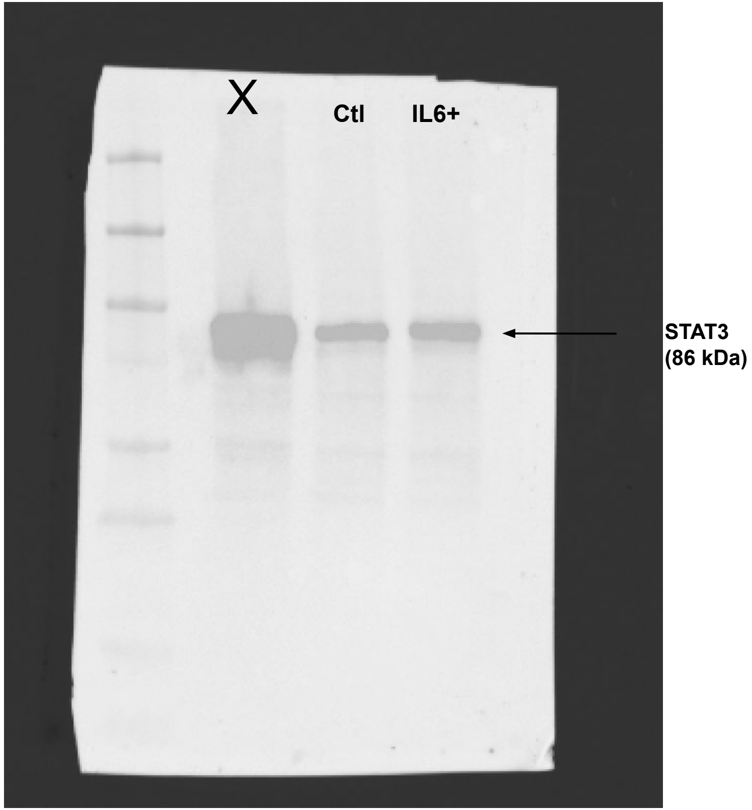

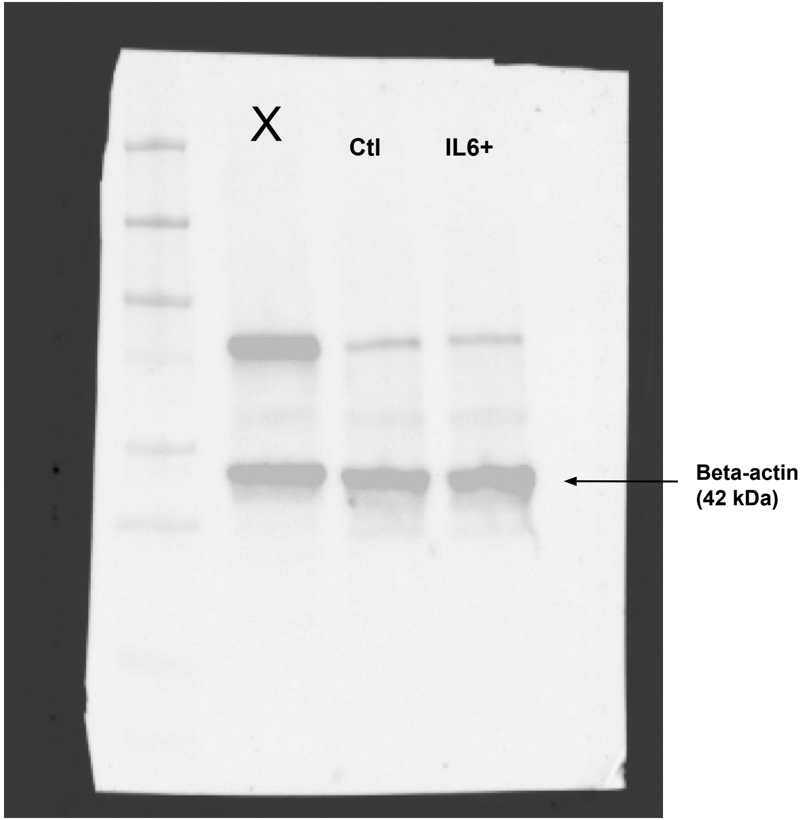

MCF10A cells_STAT3_replicate2

Figure 6

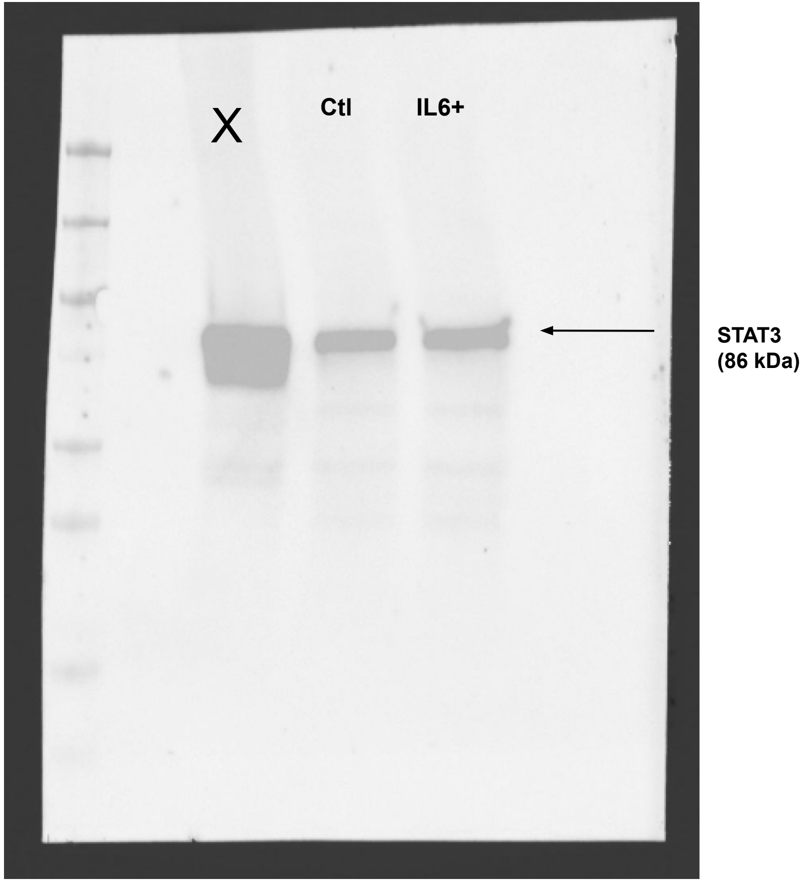

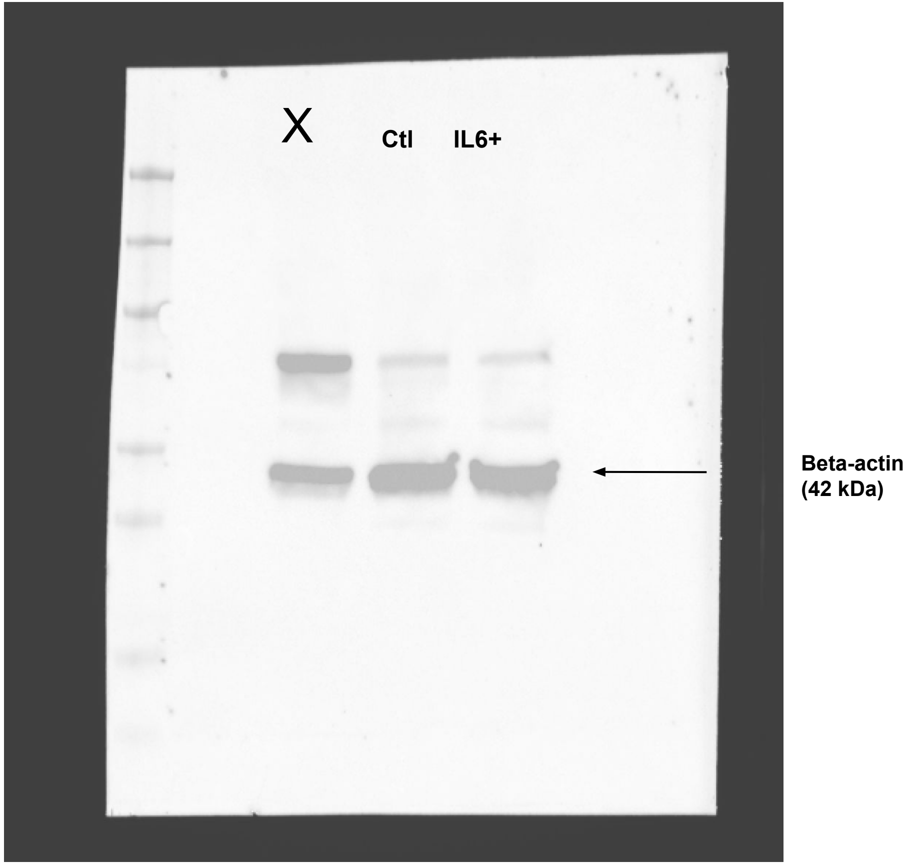

MCF10A cells_STAT3_replicate3 Figure 6

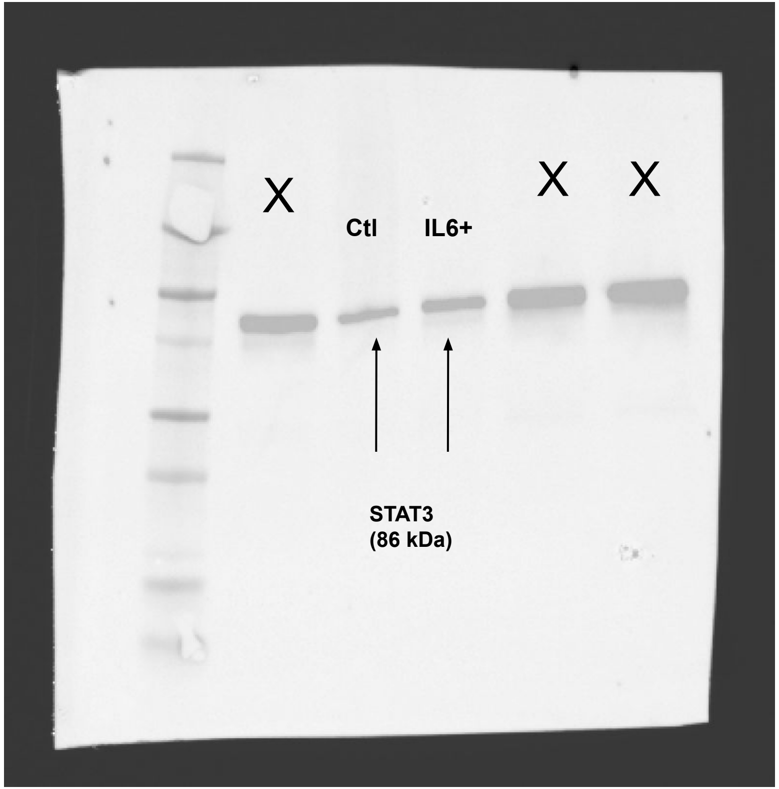

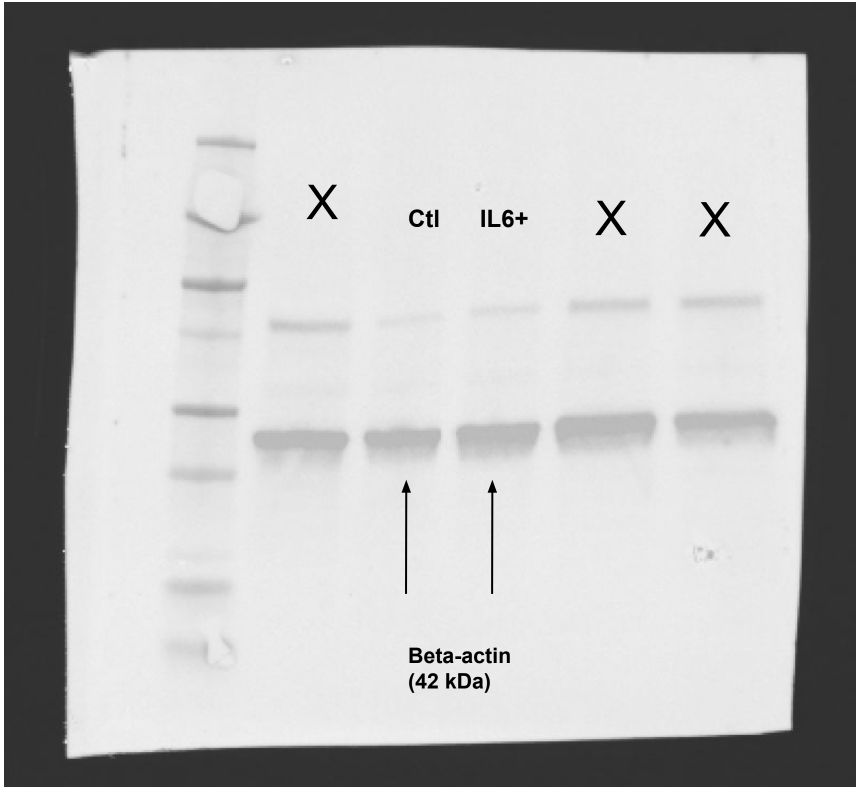

MCF10A cells_pSTAT3_replicate1 Figure 6

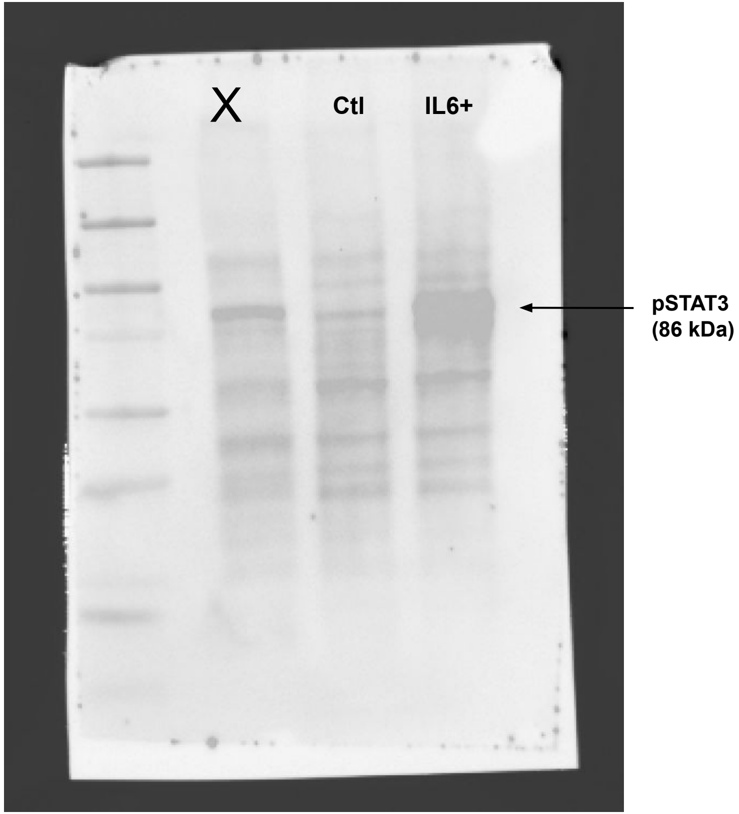

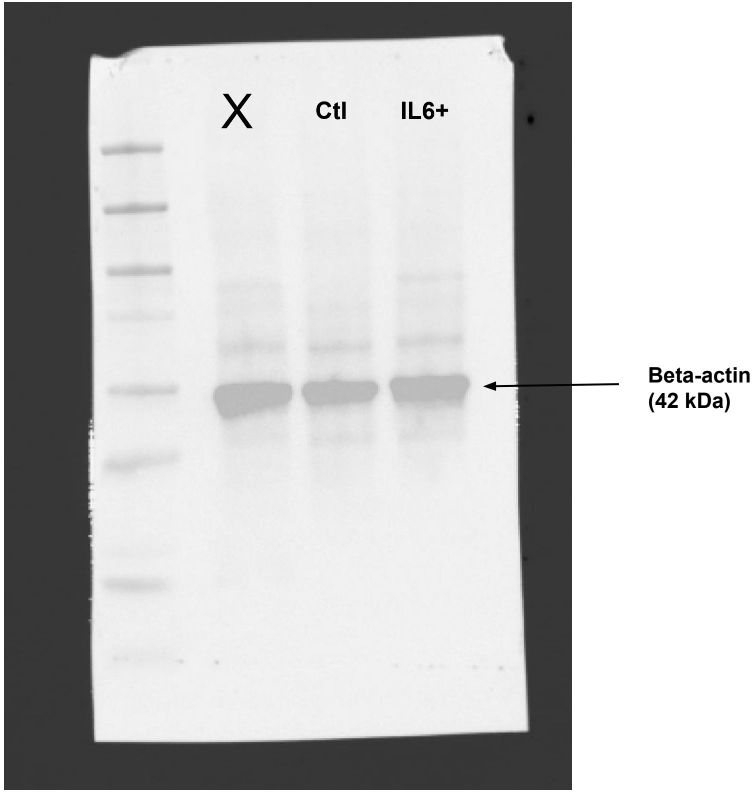

MCF10A cells_pSTAT3_replicate2 Figure 6

- Representative image used in Figure 6a

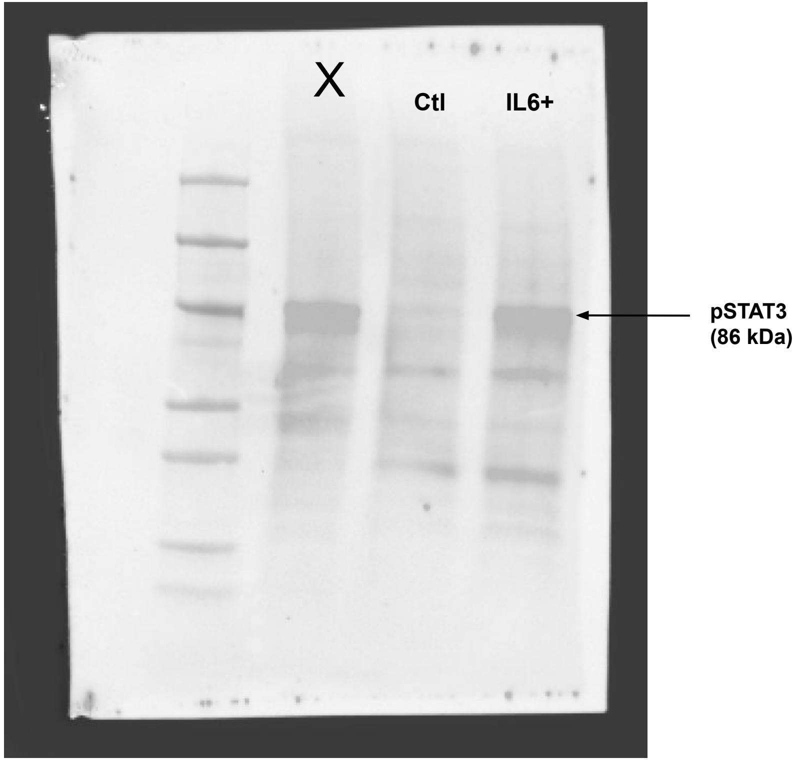

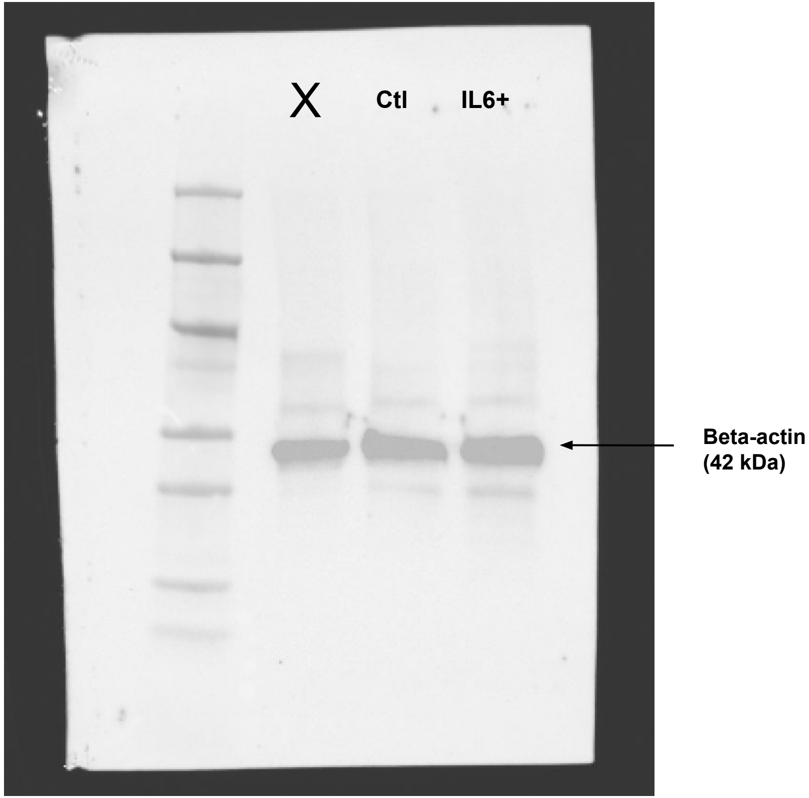

MCF10A cells_pSTAT3_replicate3 Figure 6

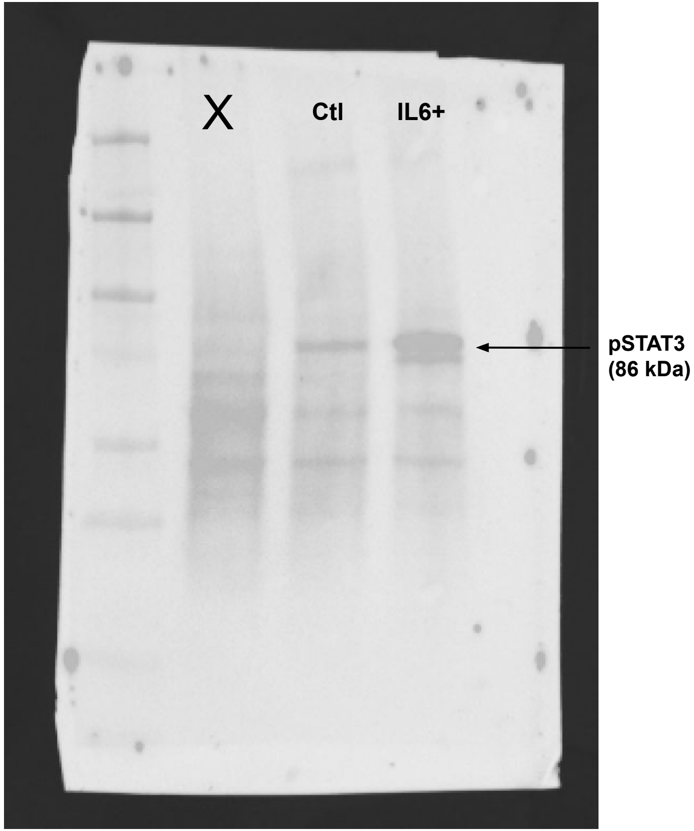

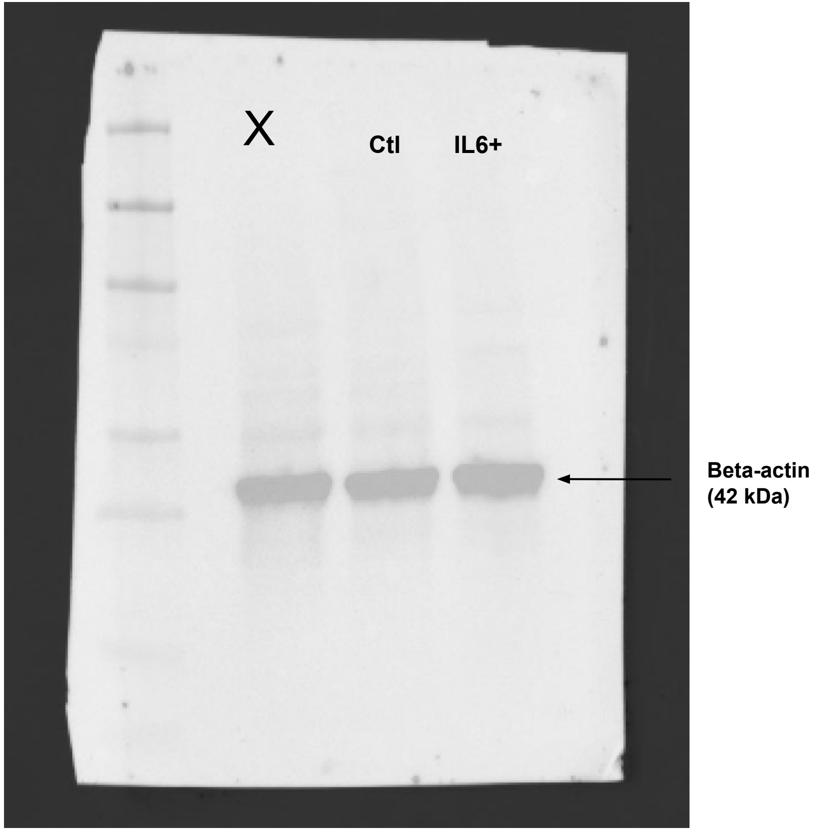

MCF10A cells_DNMT3A_replicate1

Figure 6

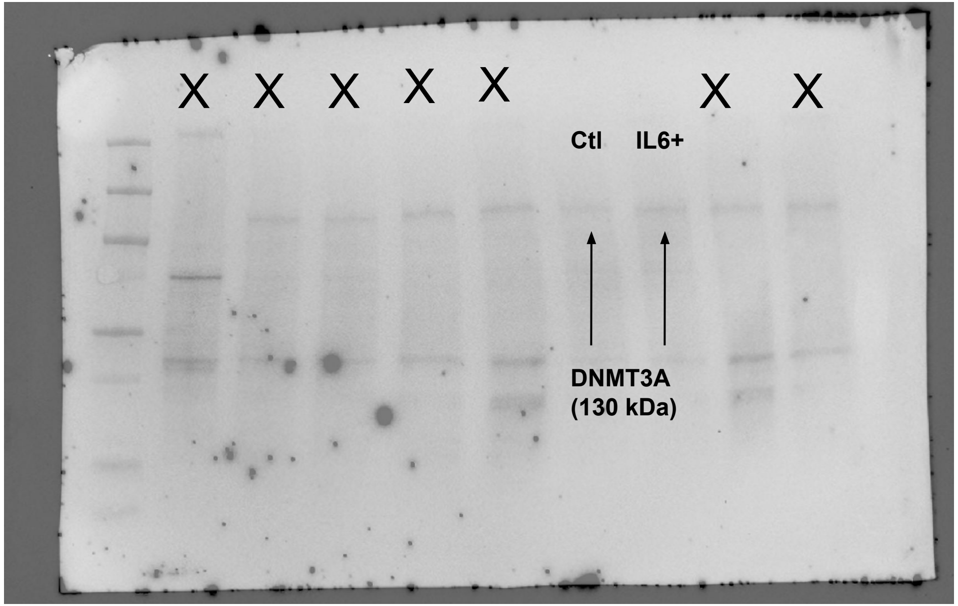

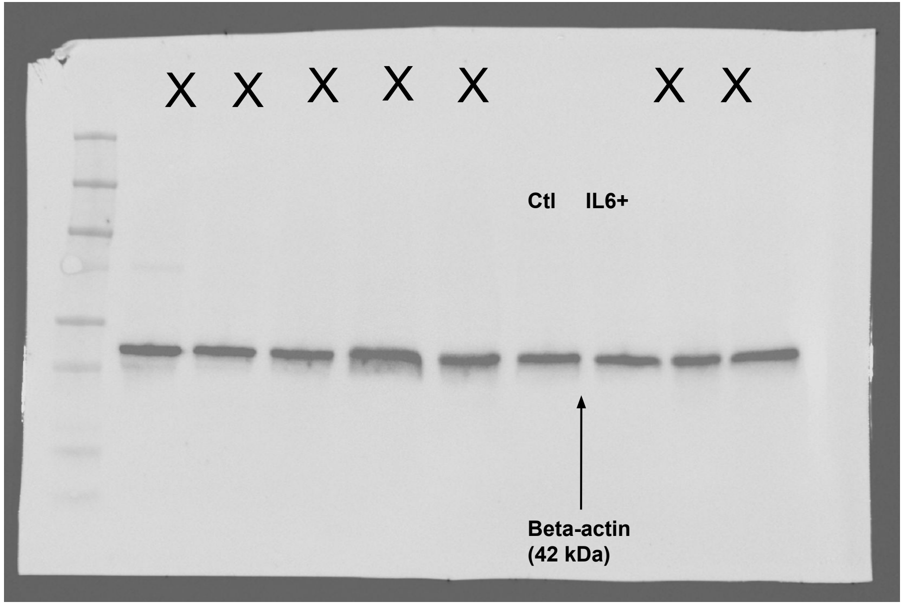

MCF10A cells_DNMT3A_replicate2

Figure 6a

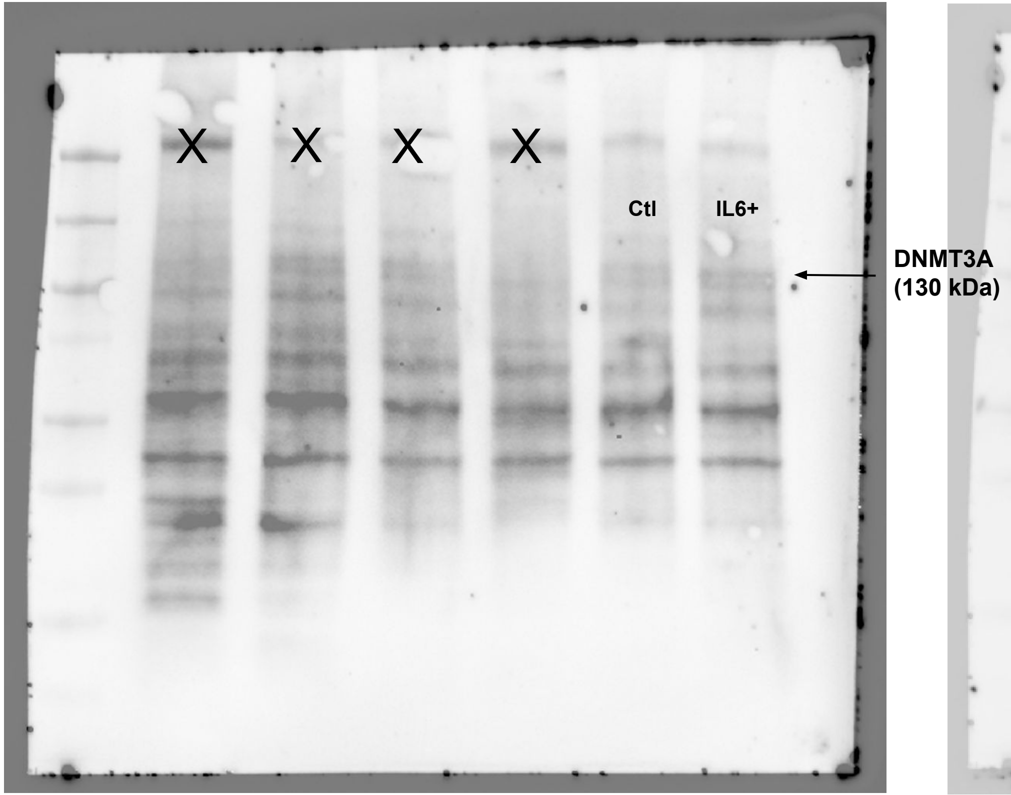

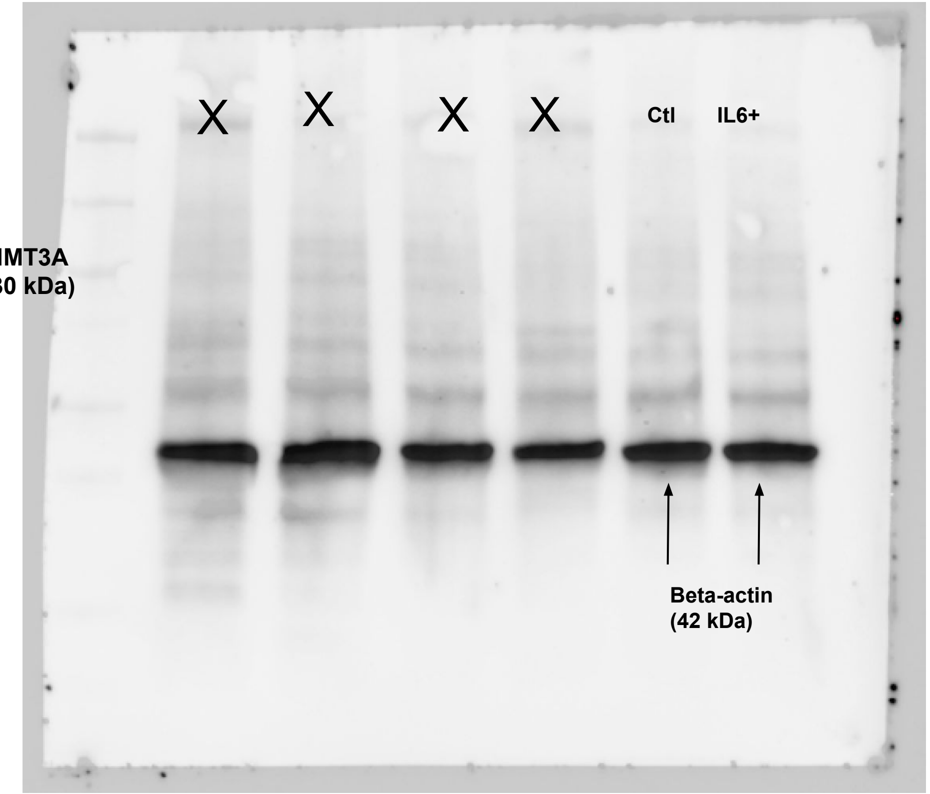

MCF10A cells_DNMT3A_replicate3

Figure 6

- Representative image figure 6a

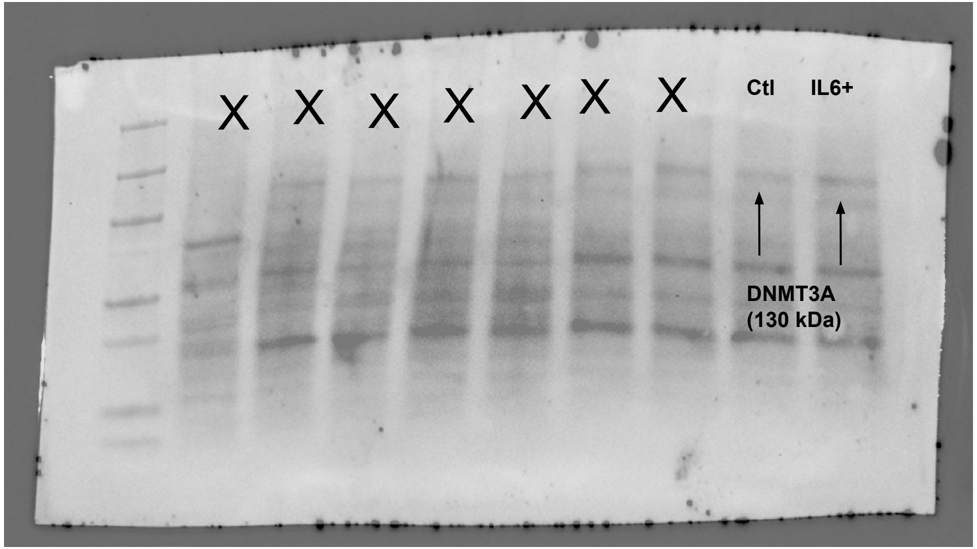

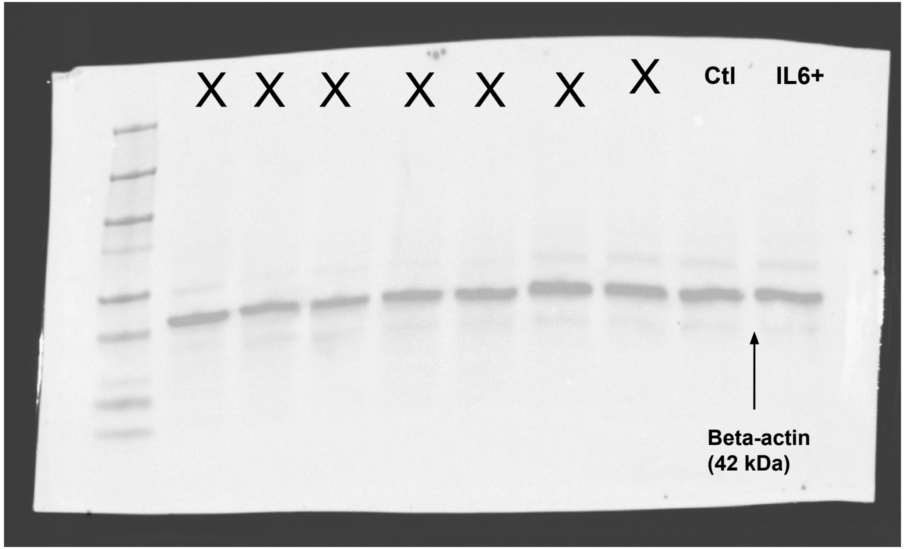

MCF10A cells_DNMT3B_replicate1

Figure 6

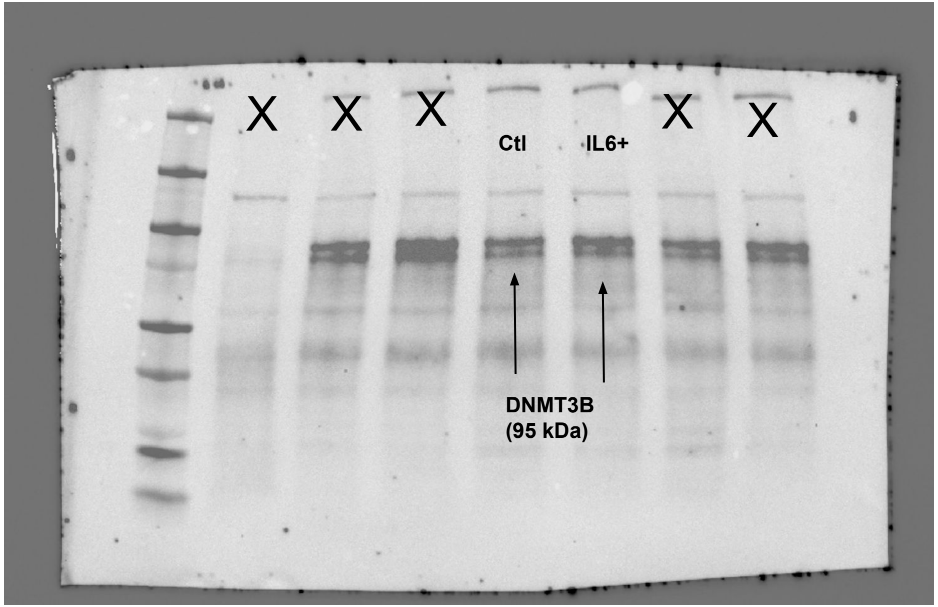

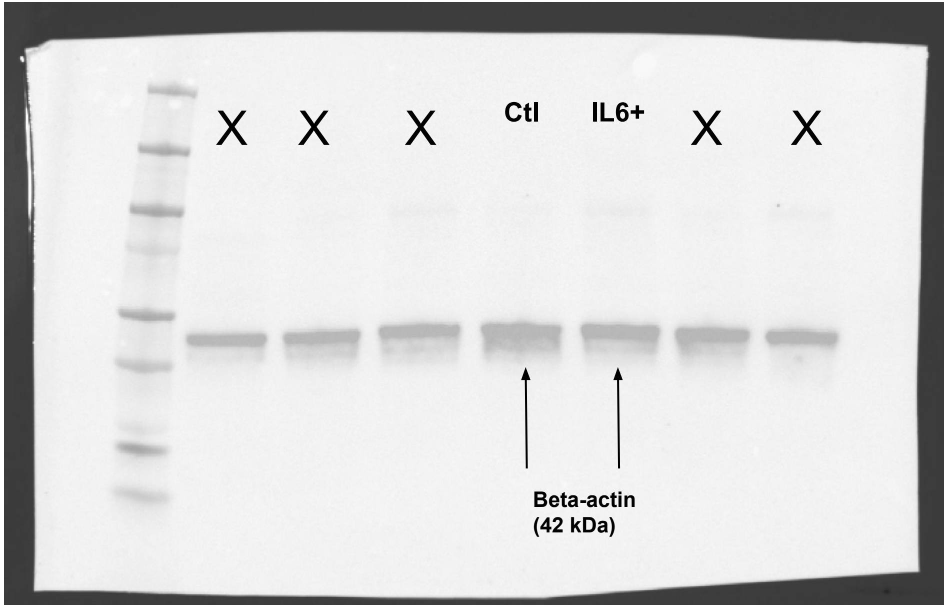

MCF10A cells_DNMT3B_replicates2&3 Figure 6

- Replicate 3 was used as a representative image in Figure 6a

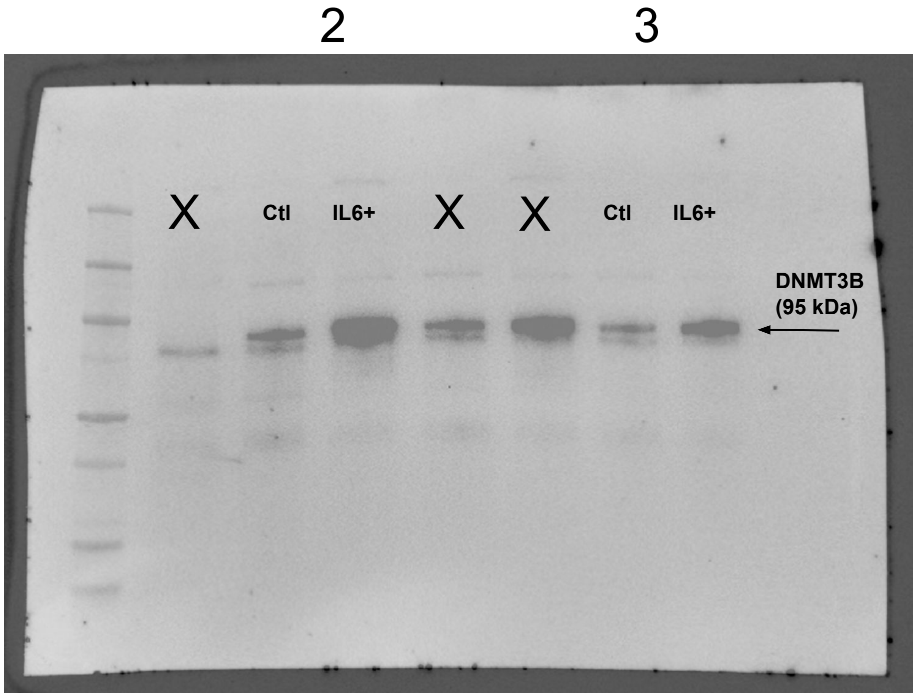

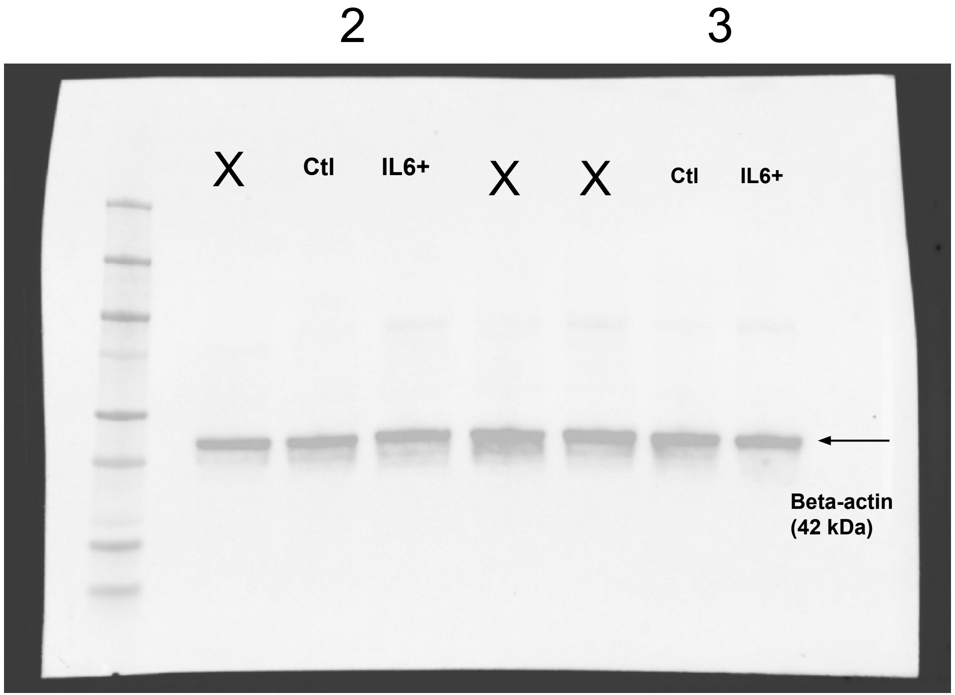

MCF10A cells_CREB3L1_replicate1

Figure 6

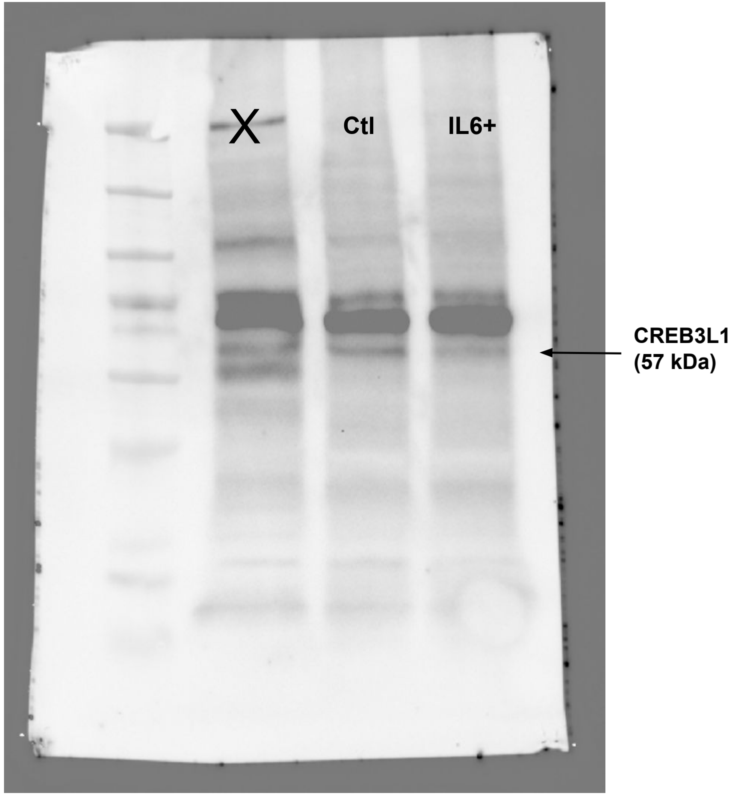

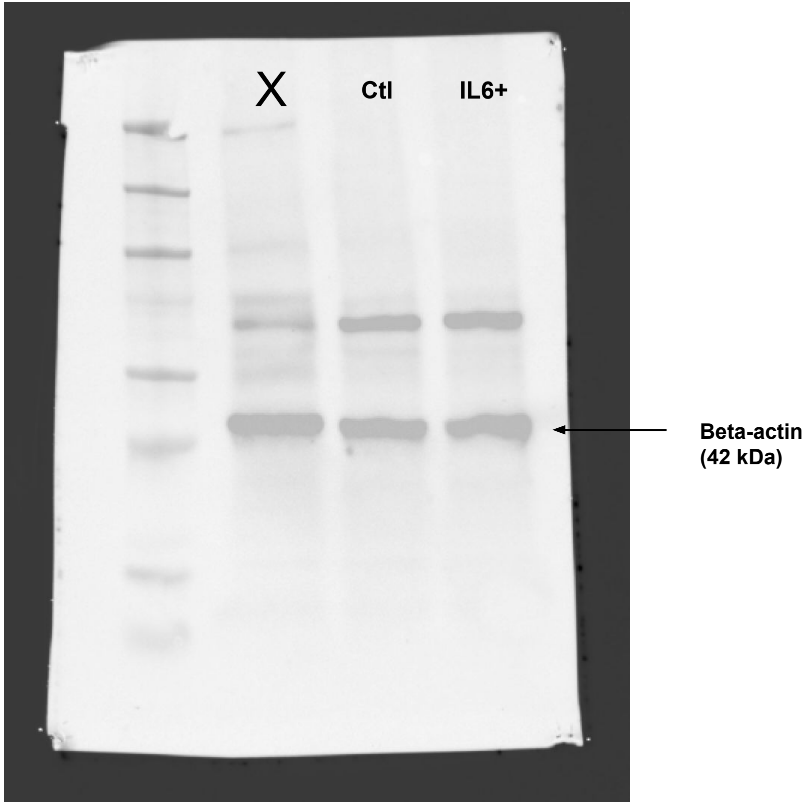

MCF10A cells_CREB3L1_replicates2&3 Figure 6

- Replicate 2 is a representative image in Figure 6a

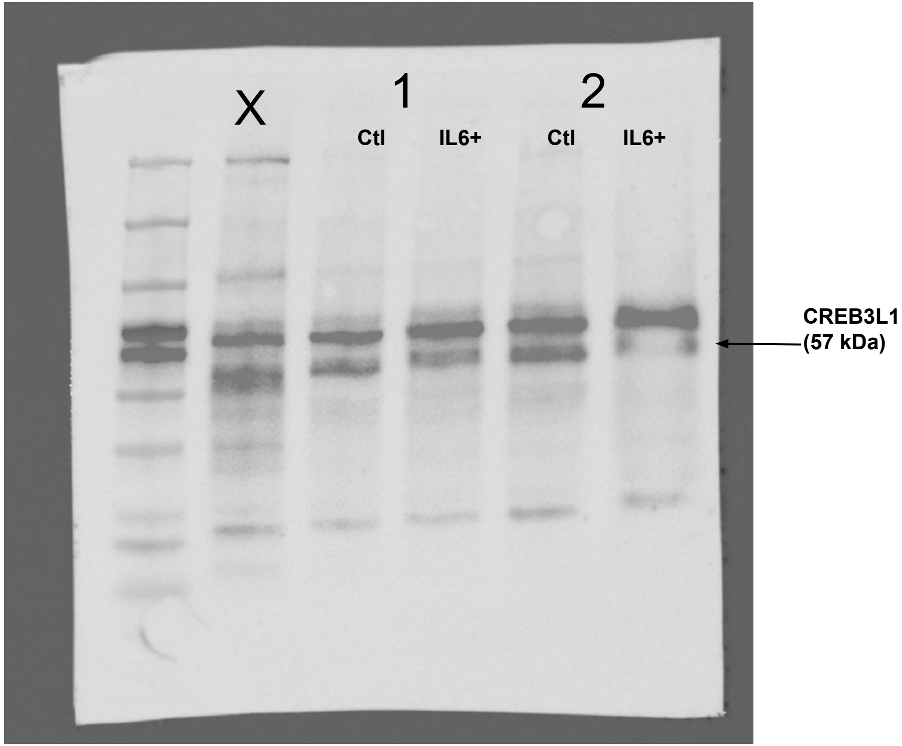

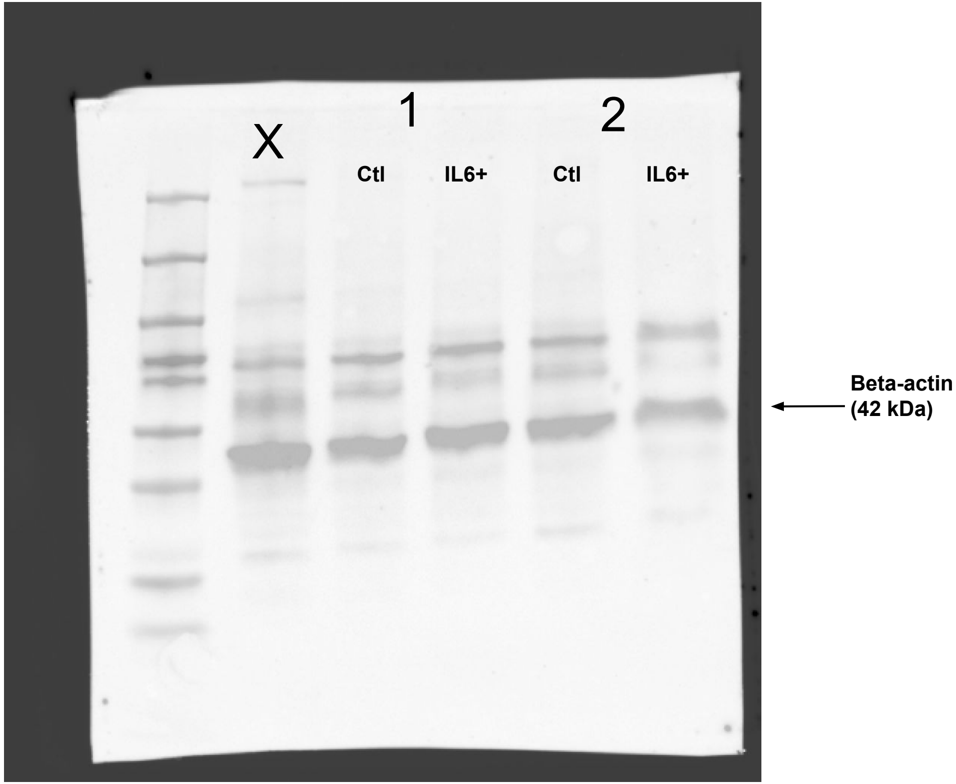

Raw western blot data

1. All lanes used in the manuscript and used for densitometry analysis are labeled. Unlabeled lanes were not used for analysis.
2. All proteins used in the manuscript and for analysis are labeled “ctl” or “si”. Lanes labeled “X” were not utilized.
3. Protein lysates were obtained from HCC1806 and MDA-MB-231 cell lines.
4. Lanes labeled “si” are proteins from cells lines that were treated with siRNA to induce STAT3 knockdown. Lanes that are labeled “Ctl” are the control samples treated with scrambled RNA.
5. All densitometry analysis was normalized to a housekeeping gene, beta-actin.
6. Brightness and contrast of blots may have been adjusted to make the protein bands more visible in the manuscript.

HCC1806 cells_STAT3_replicate1 Figure 6

- Representative image in figure 6c

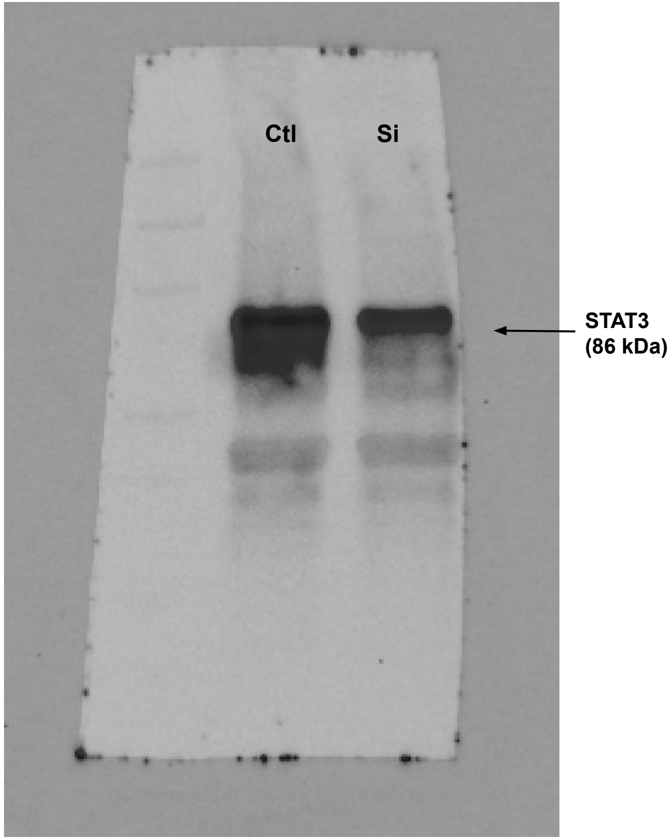

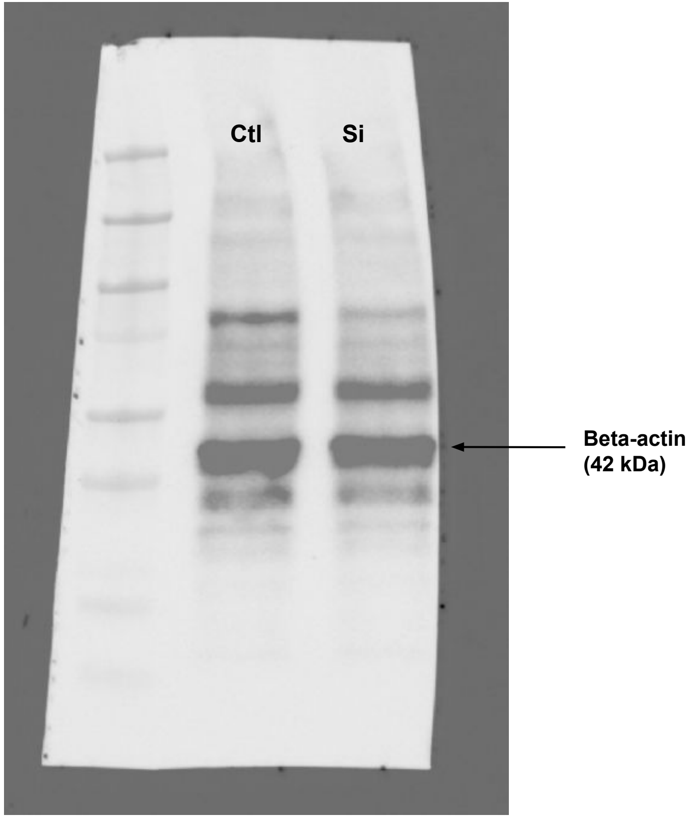

HCC1806 cells_STAT3_replicate2

Figure 6

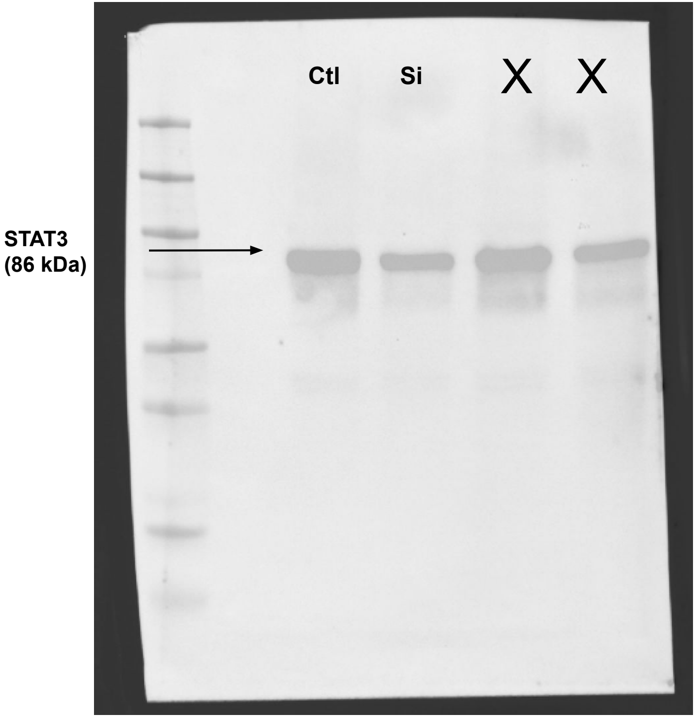

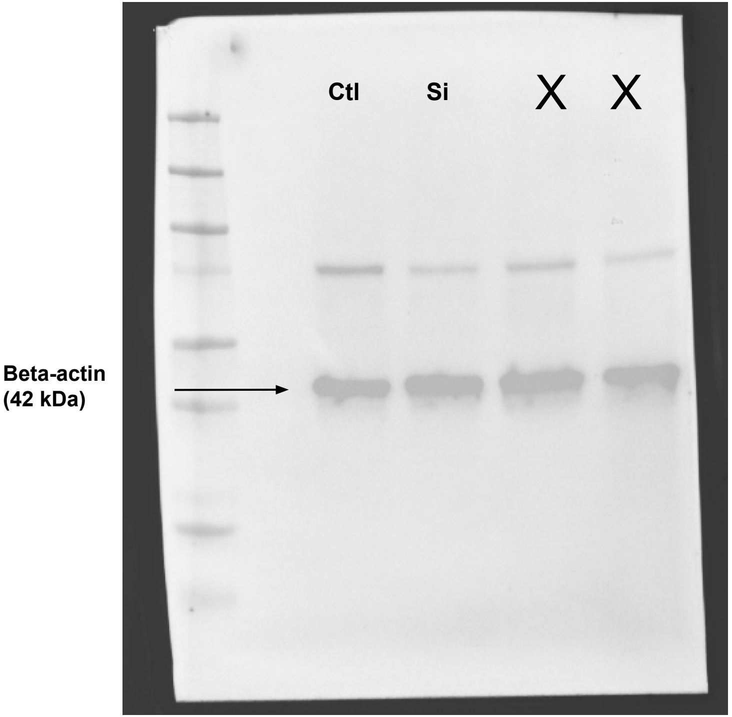

HCC1806 cells_STAT3_replicate3

- Replicate 3 was used in Figure __

STAT3
(86 kDa)

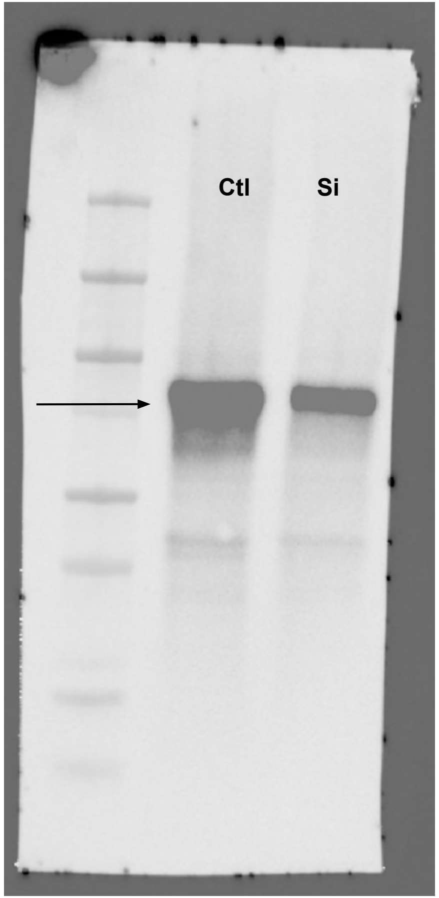

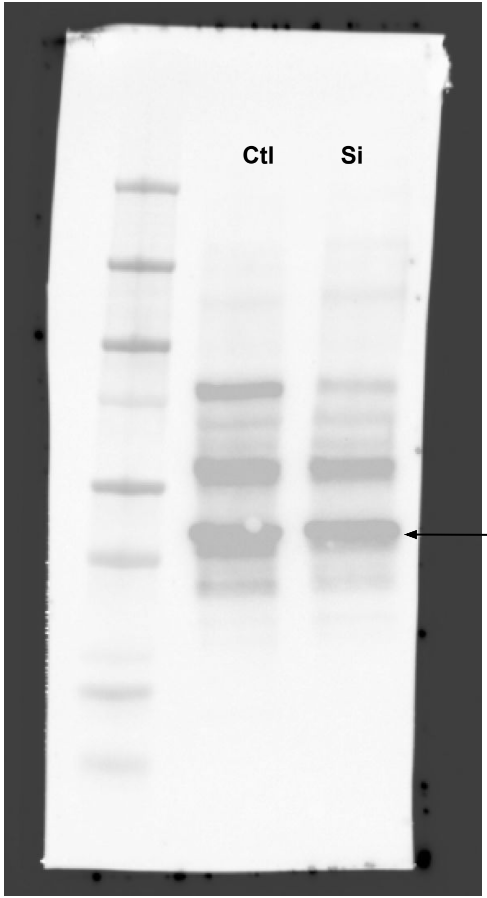

Beta-actin
(42 kDa)

HCC1806 cells_pSTAT3_replicate1 Figure 6c

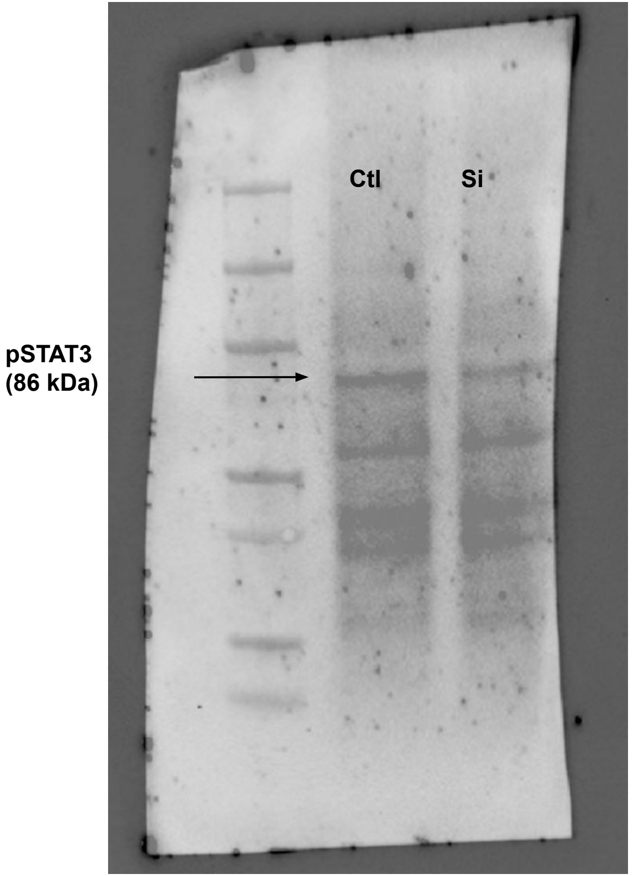

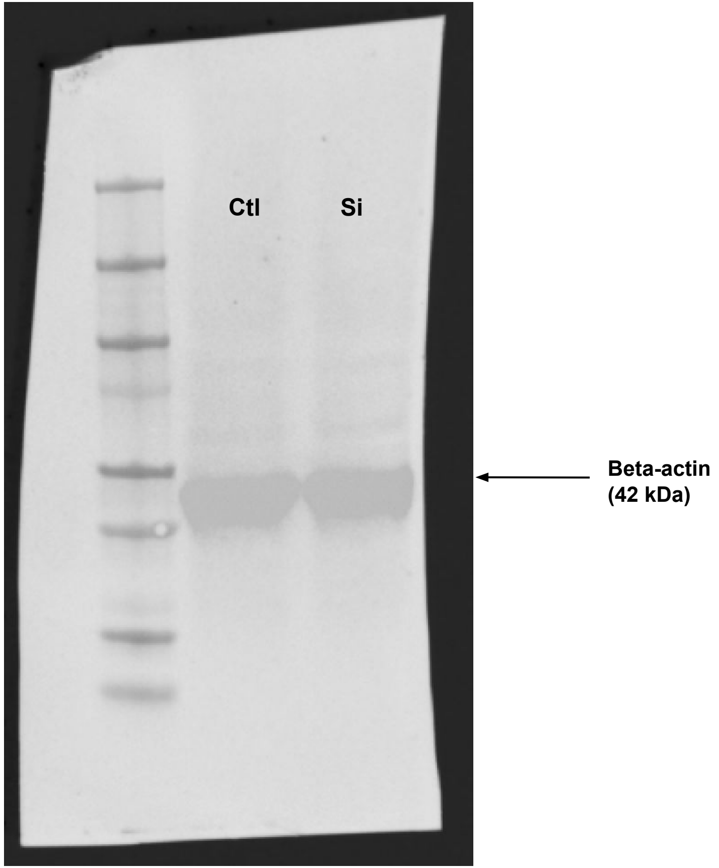

HCC1806 cells_pSTAT3_replicate2

Figure 6

- Representative image Figure 6c

pSTAT3
(86 kDa)

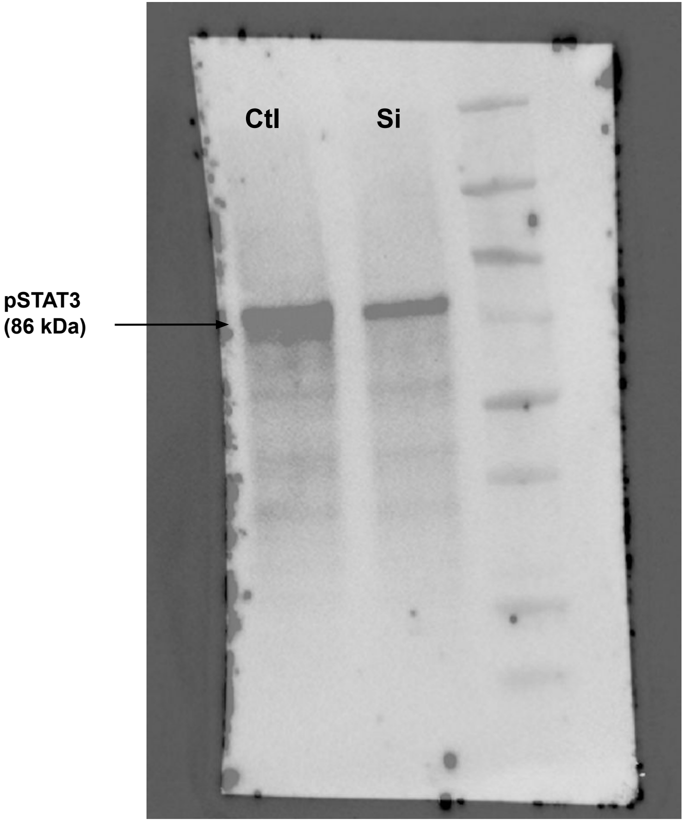

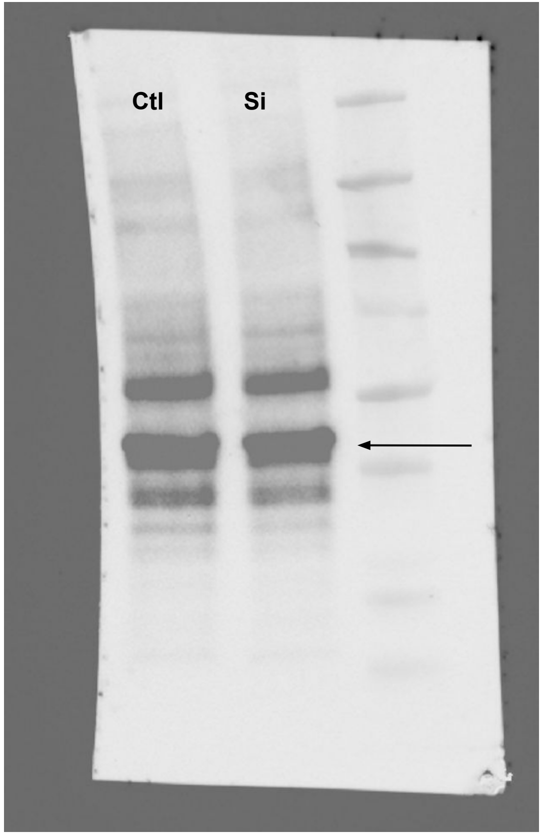

Beta-actin
(42 kDa)

HCC1806 cells_pSTAT3_replicate3 Figure 6

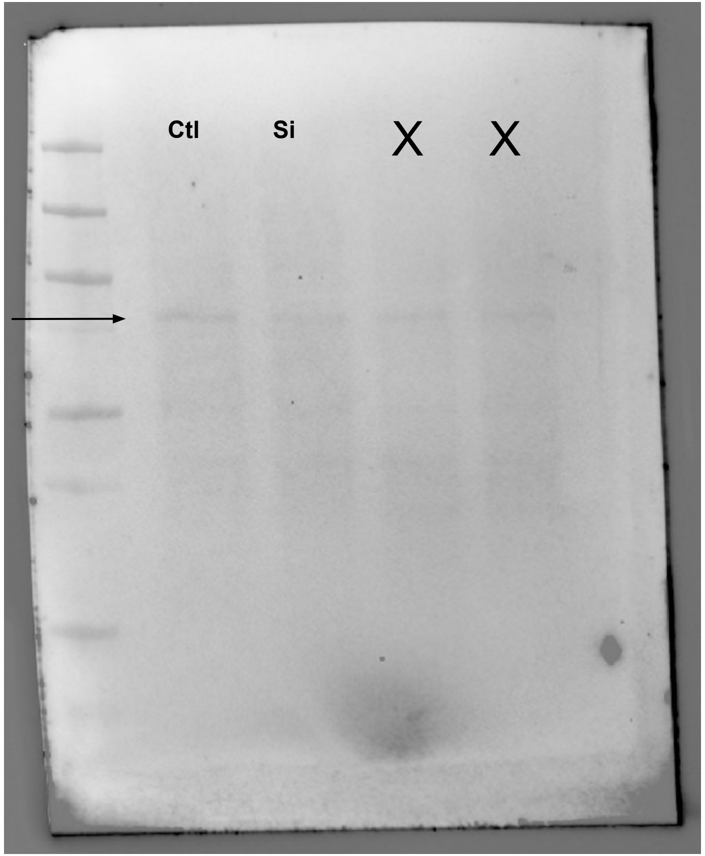

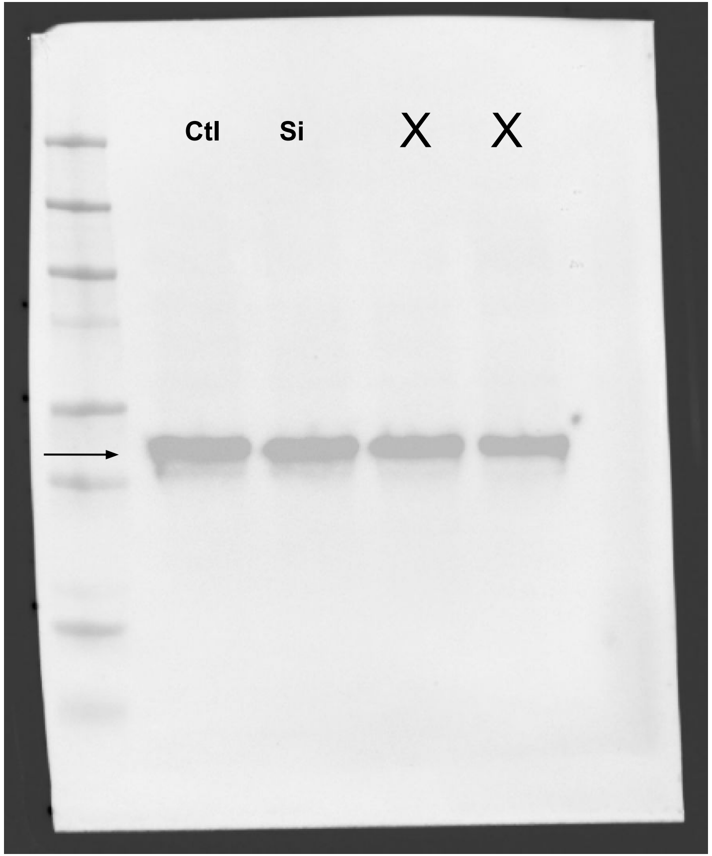

HCC1806 cells_ROR1_replicate1 Figure 6

ROR1
(130 kDa)

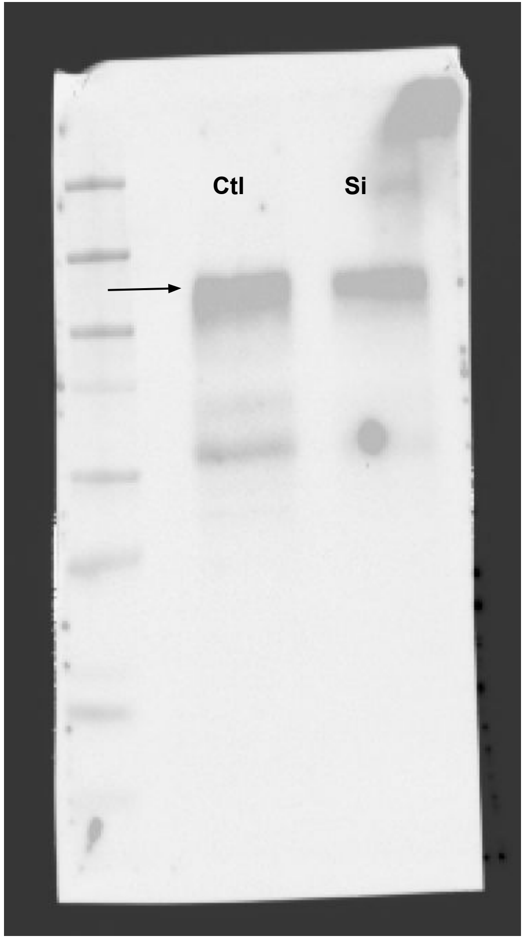

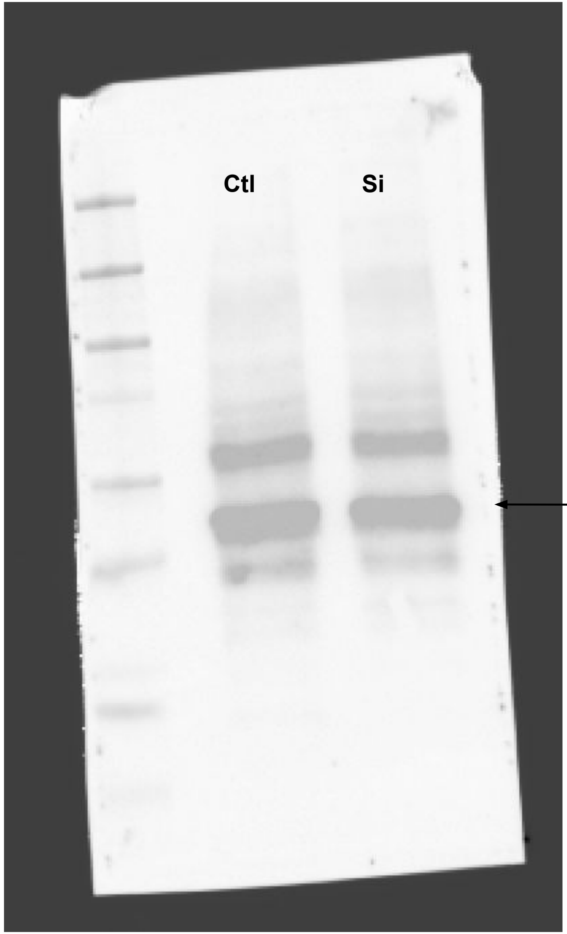

Beta-actin
(42 kDa)

HCC1806 cells_ROR1_replicate2 Figure 6

- Representative image figure 6c

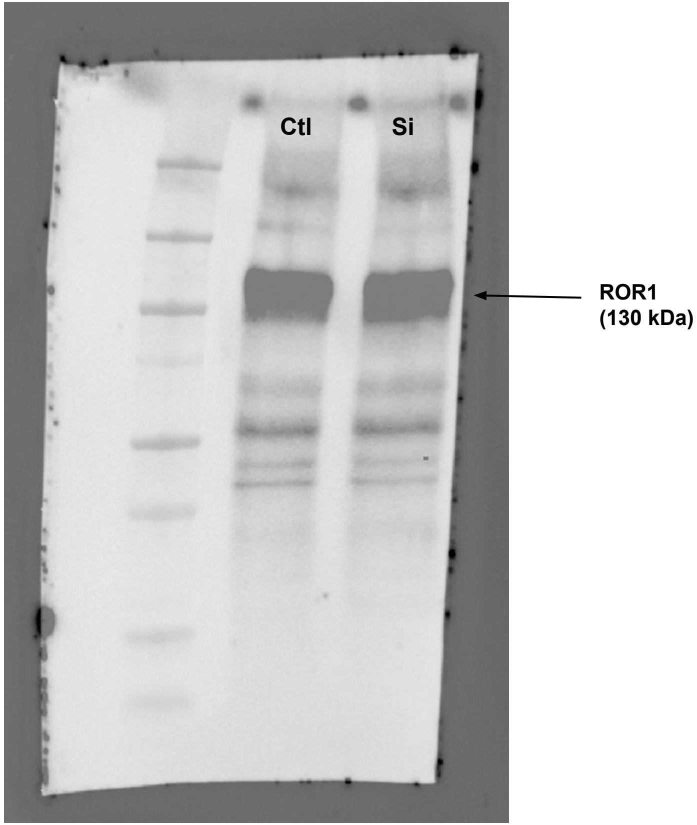

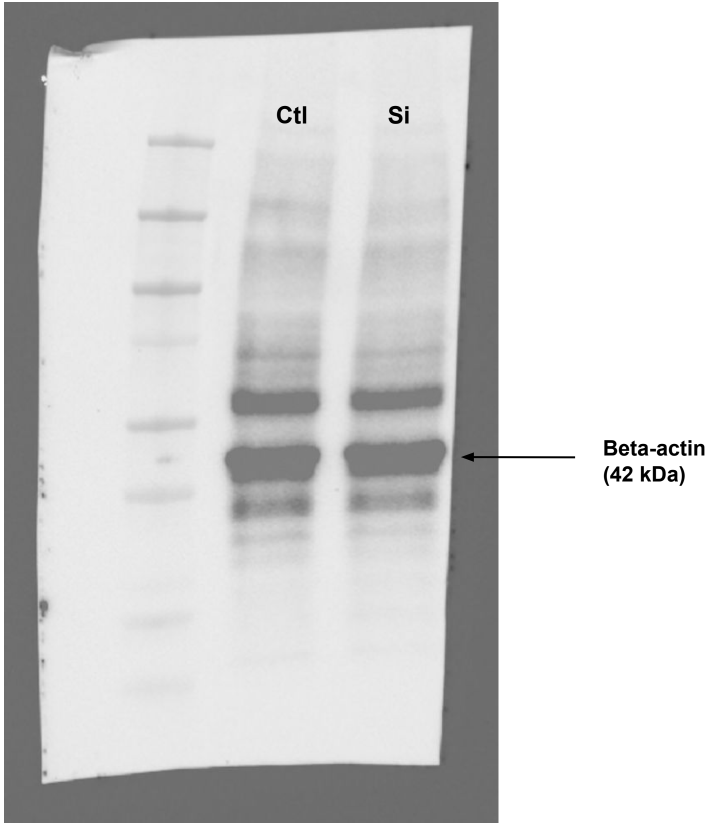

HCC1806 cells_ROR1_replicate3

Figure 6

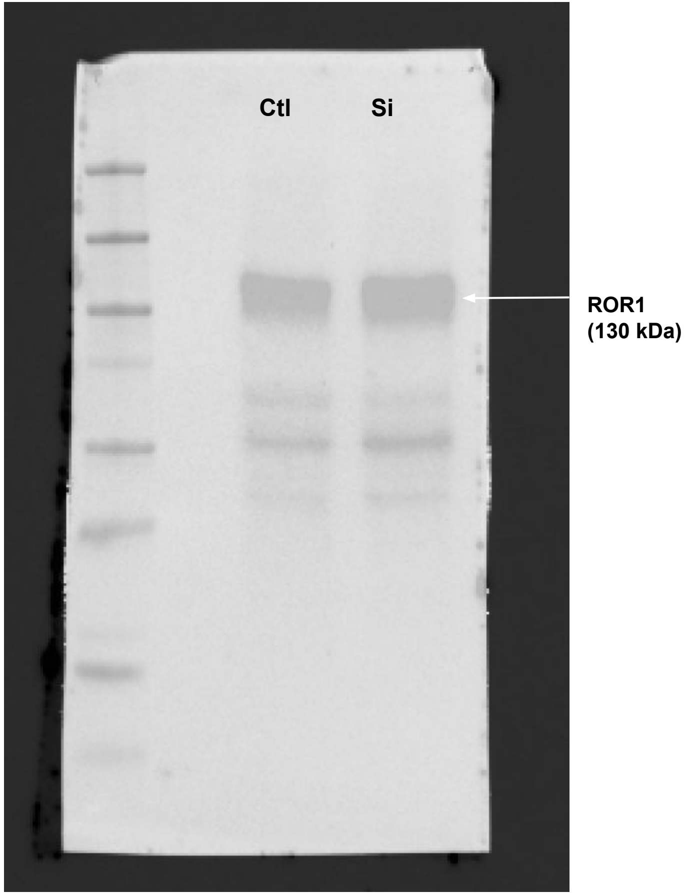

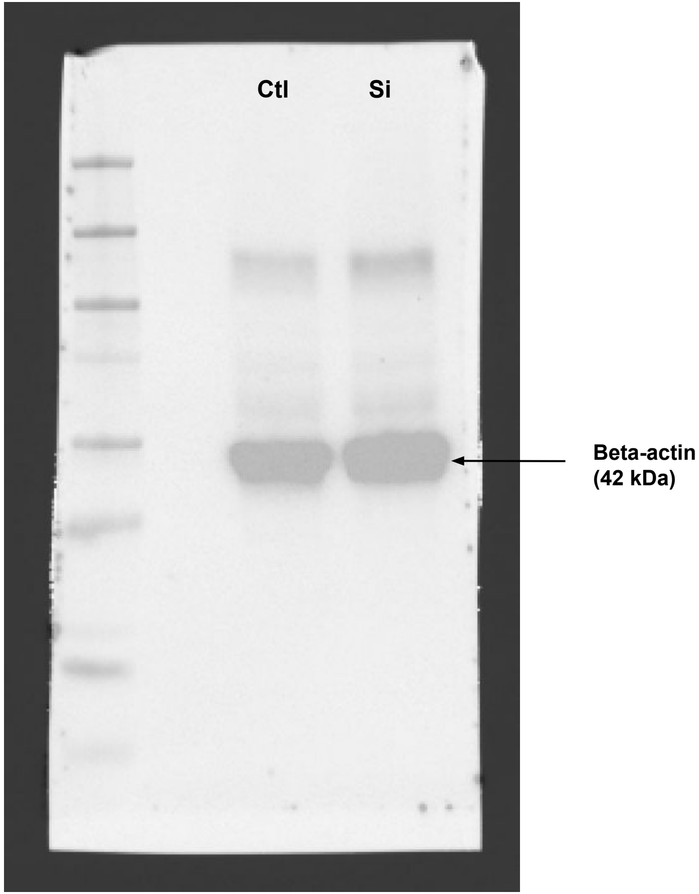

HCC1806 cells_CREB3L1_replicate1 Figure 6

- Representative image used in Figure 6c

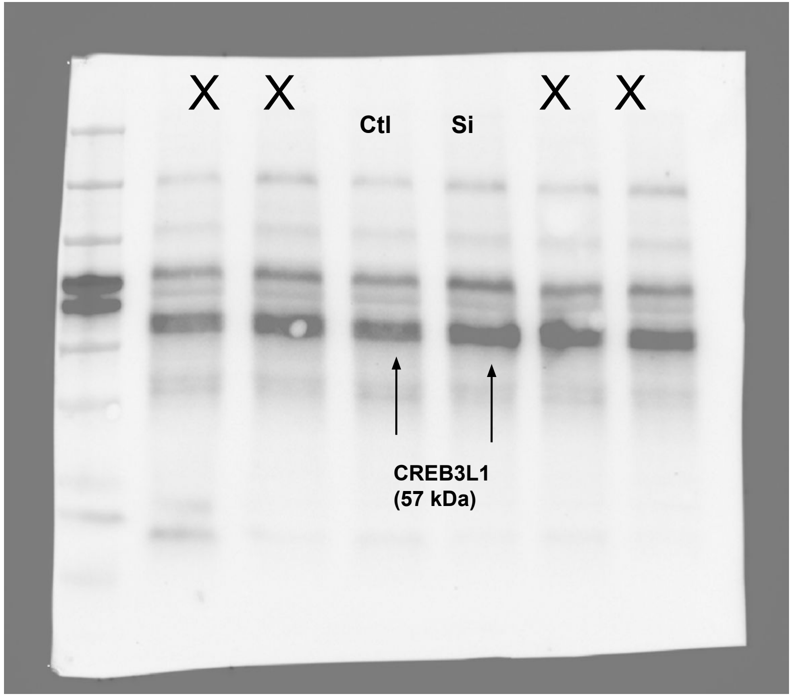

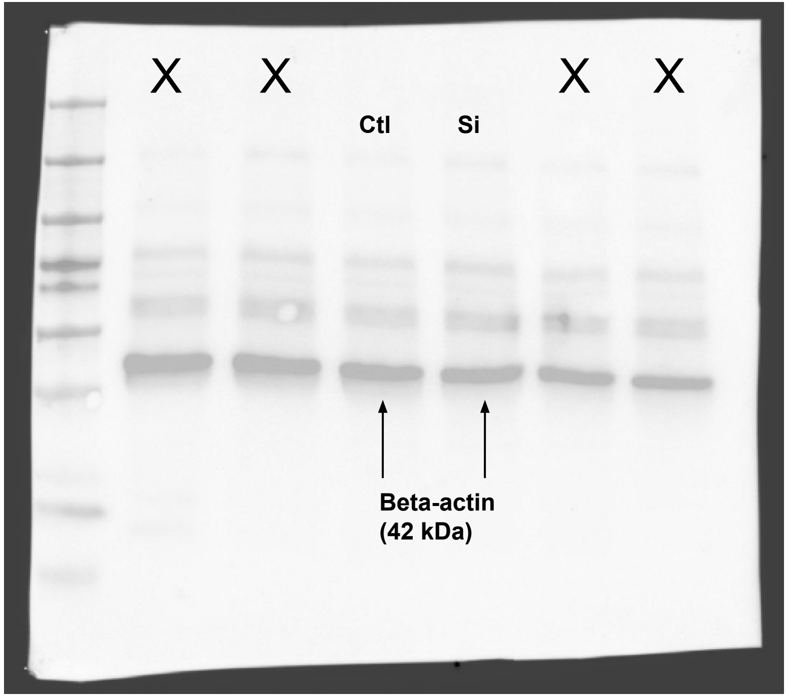

HCC1806 cells_CREB3L1_replicate2

Figure 6

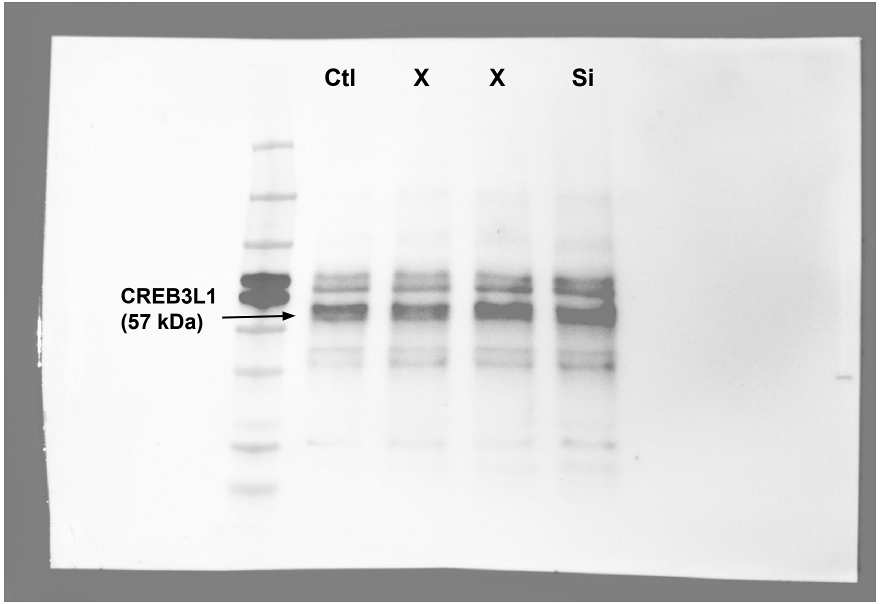

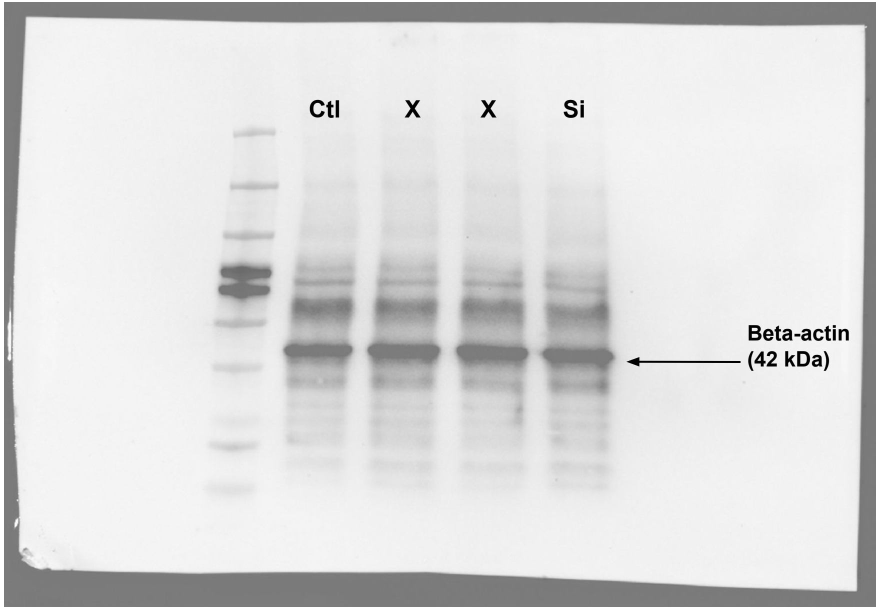

HCC1806 cells_CREB3L1_replicate3

Figure 6

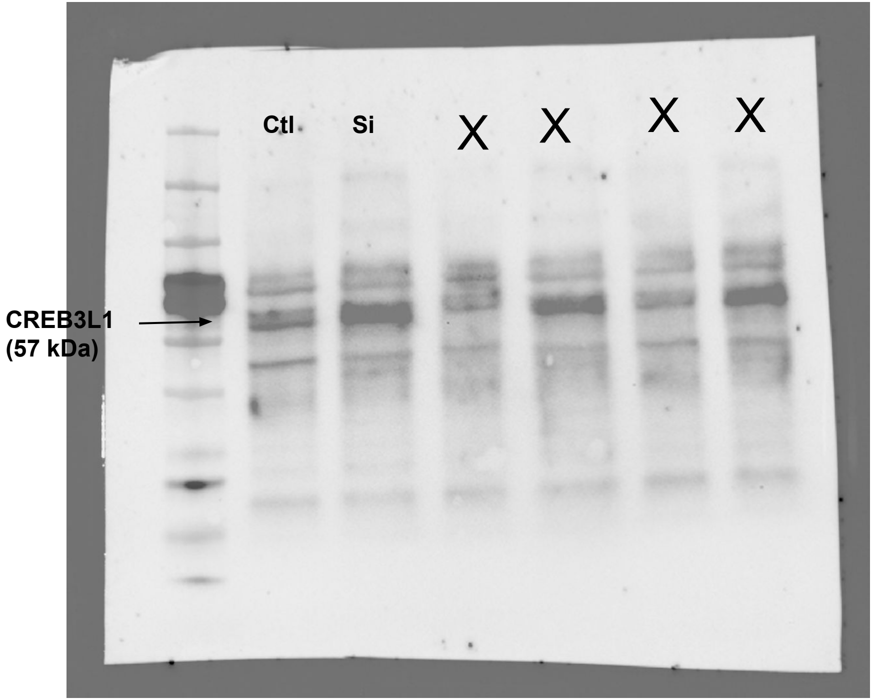

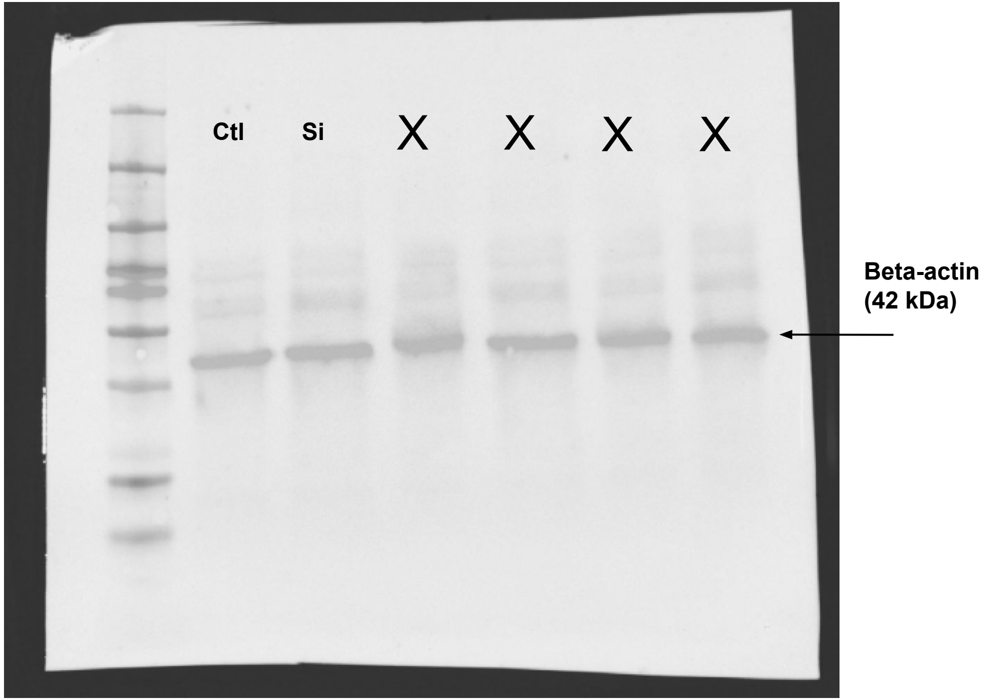

MDA-MB-231 cells_STAT3_replicate1

Figure 6

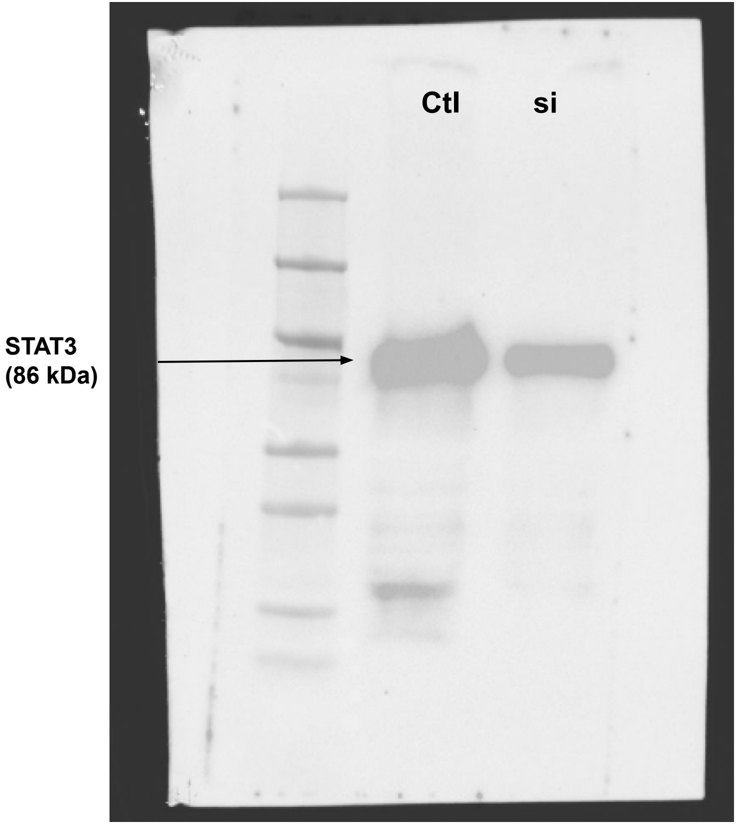

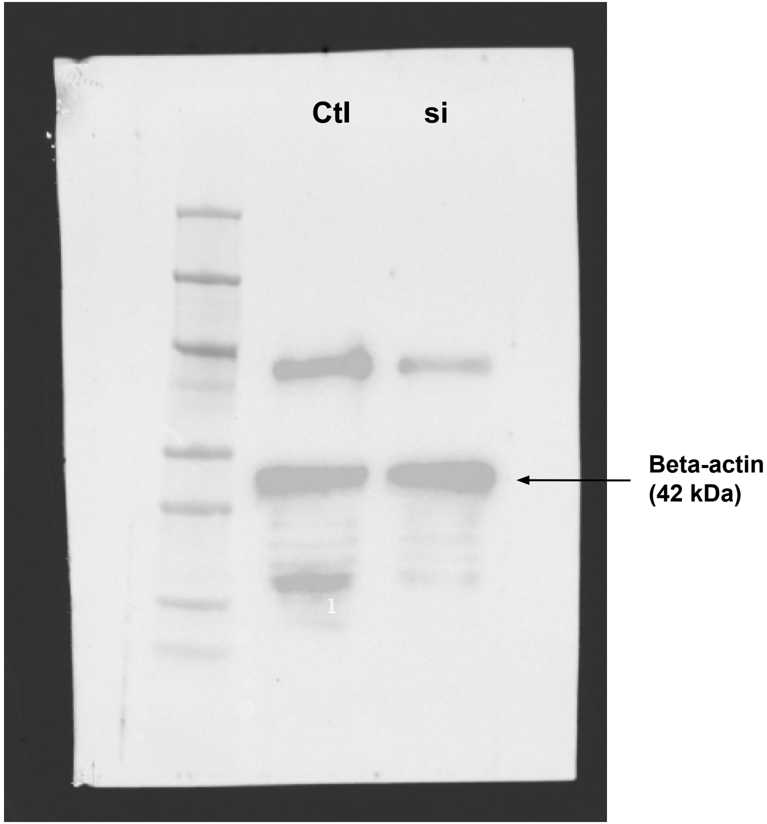

MDA-MB-231 cells_STAT3_replicate2 and 3 Figure 6

- Representative image 3 used in Figure 6a

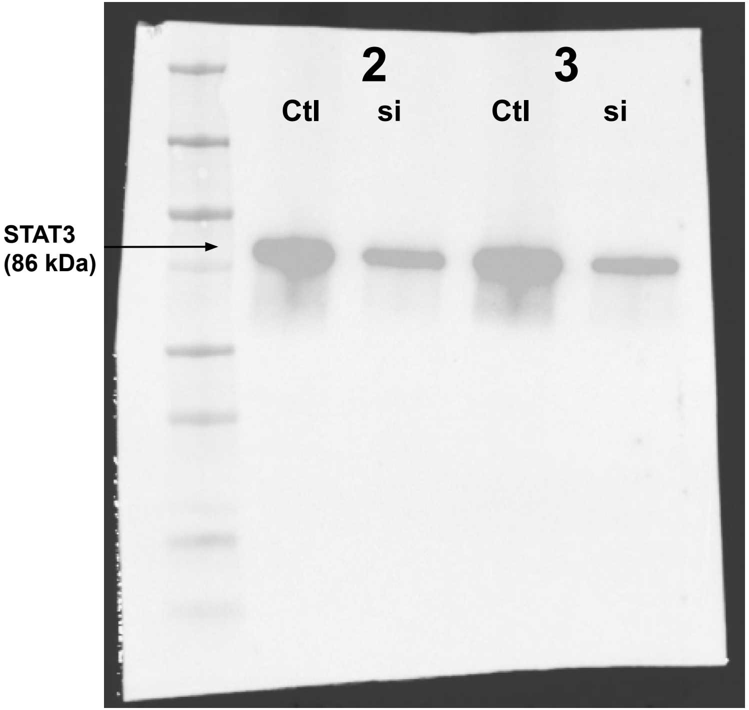

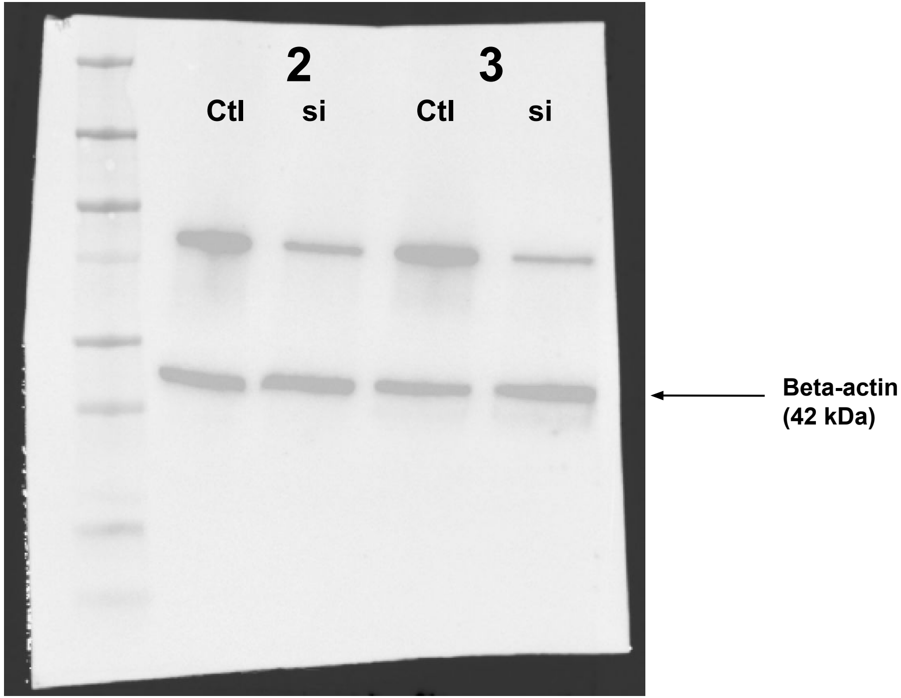

MDA-MB-231 cells_pSTAT3_replicates1+2

Figure 6

- Replicate 1 was used in Figure 6a

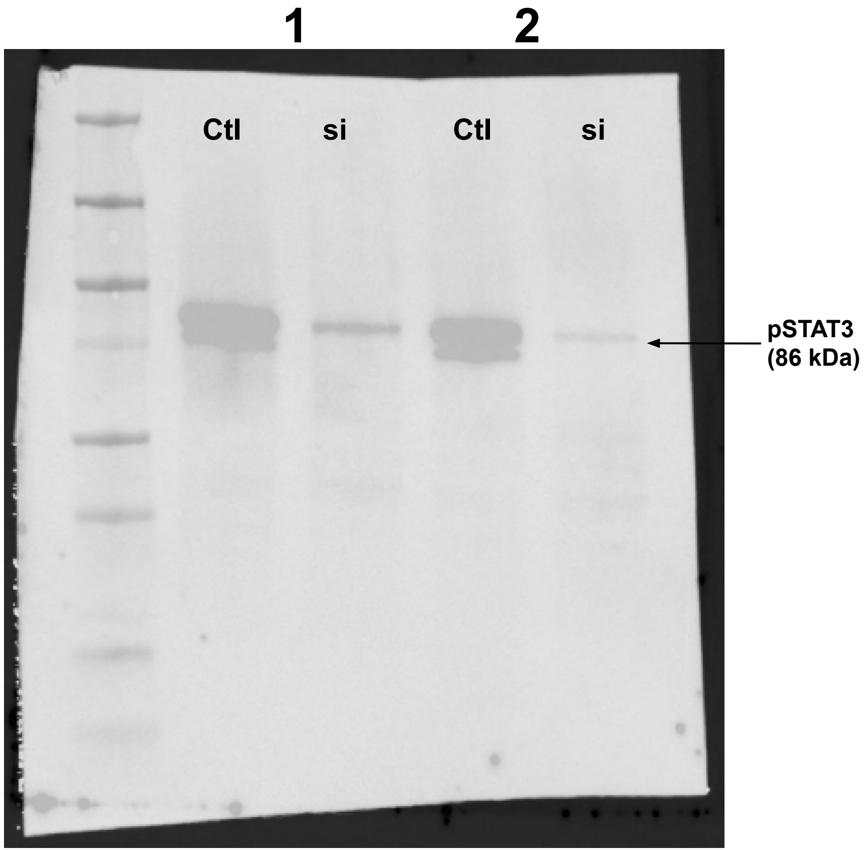

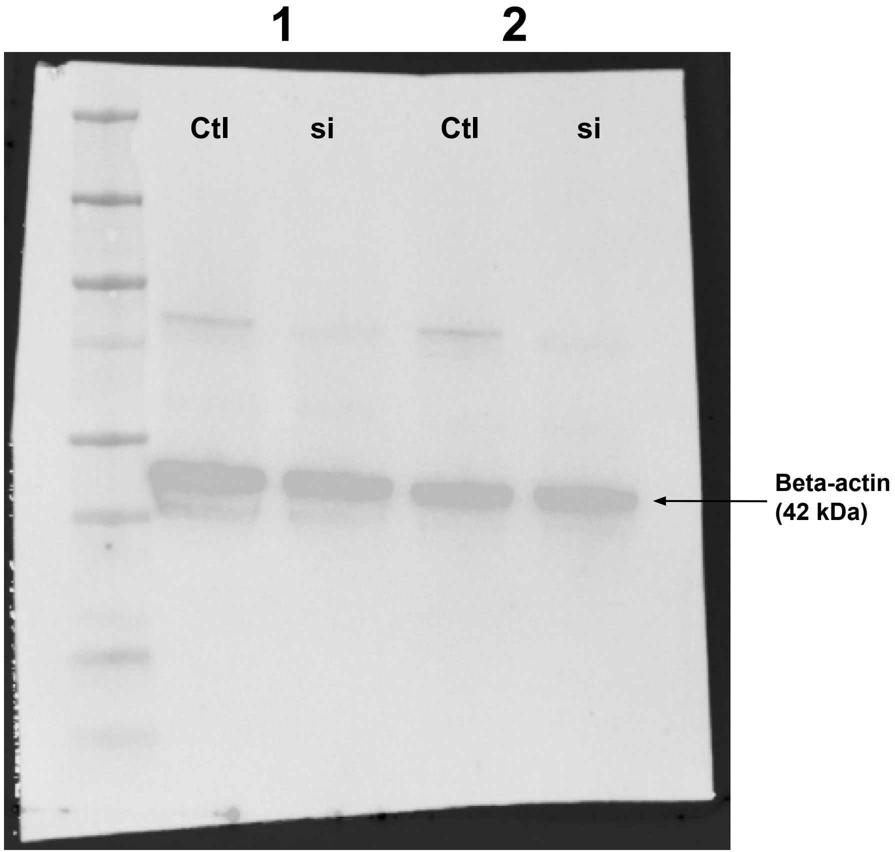

MDA-MB-231 cells_pSTAT3_replicate3

Figure 6

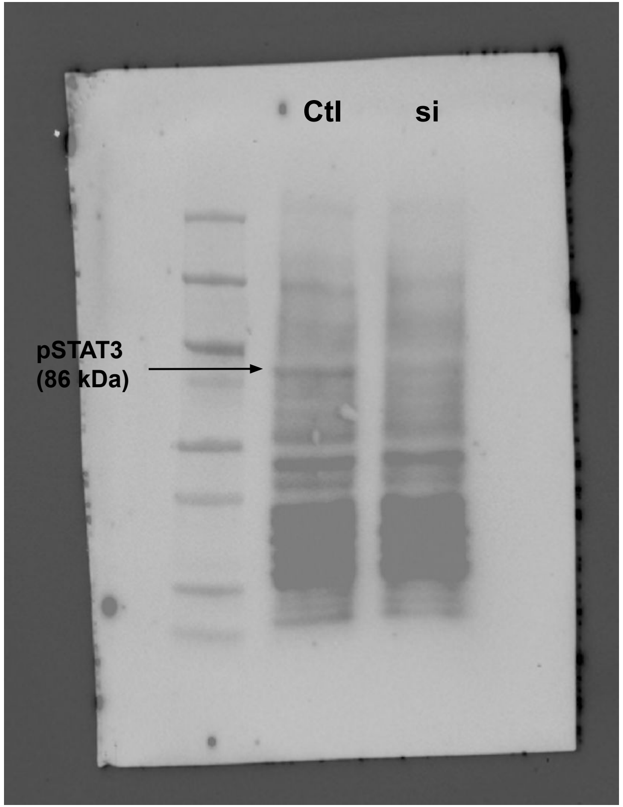

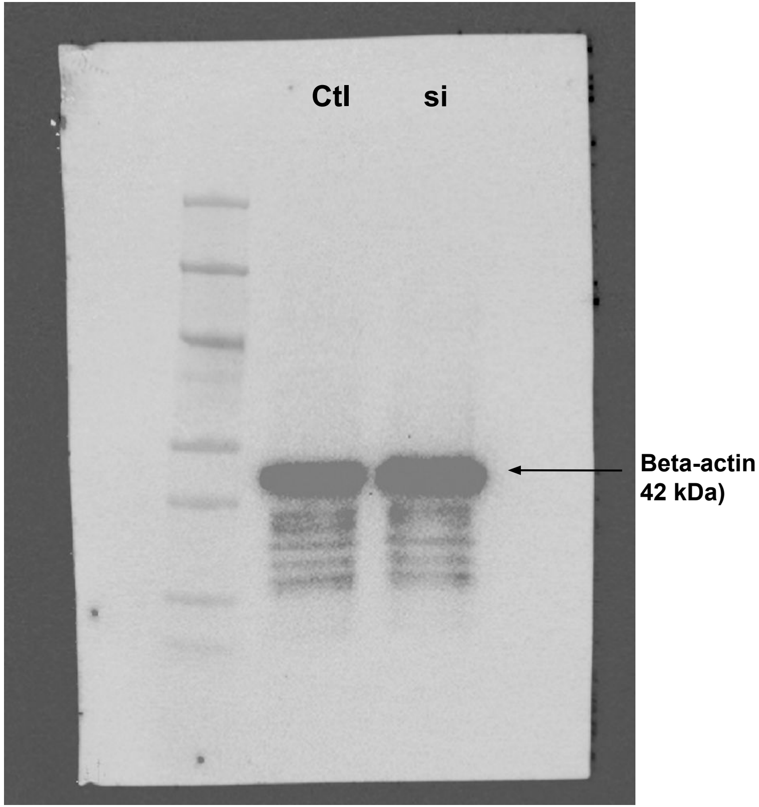

MDA-MB-231 cells_CREB3L1_replicate1

Figure 6

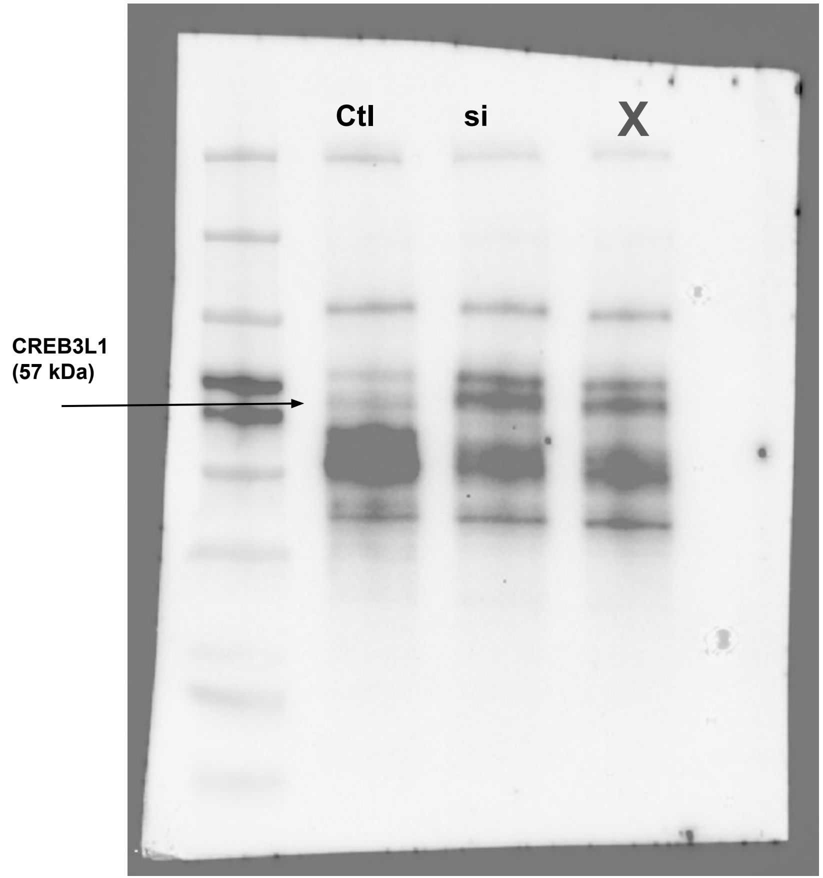

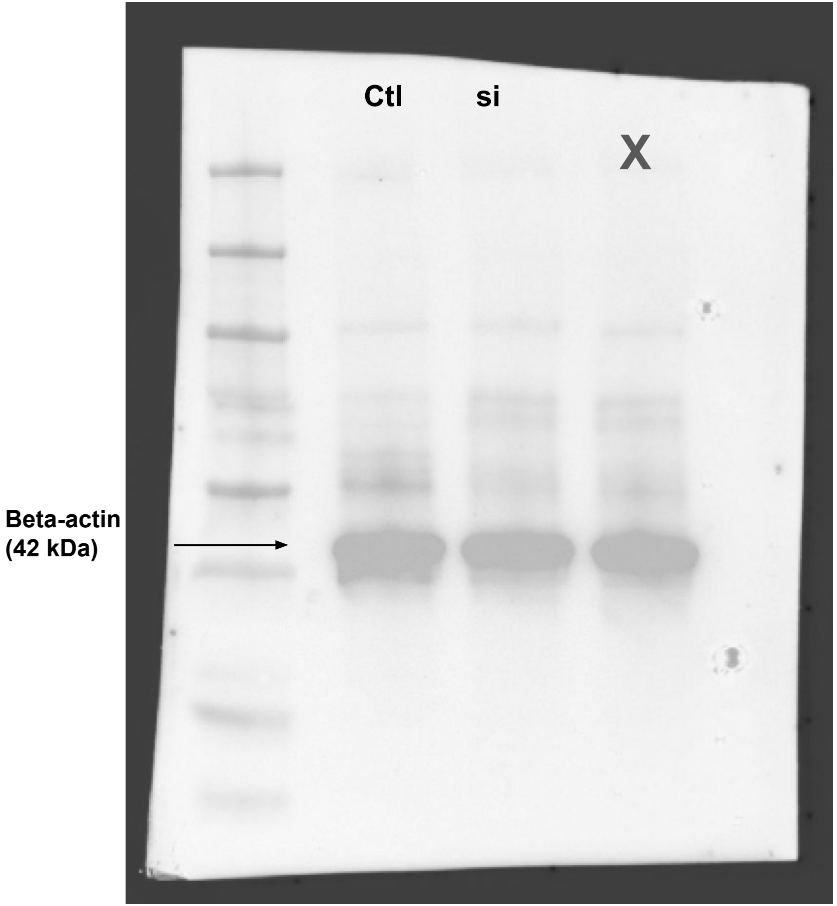

MDA-MB-231 cells_CREB3L1_replicates2+3 Figure 6

- Replicate 3 was used in figure 6a

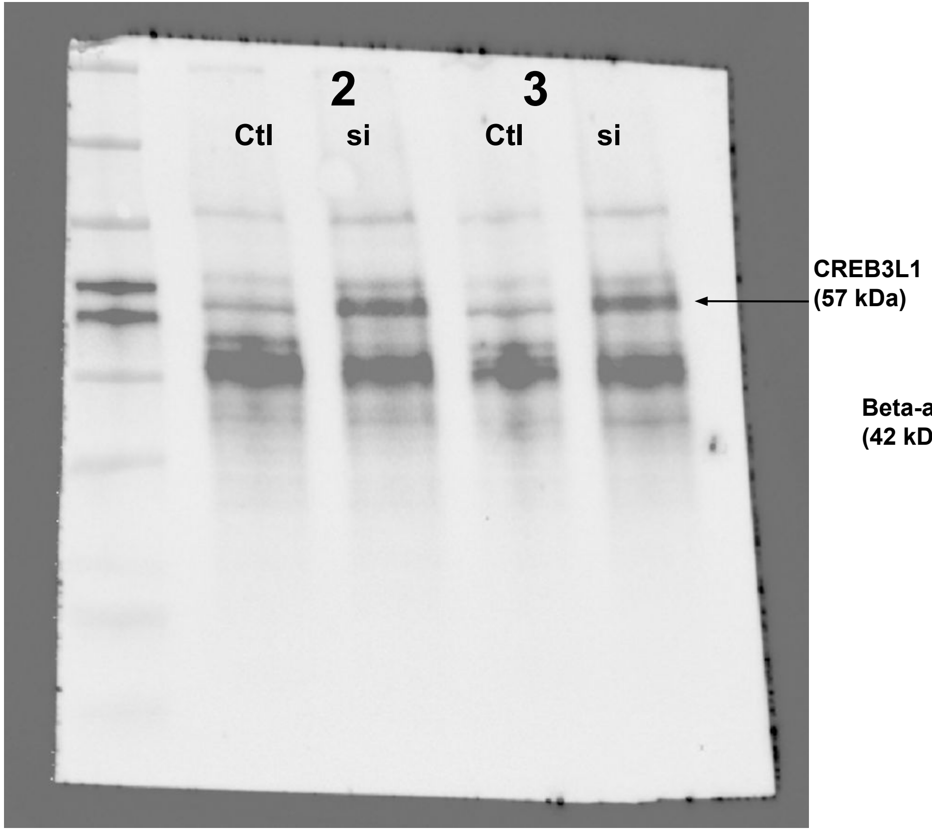

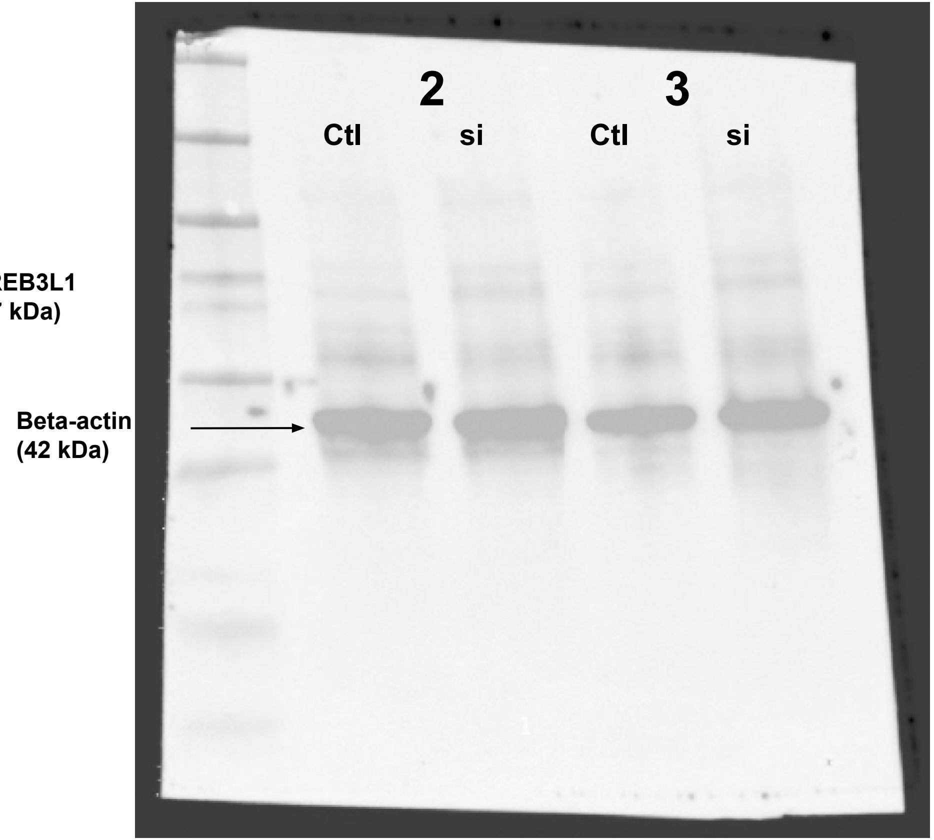

MDA-MB-231 cells_ROR1_replicate1 Figure 6

- Replicate 1 was used in figure 6a

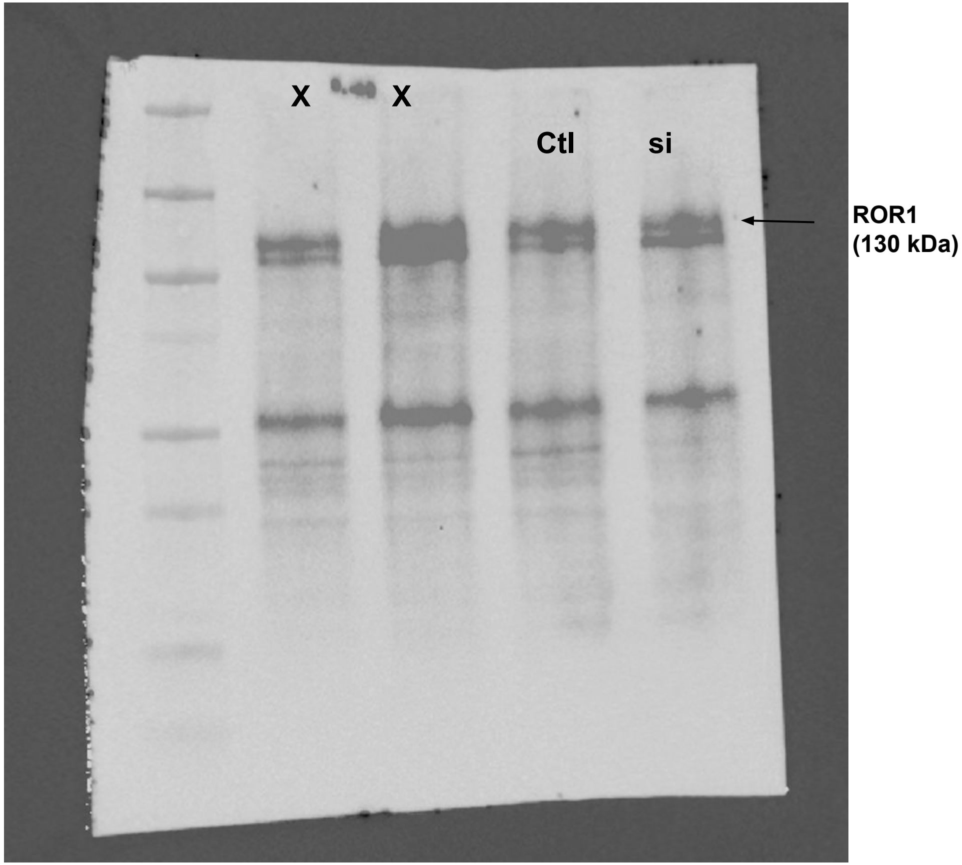

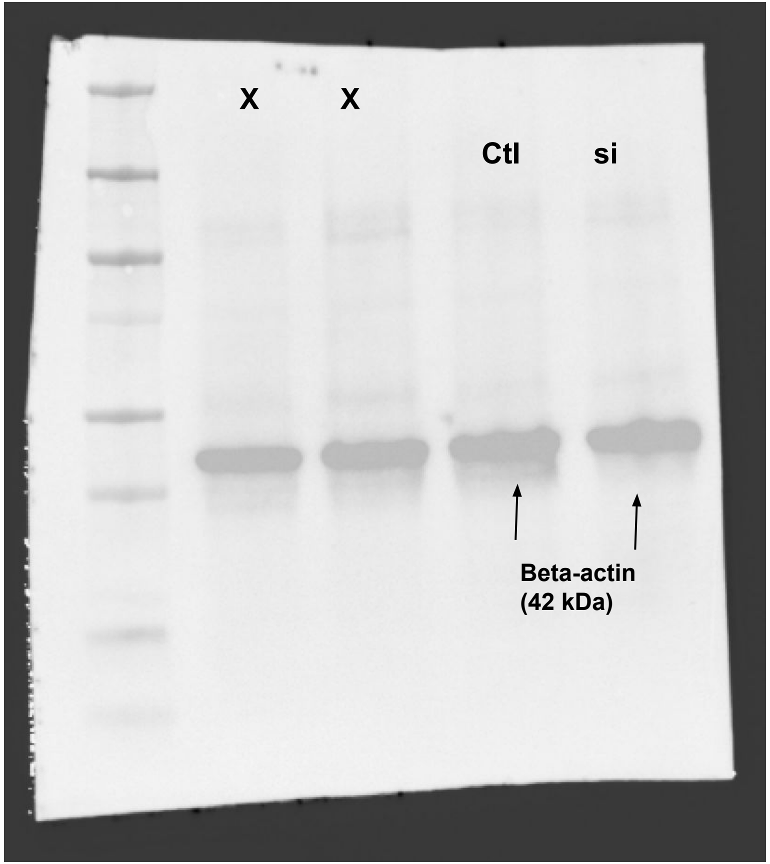

MDA-MB-231 cells_ROR1_replicates 2&3 Figure 6

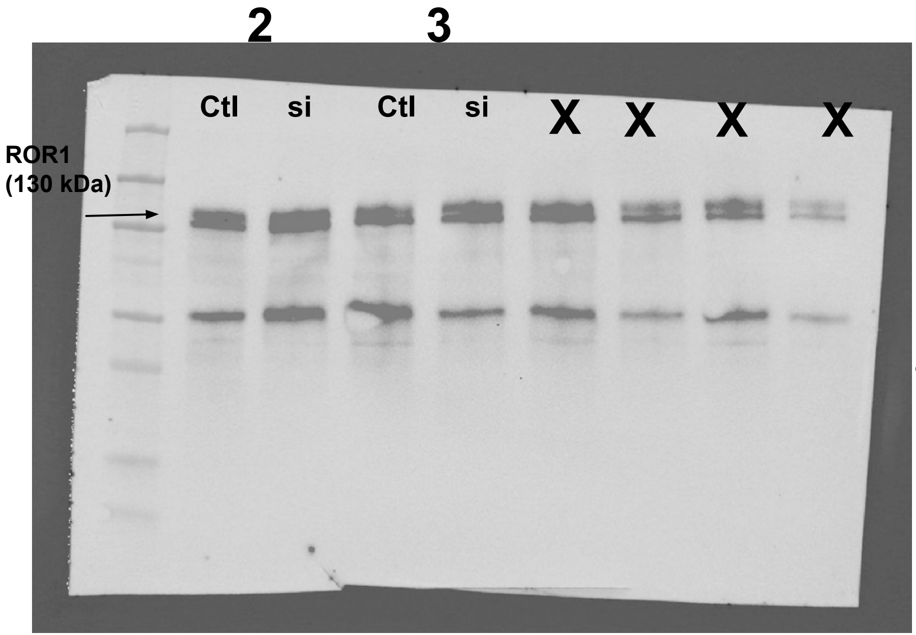

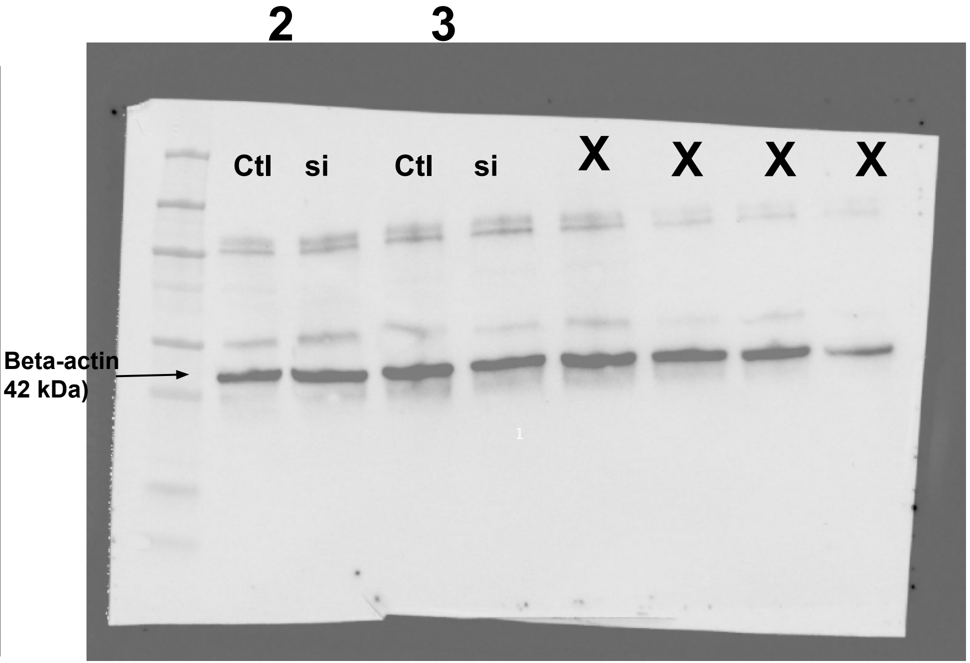

Immunofluorescence images

- HCC1806 control (scRNA) vs siROR1
  - CREB3L1
  - DNMT3B
- MDA-MB-231 control (scRNA) vs siROR1
  - CREB3L1
  - DNMT3A

HCC1806 - CREB3L1 - control - 1
Representative image Figure 4e

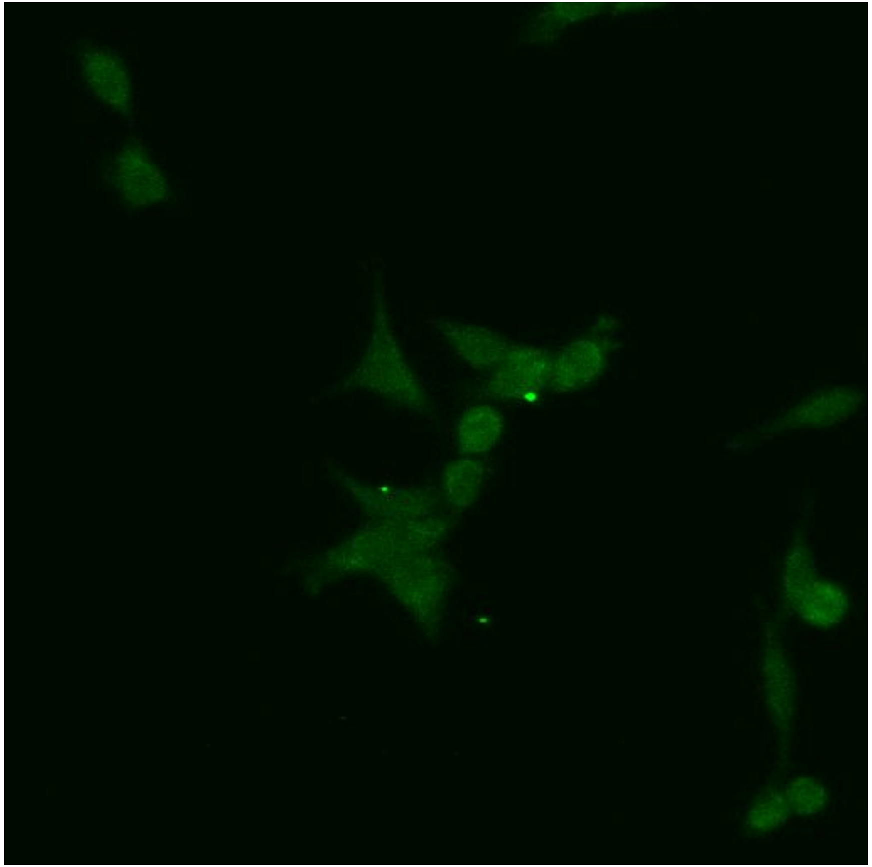
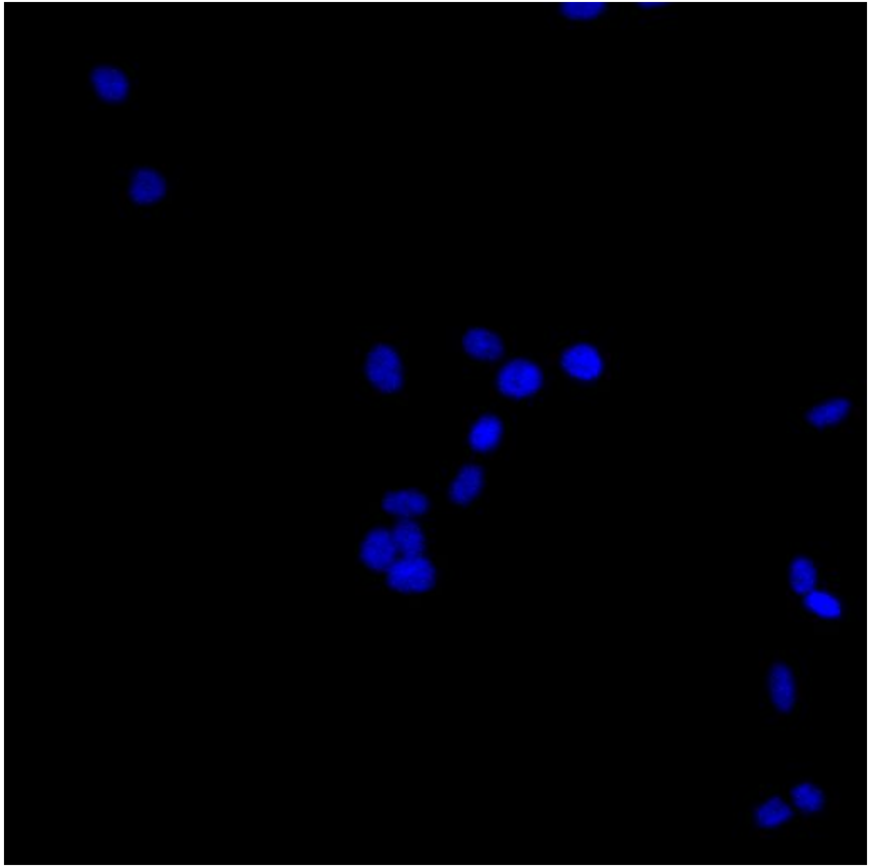

HCC1806 - CREB3L1 - control - 2

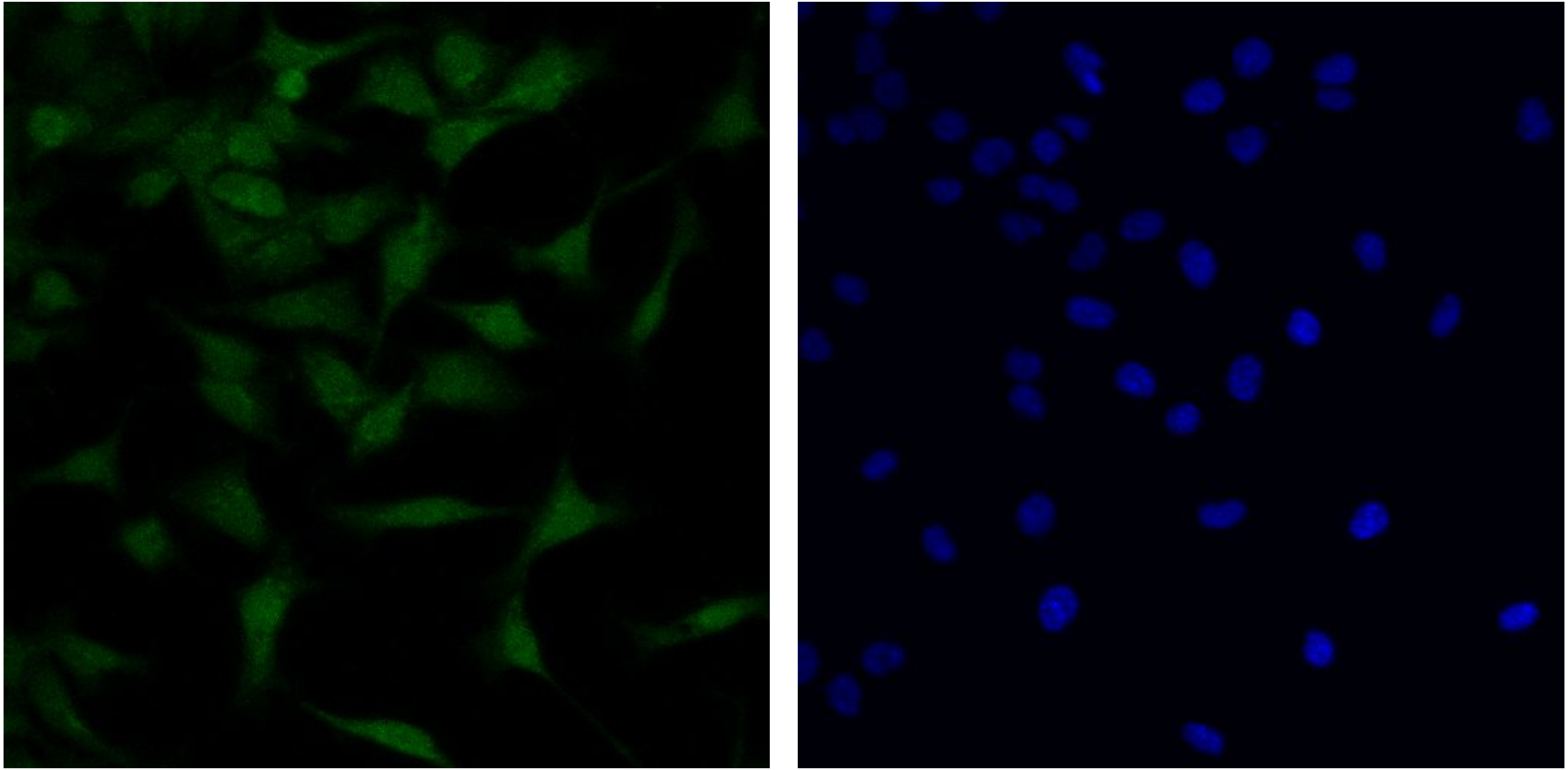

HCC1806 - CREB3L1 - control - 3

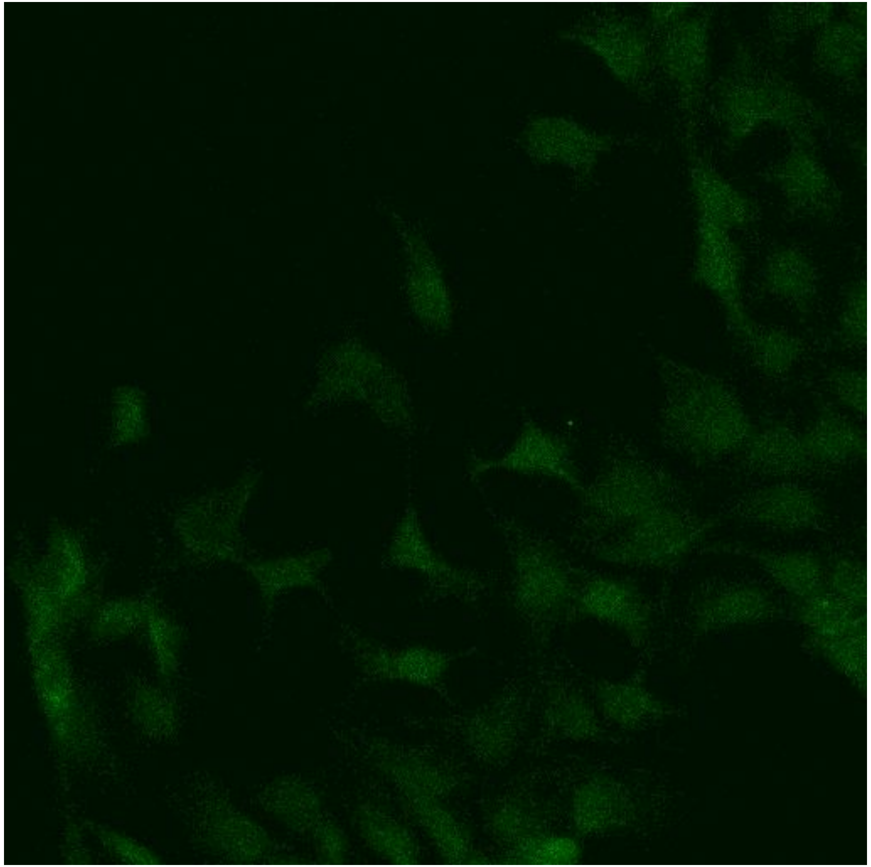

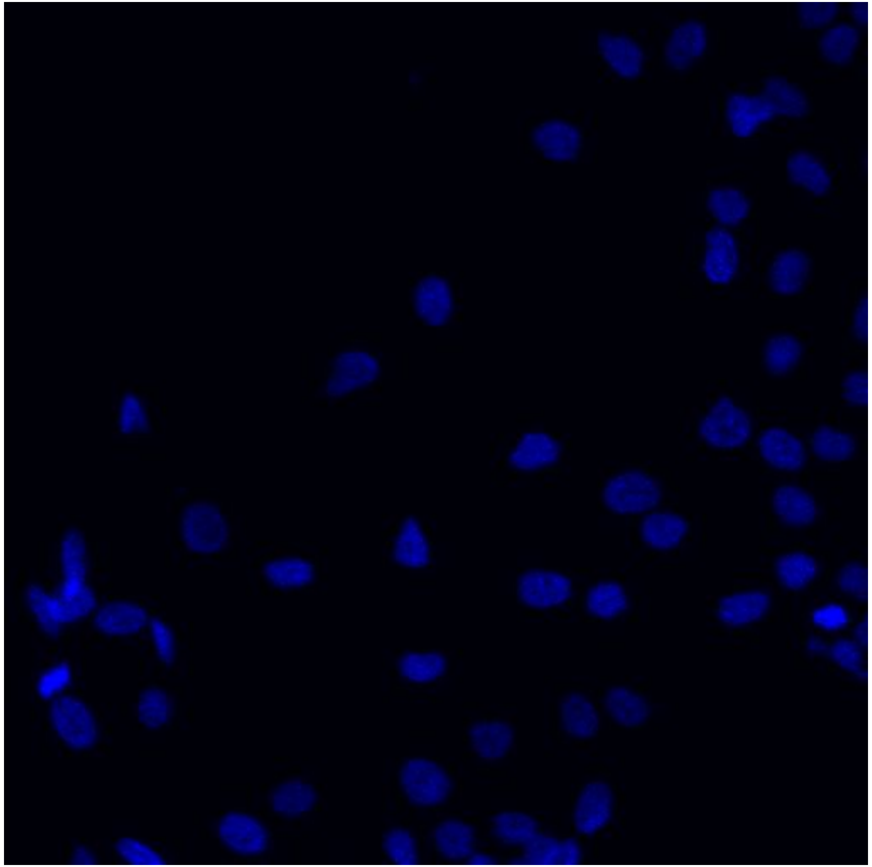

HCC1806 - CREB3L1 - siROR1 - 1
Representative image Figure 4e

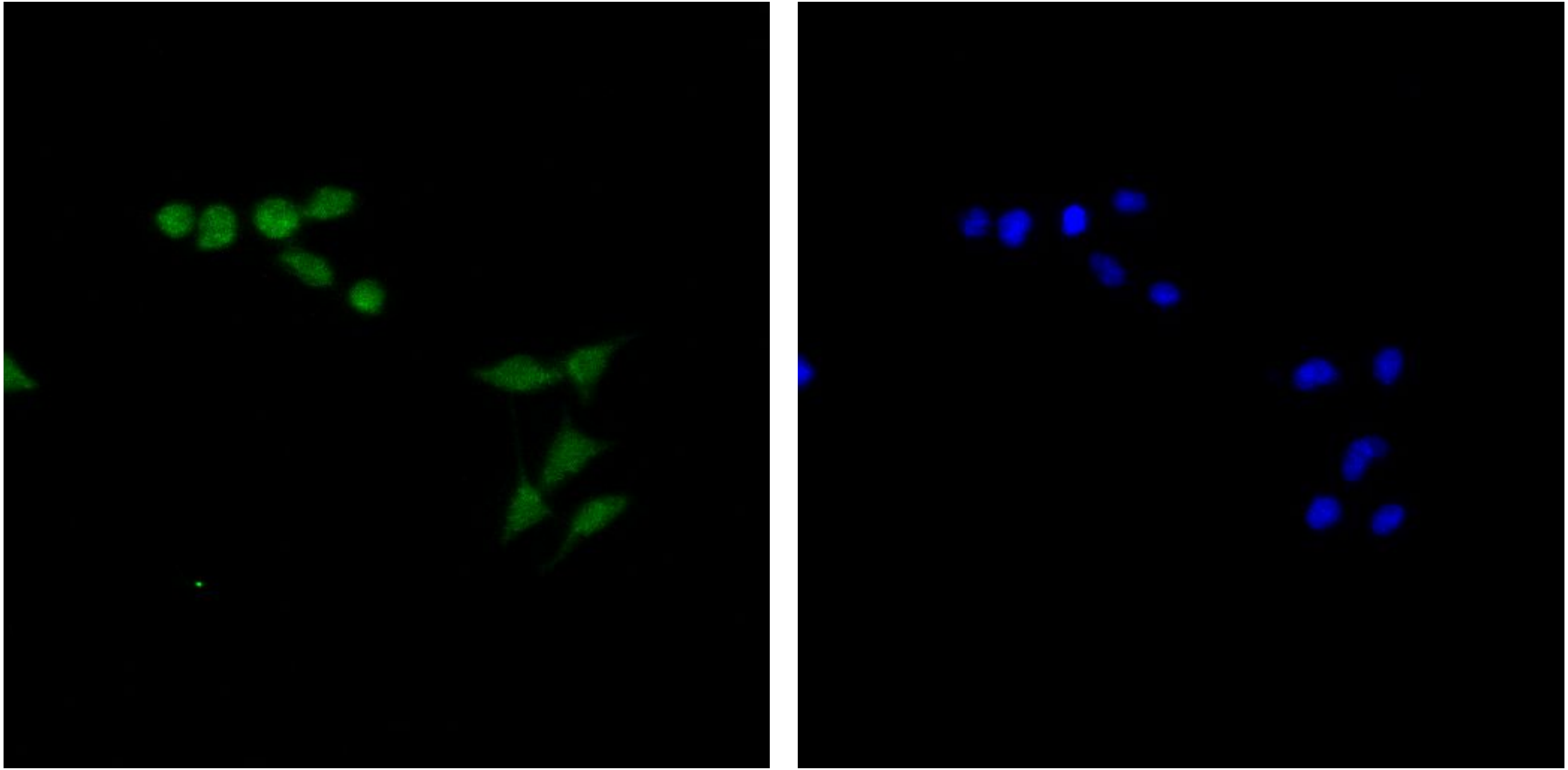

HCC1806 - CREB3L1 - siROR1 - 2

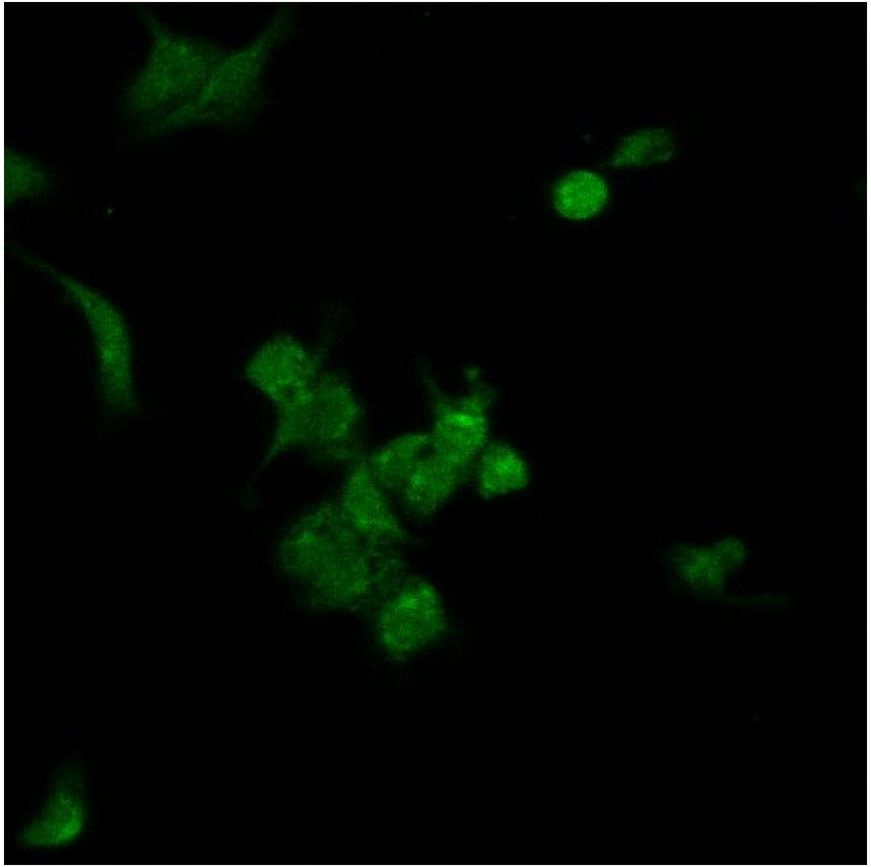

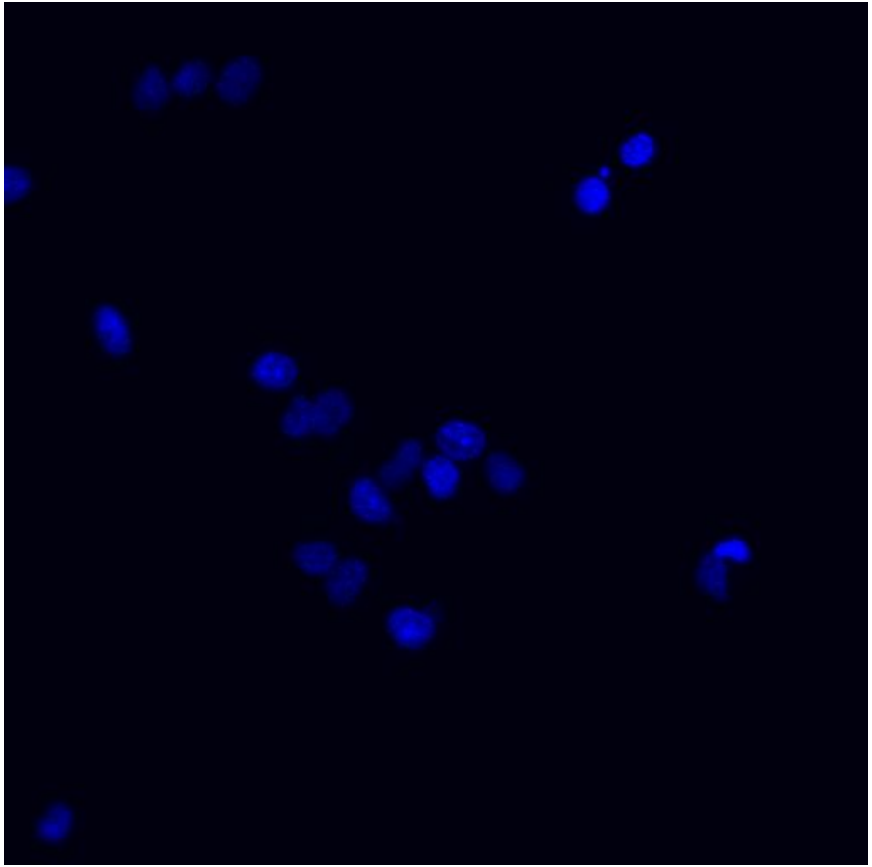

HCC1806 - CREB3L1 - siROR1 - 3

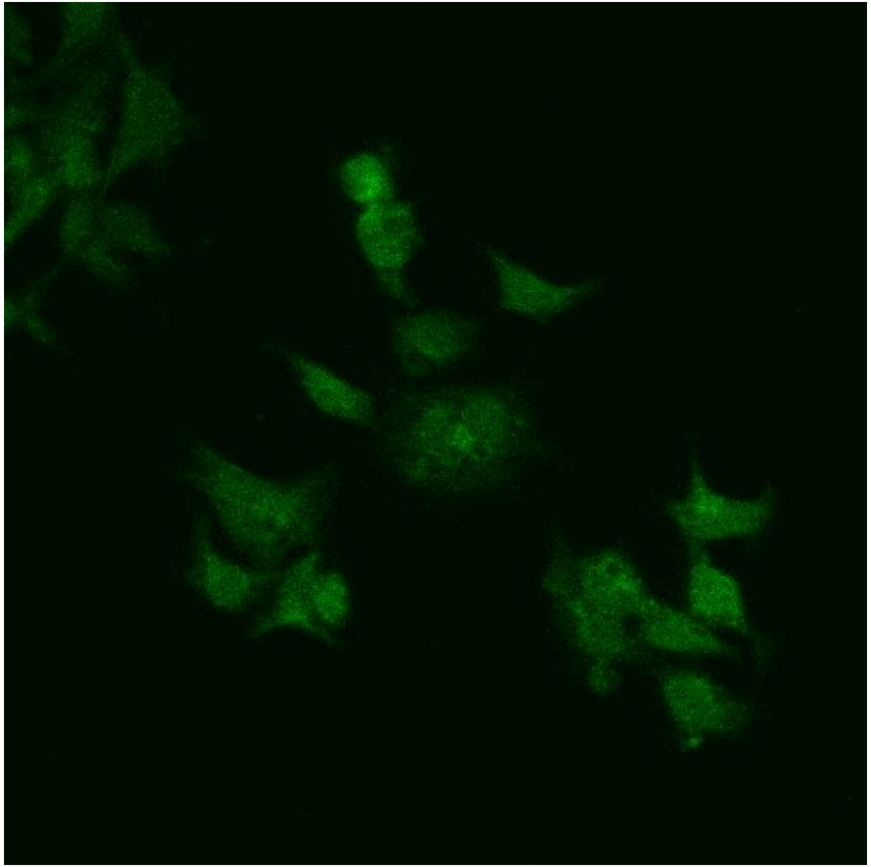

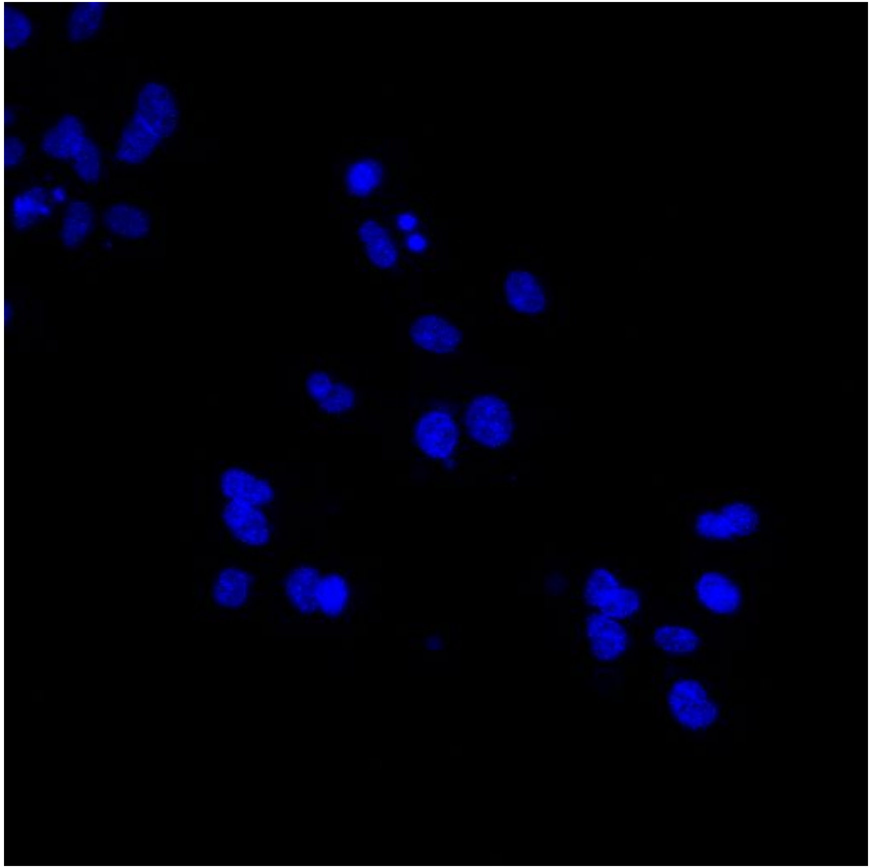

HCC1806 - DNMT3B - control-1

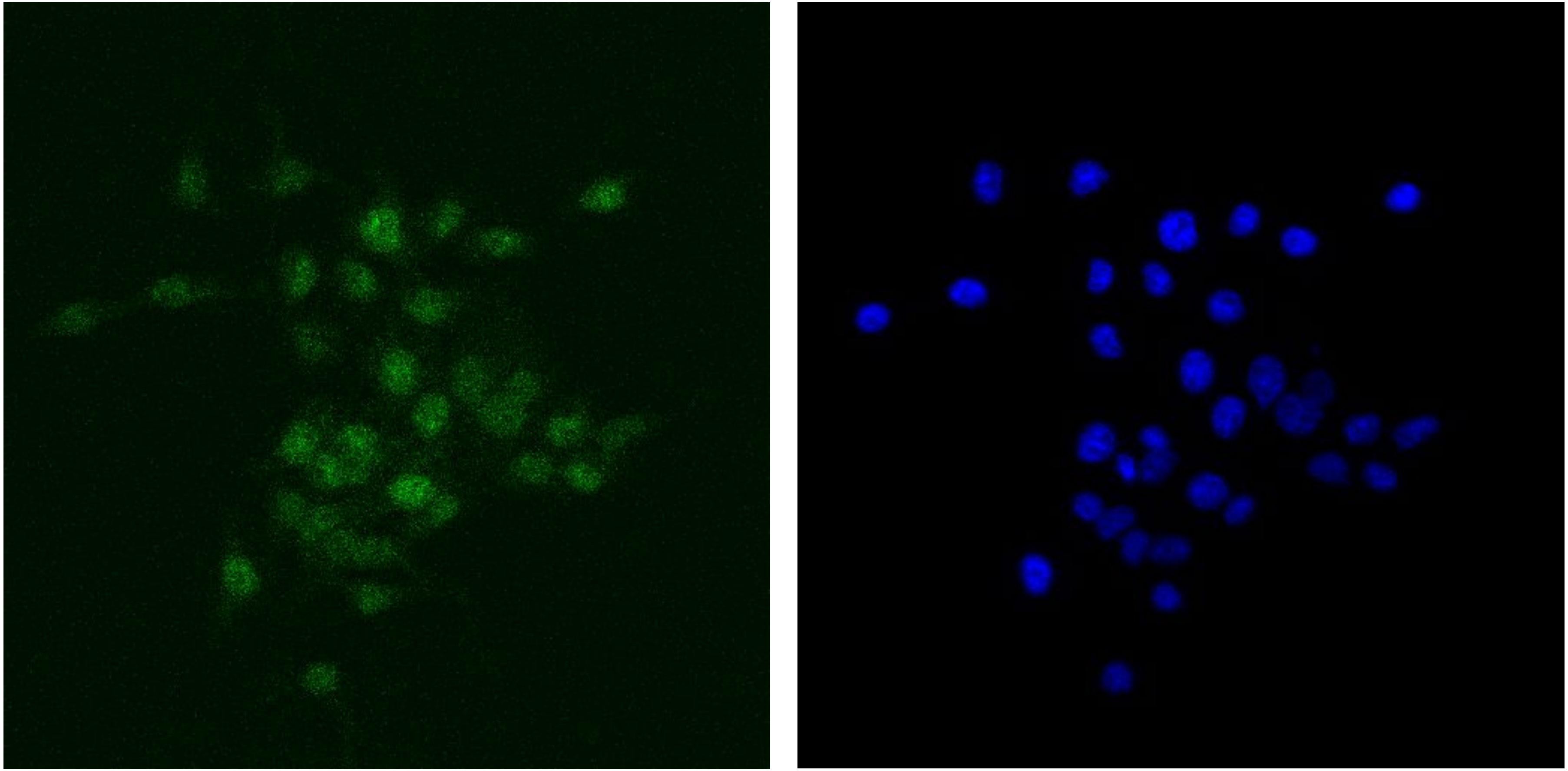

HCC1806 - DNMT3B - control- 2

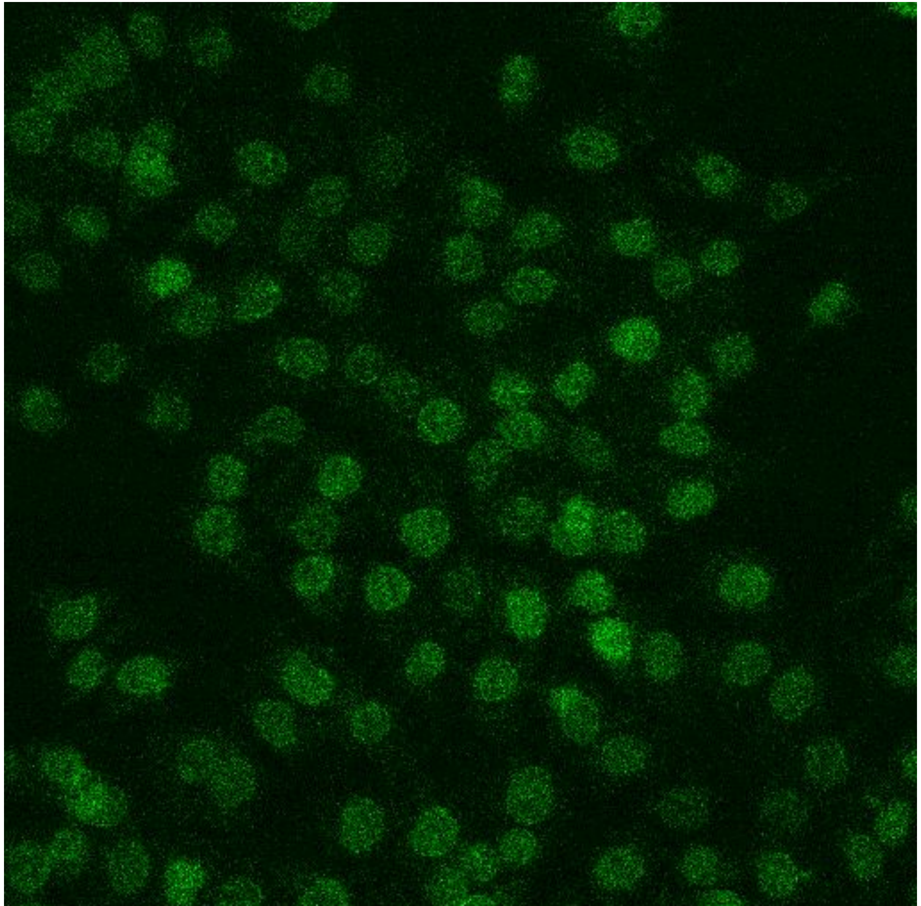

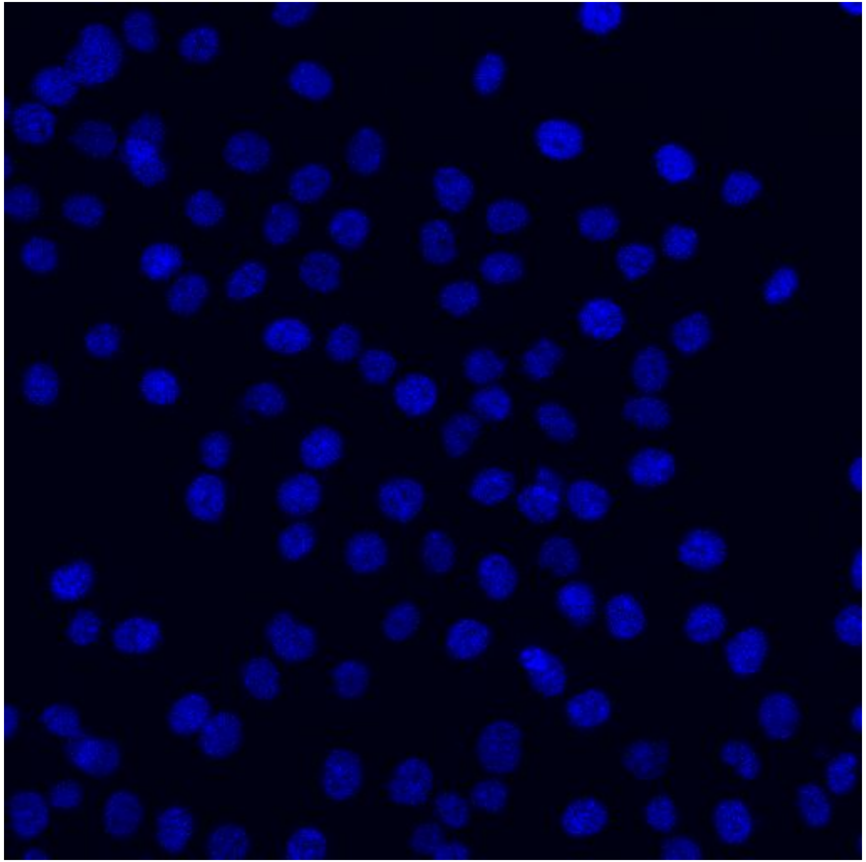

HCC1806 - DNMT3B - control -3

Representative image Figure 4c

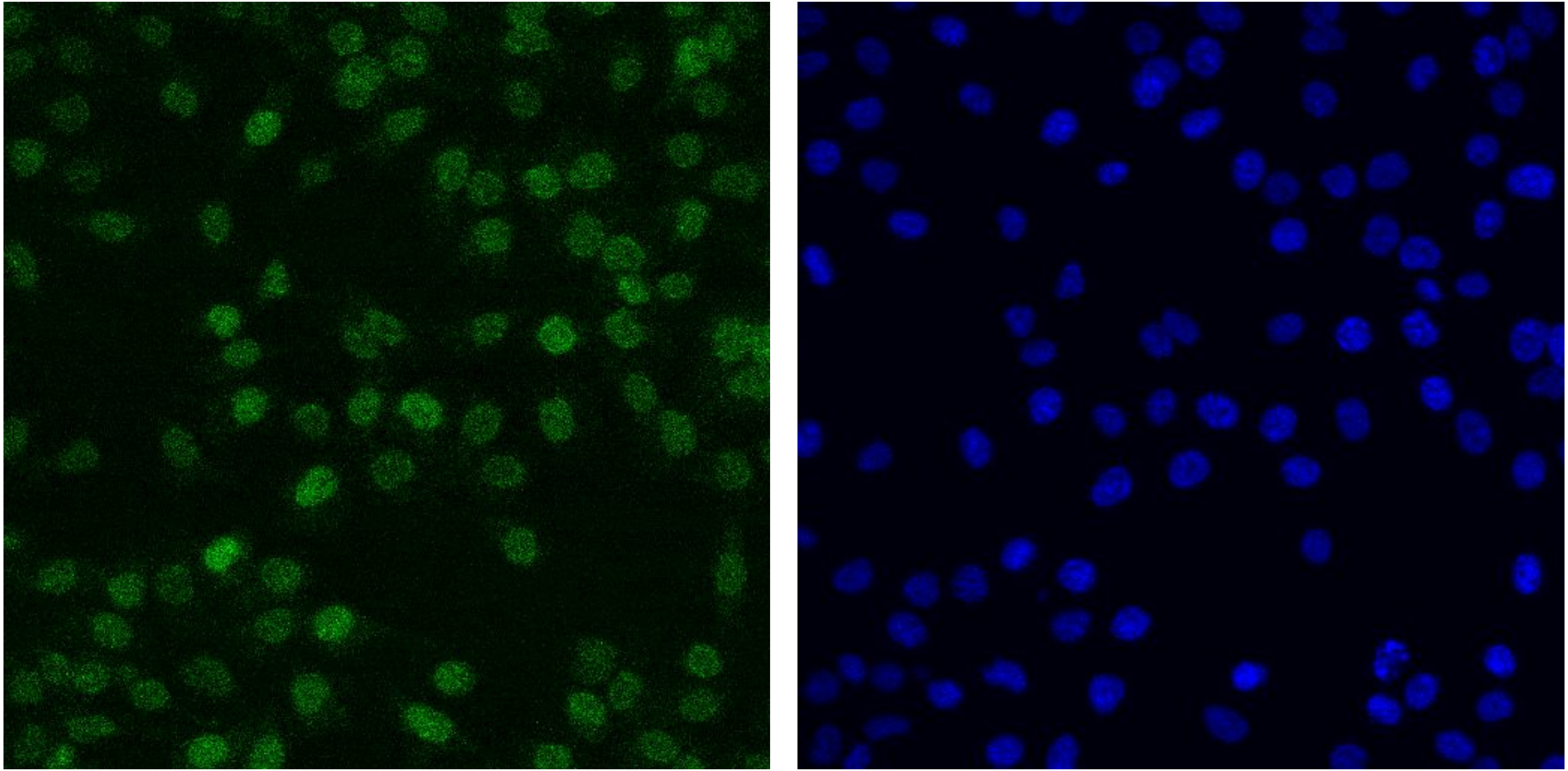

HCC1806 - DNMT3B - siROR1 - 1

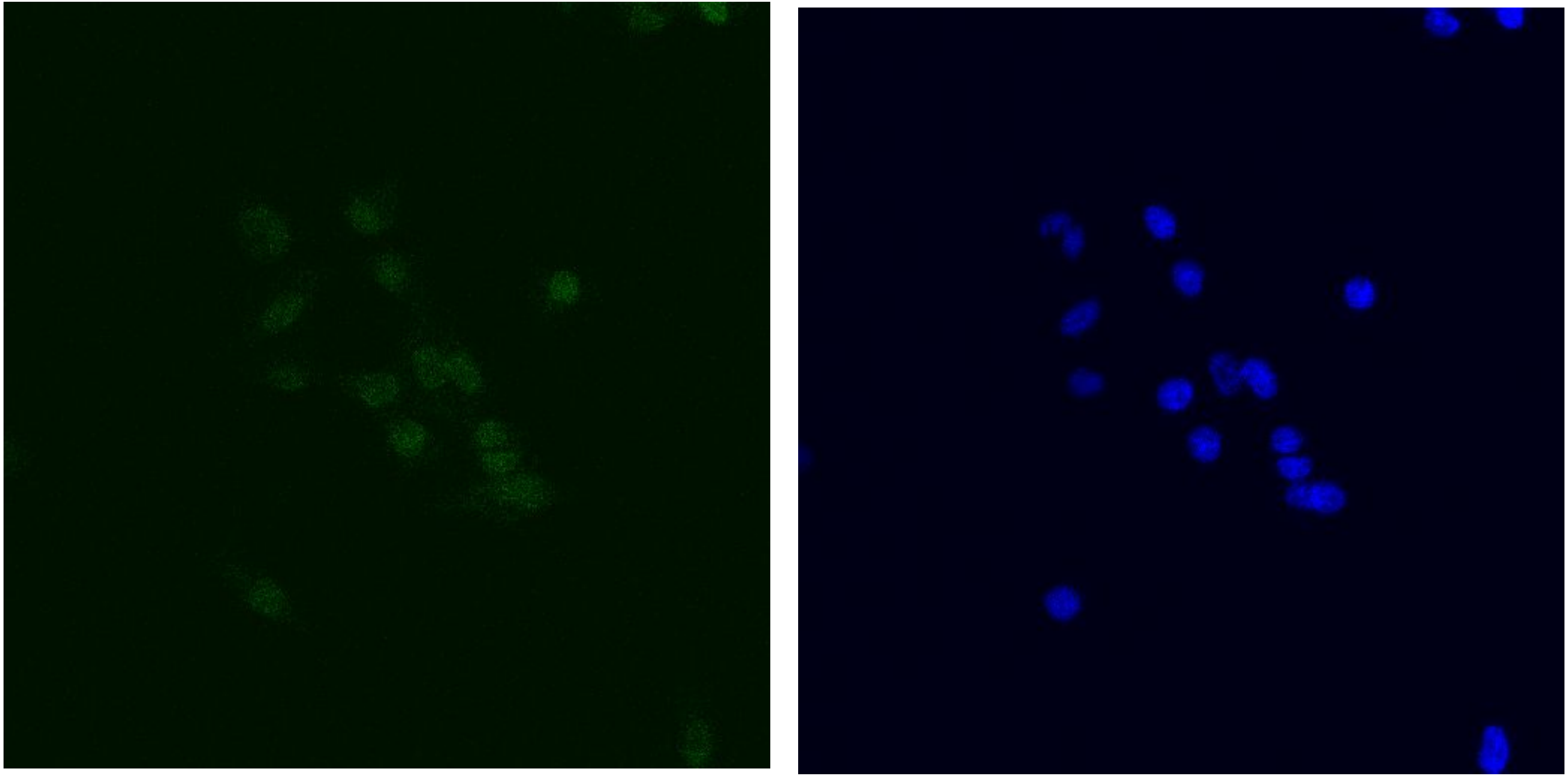

HCC1806 - DNMT3B - siROR1 - 2

- Representative image Figure 4c

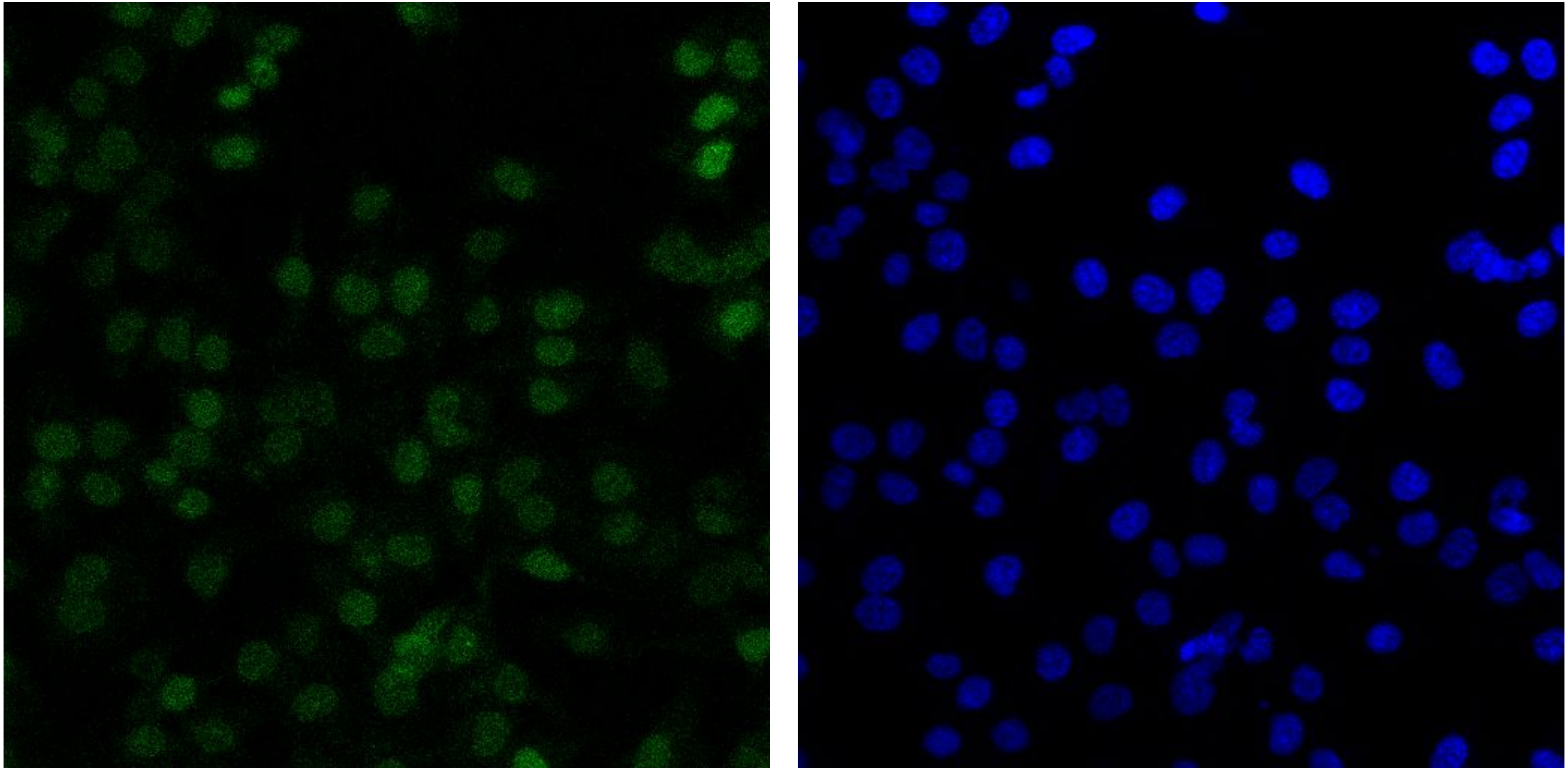

HCC1806 - DNMT3B - siROR1 - 3

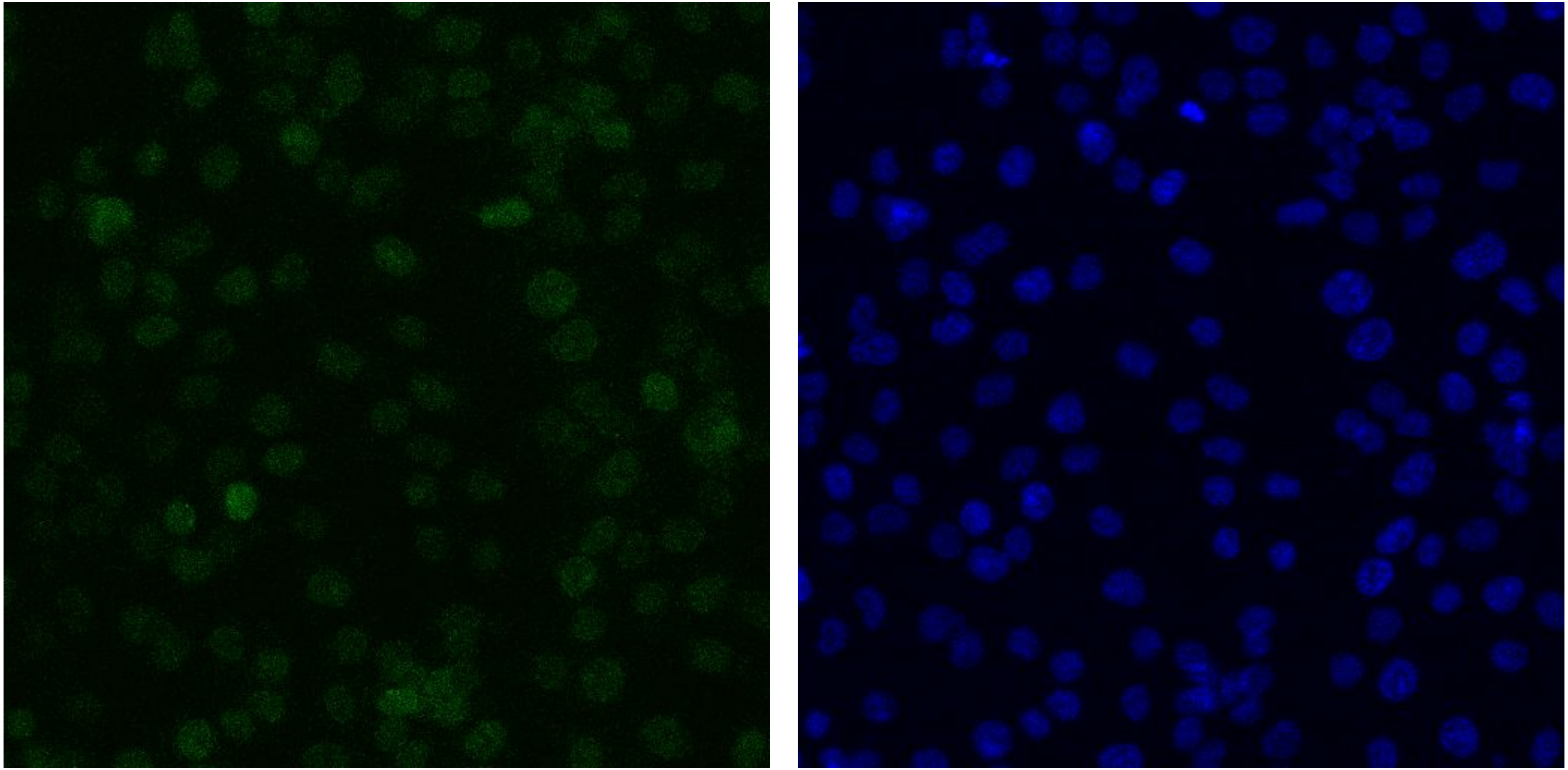

MDA-MB-231 - CREB3L1 - control-1

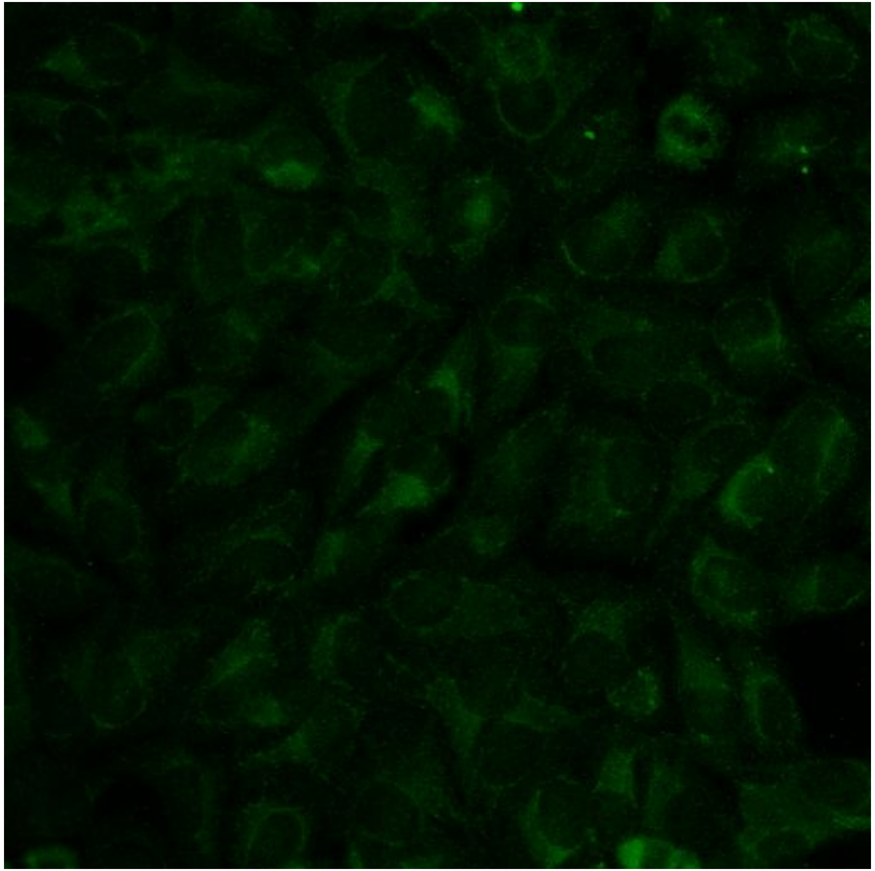

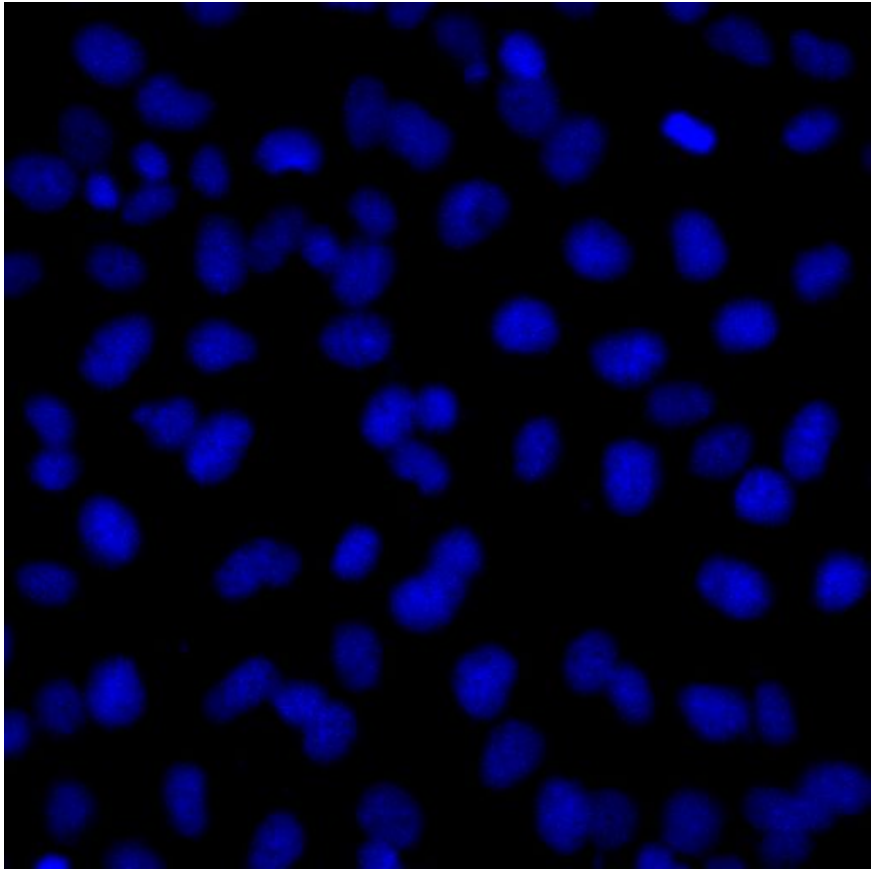

MDA-MB-231 - CREB3L1 - control-2

- Representative image Figure 4e

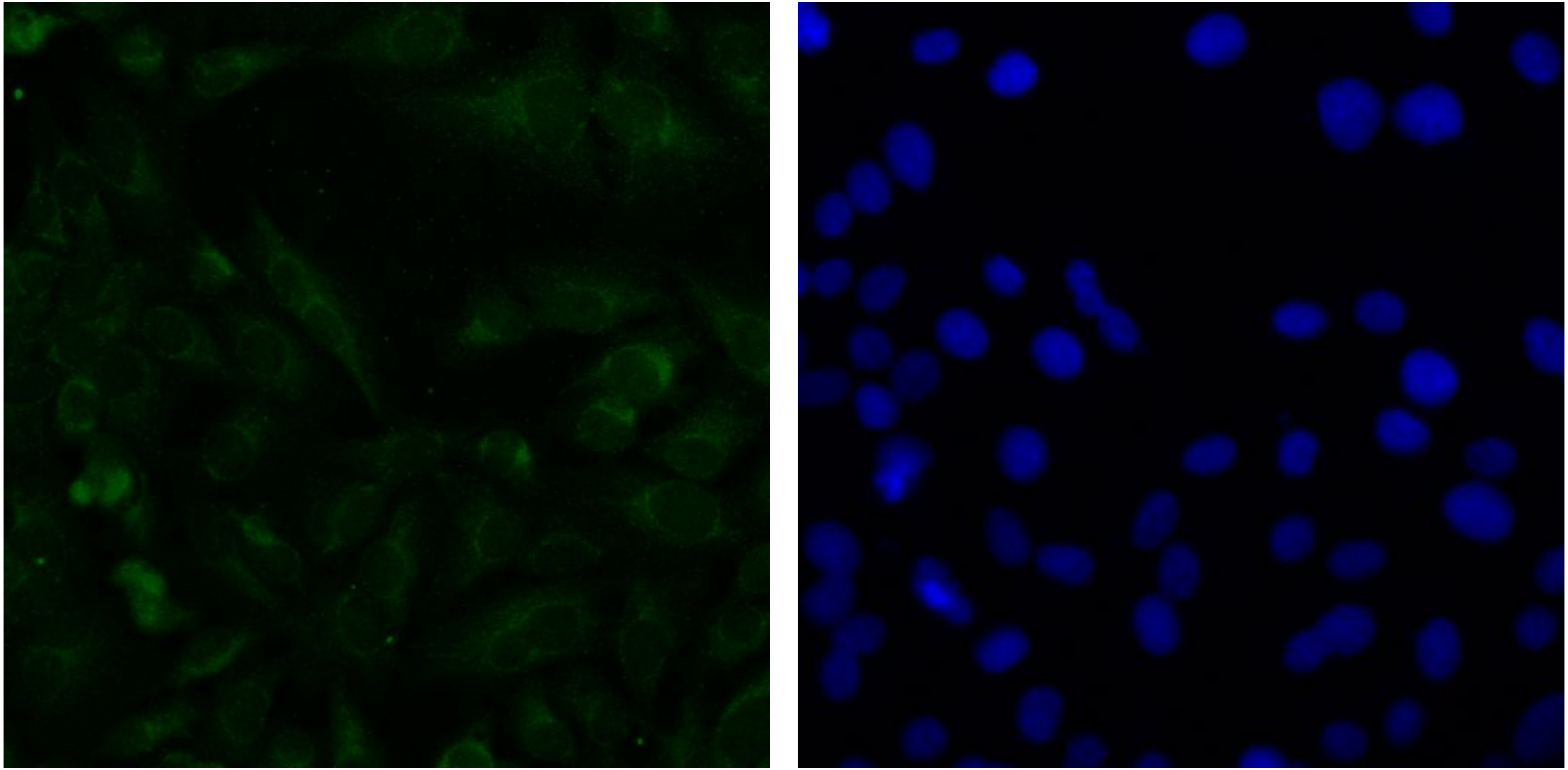

MDA-MB-231 - CREB3L1 - control-3

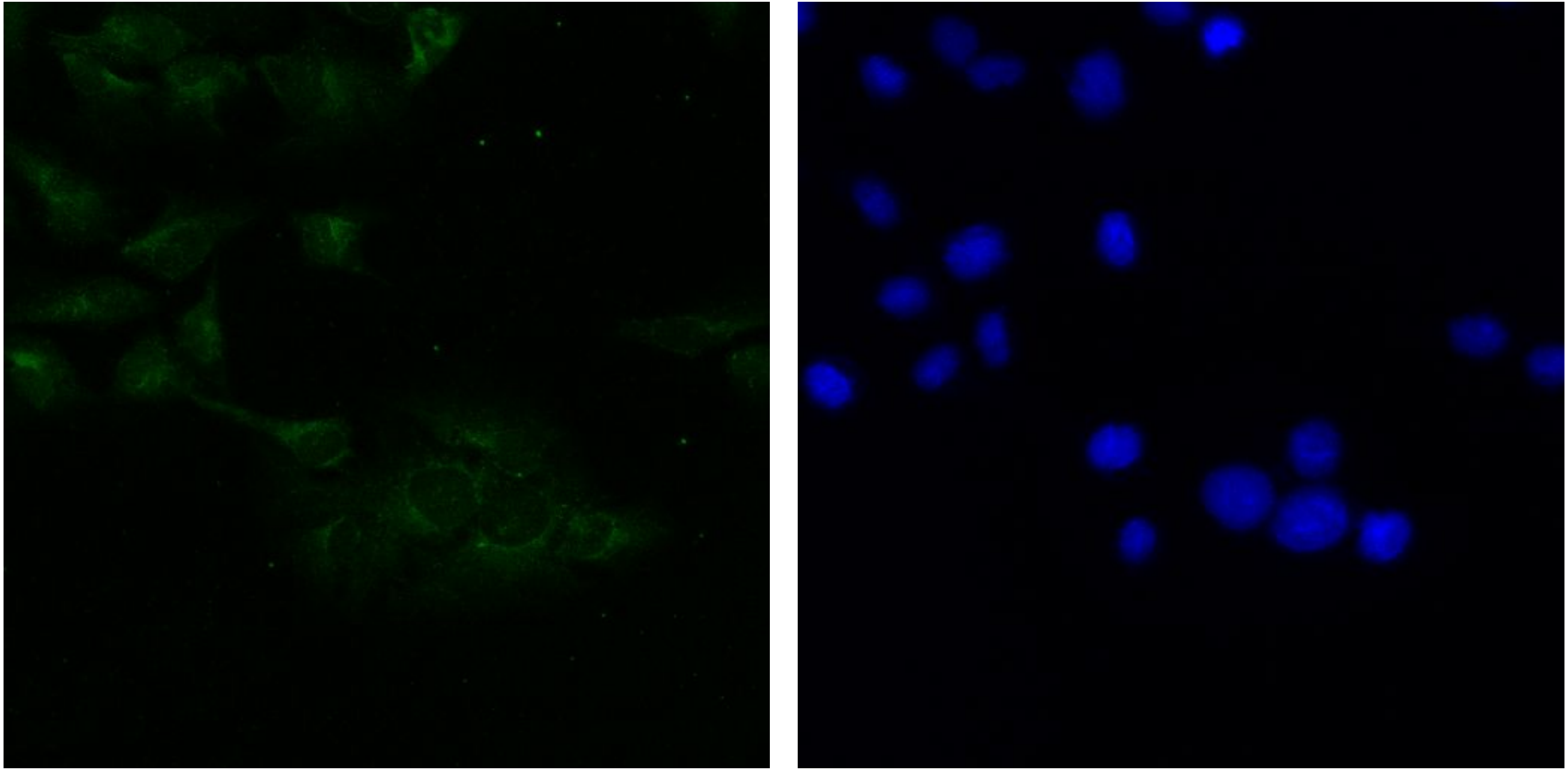

MDA-MB-231 - CREB3L1 - siROR1-1

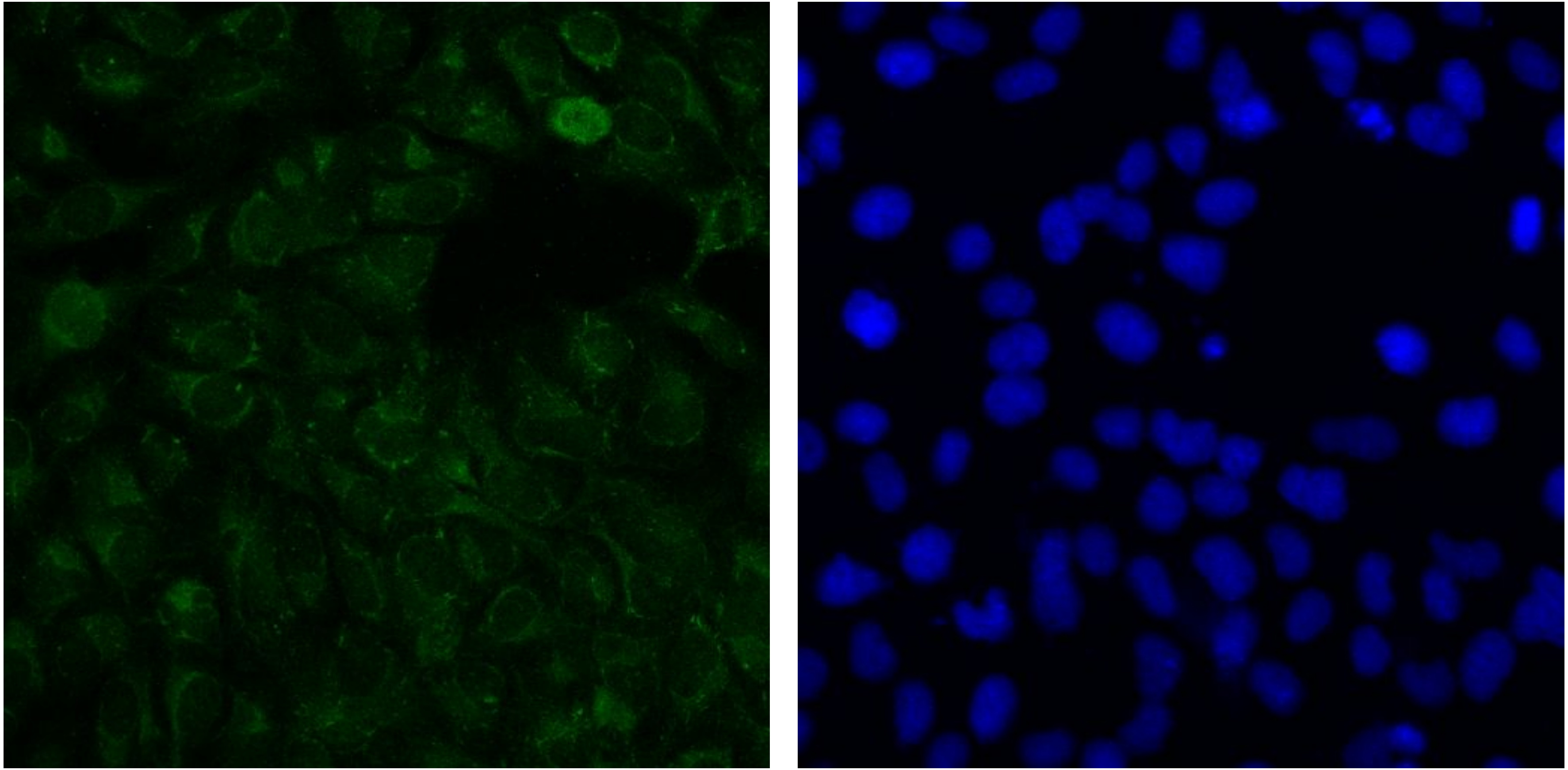

MDA-MB-231 - CREB3L1 - siROR1-2

- Representative image Figure 4e

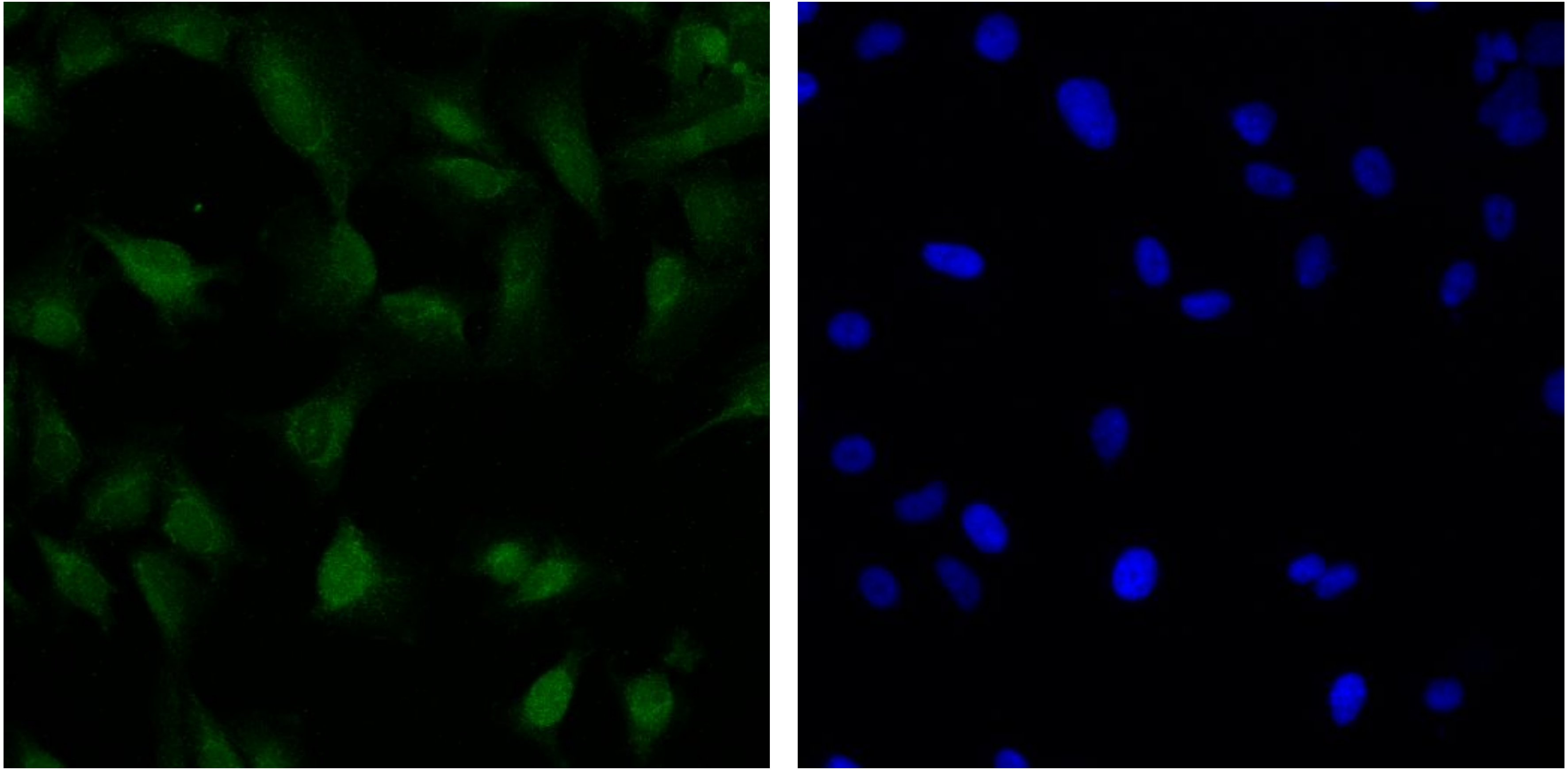

MDA-MB-231 - CREB3L1 - siROR1-3

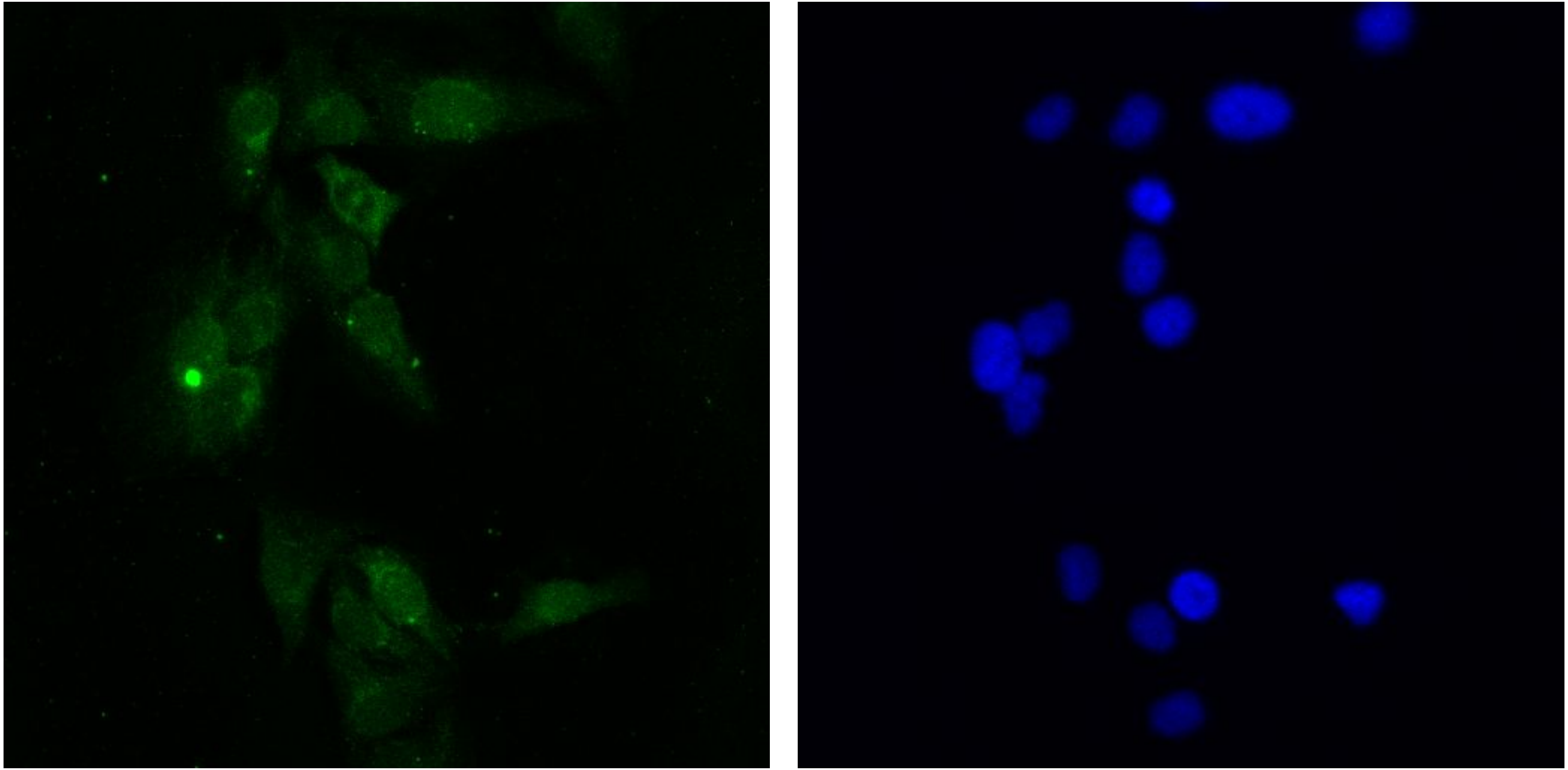

MDA-MB-231 - DNMT3A - control-1

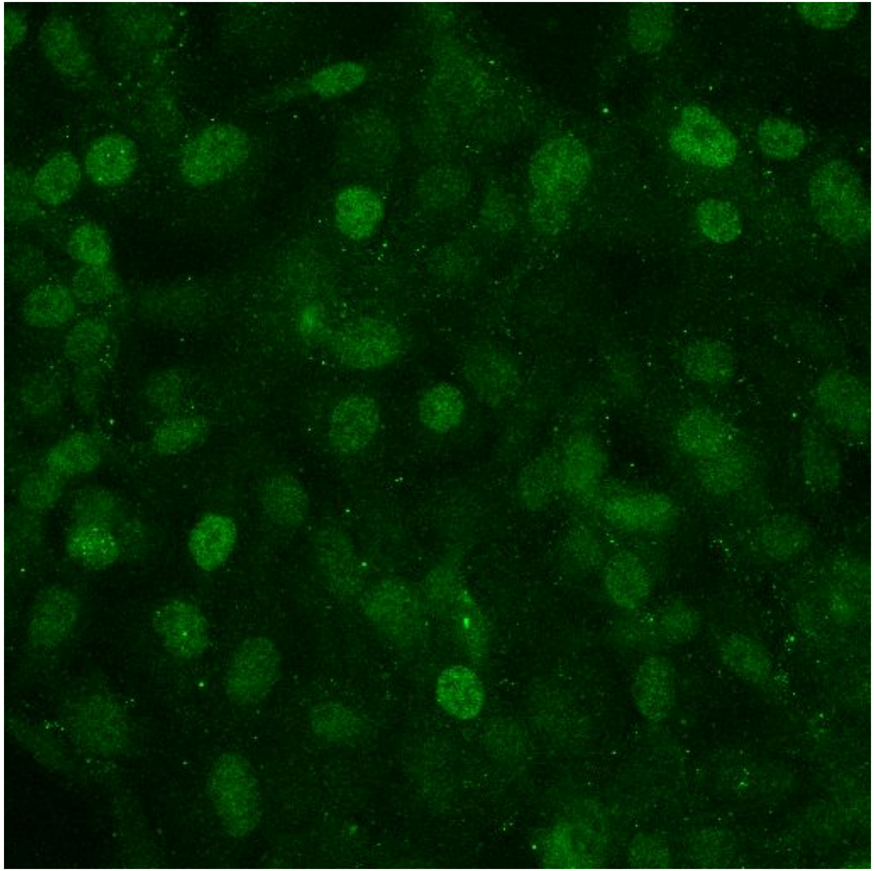

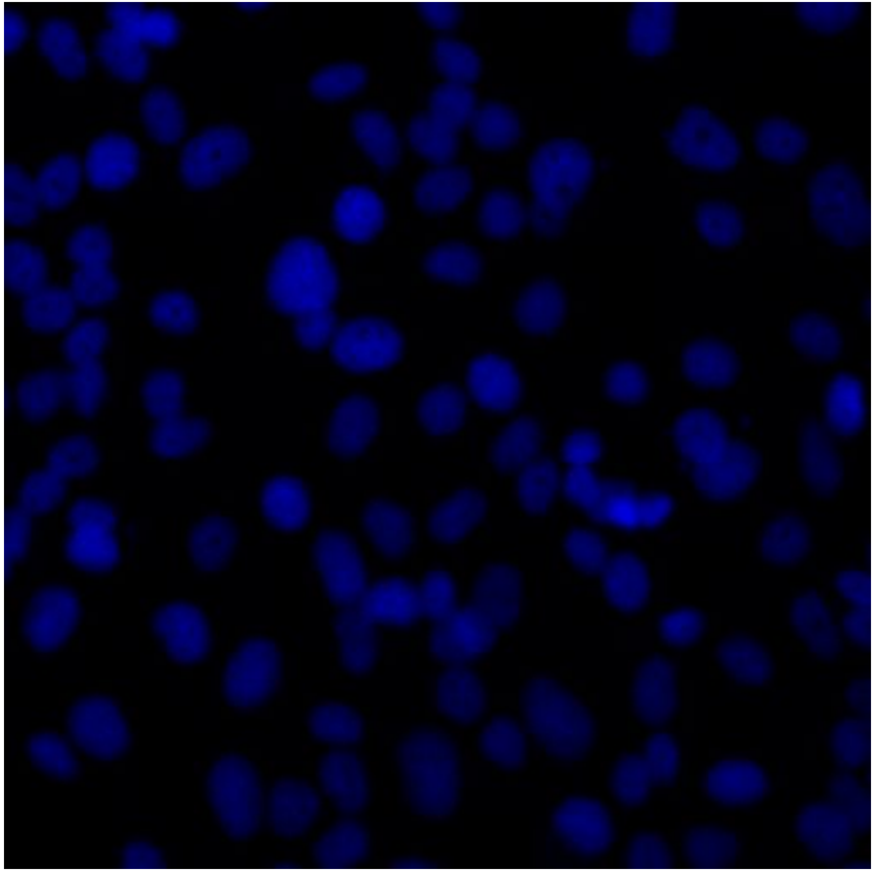

MDA-MB-231 - DNMT3A - control-2

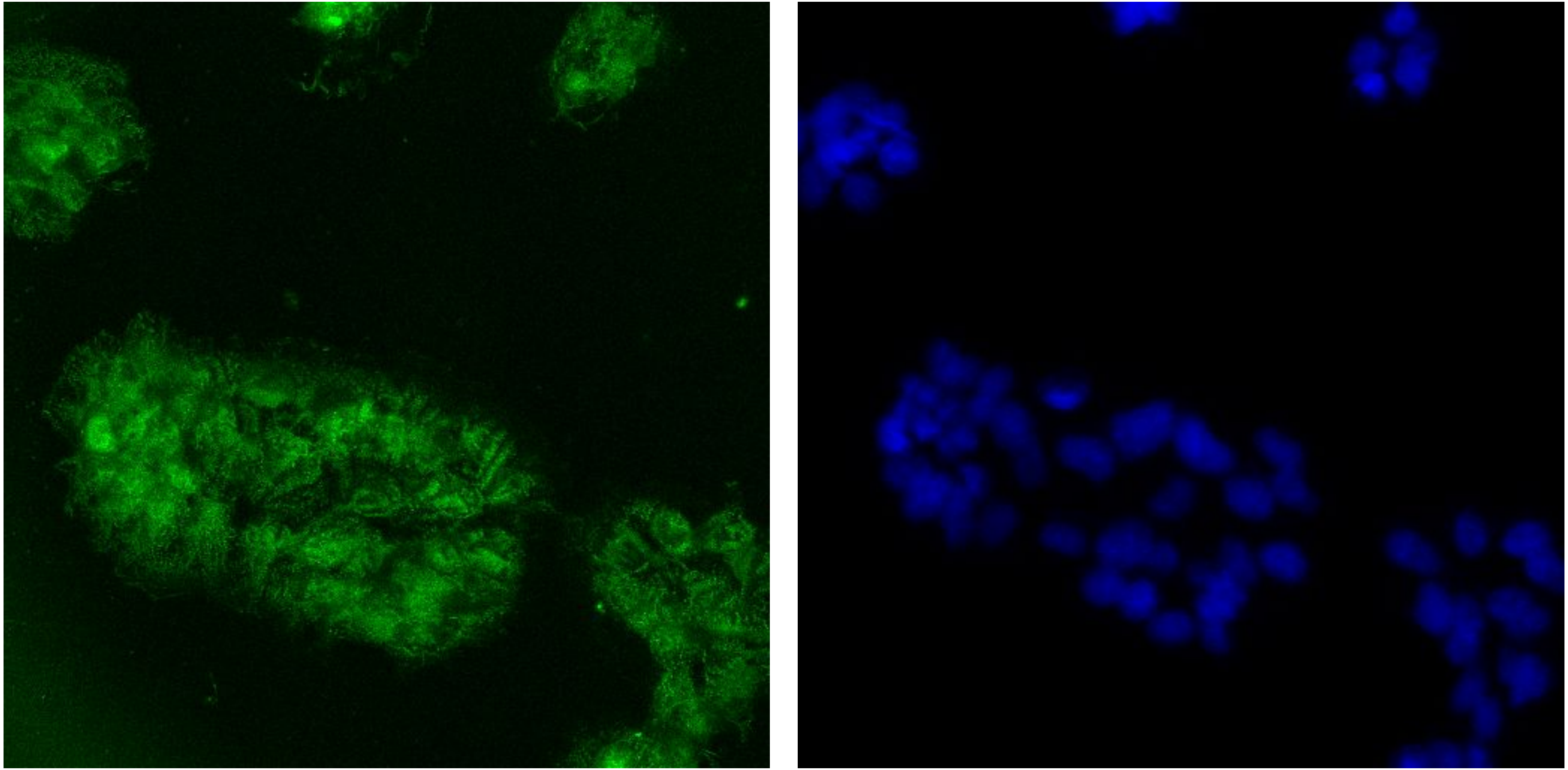

MDA-MB-231 - DNMT3A - control-3

- Representative image figure 4c

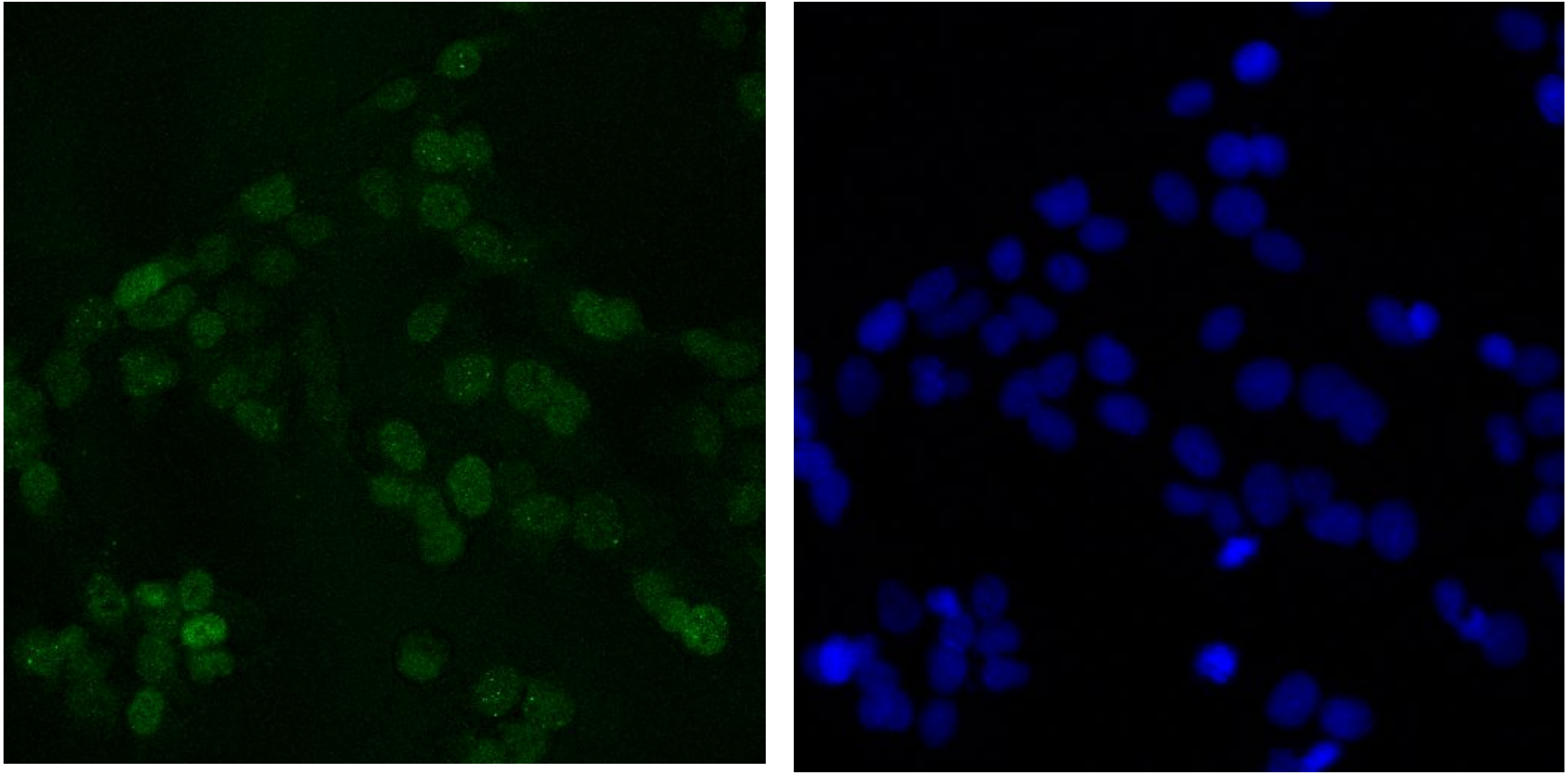

MDA-MB-231 - DNMT3A - siROR1-1

- Representative image Figure 4c

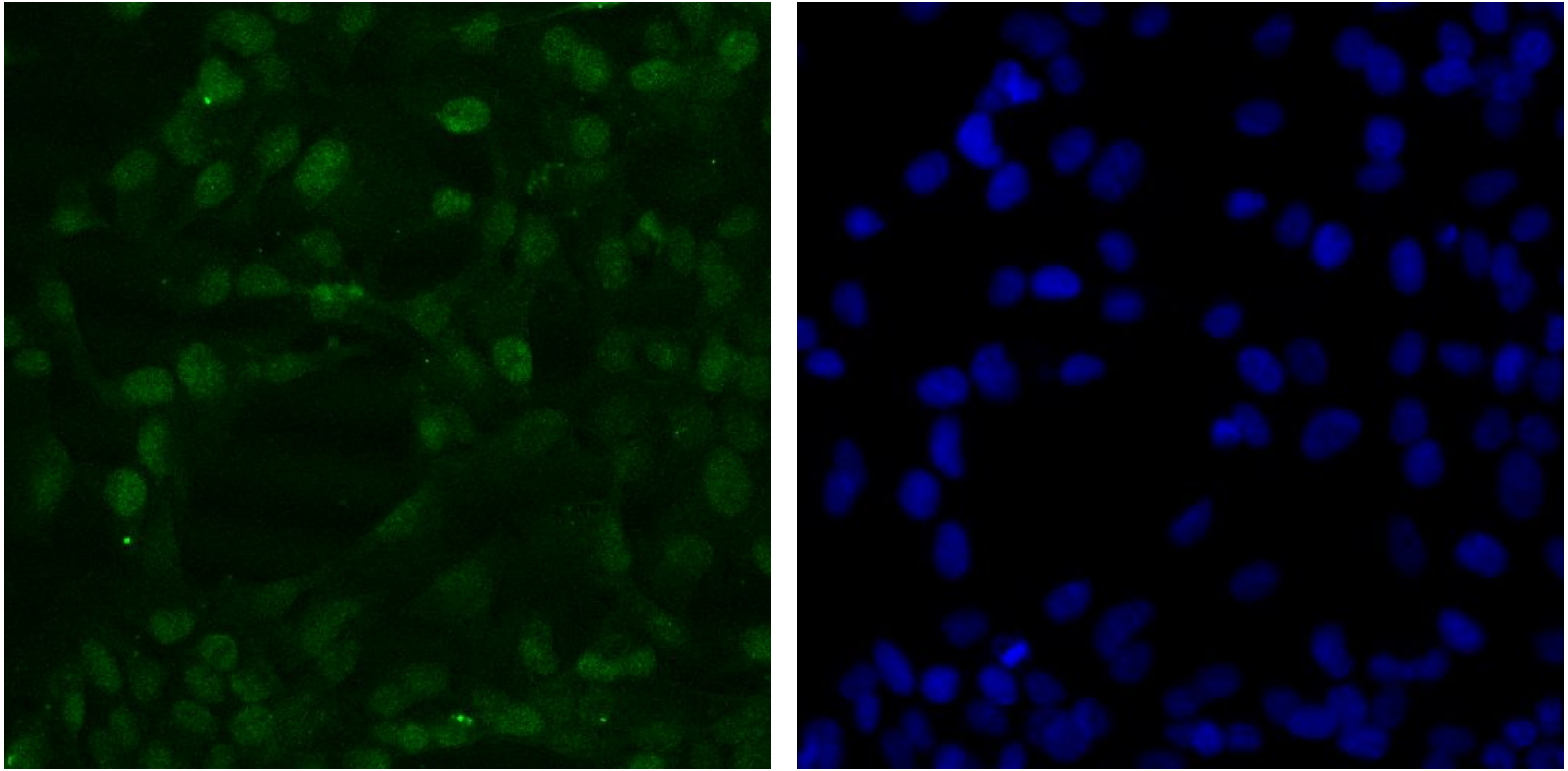

MDA-MB-231 - DNMT3A - siROR1-2

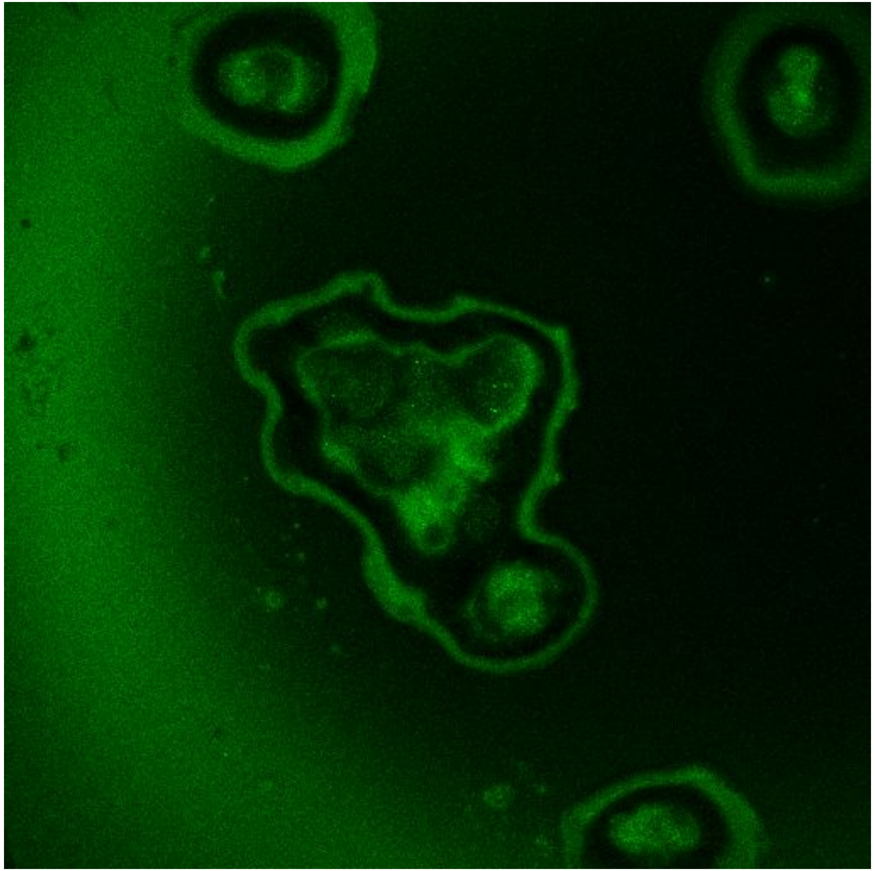

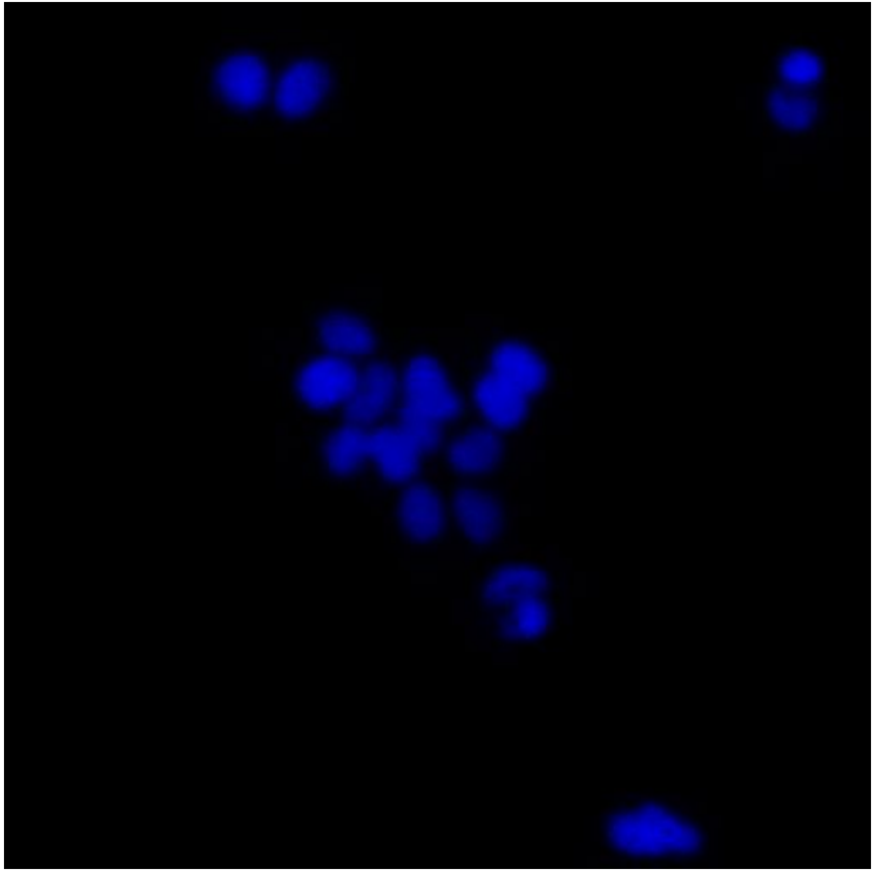

MDA-MB-231 - DNMT3A - siROR1-3

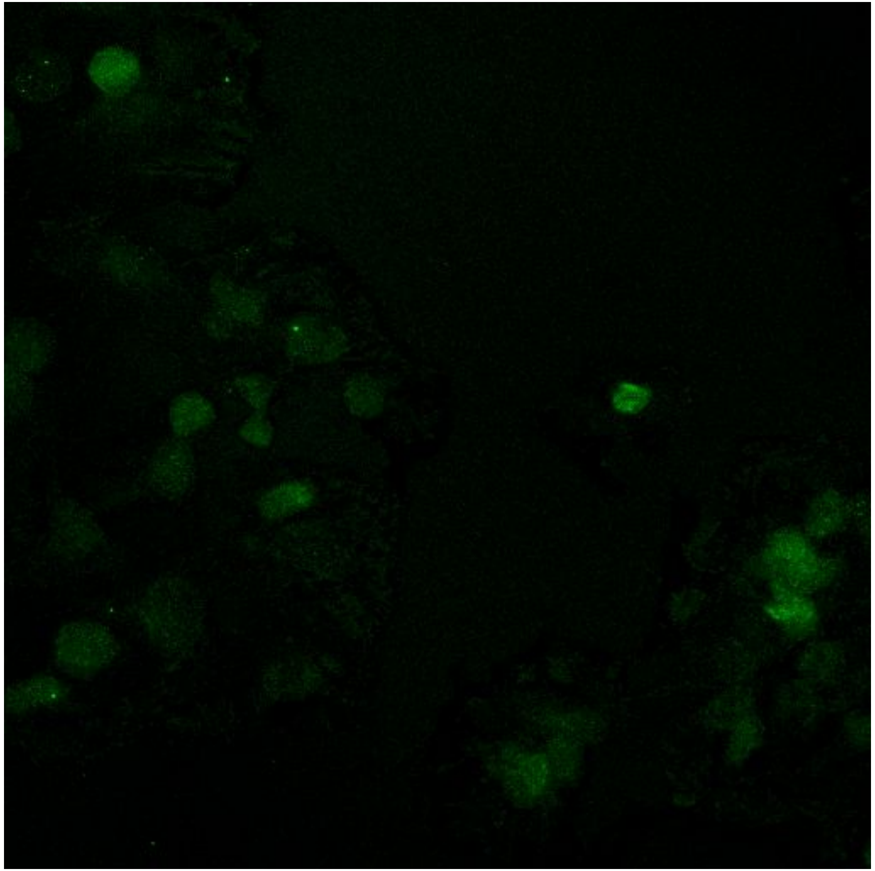

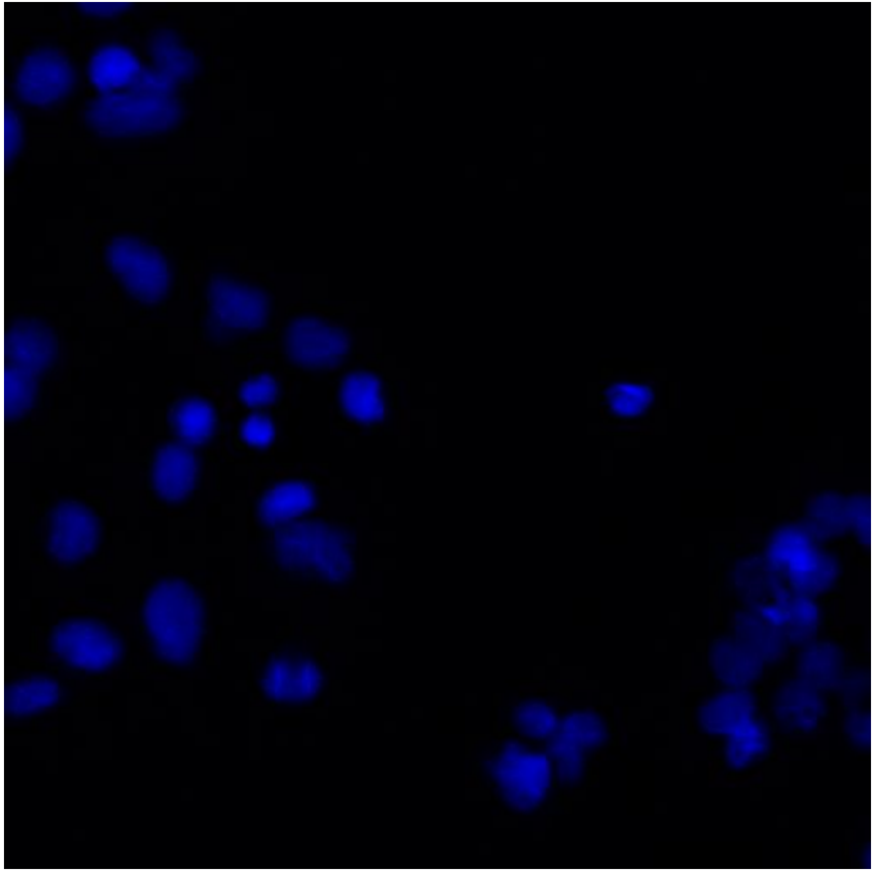

Supplement: Supplementary file 1 [file biomolecules-15-00734-s001.zip › biomolecules-3571225-supplementary.pdf]
